# Reduced-DSP 200G/λ low-voltage slow-light silicon modulator enabled by built-in EO equalizer

**DOI:** 10.1038/s41467-026-78128-1

**Published:** 2026-10-22

**Authors:** Keisuke Kawahara, Tai Tsuchizawa, Noritsugu Yamamoto, Yuriko Maegami, Koji Yamada, Nobuhiko Nishiyama, Toshihiko Baba

**Affiliations:** 1 Department of Electrical and Electronic Engineering, Institute of Science Tokyo, Tokyo, Japan; 2 Department of Electrical and Computer Engineering, Yokohama National University, Yokohama, Japan; 3 National Institute of Advanced Industrial Science and Technology (AIST), Tsukuba, Japan; 4 Photonics Electronics Technology Research Association (PETRA), Tokyo, Japan; 5 Institute of Advanced Sciences (IAS), Institute of Multidisciplinary Sciences (IMS), Yokohama National University, Yokohama, Japan

**Keywords:** Electrical and electronic engineering, Optics and photonics, Slow light

## Abstract

Multi-Tb/s optical interconnects for artificial intelligence data centers necessitate high-speed, energy-efficient, and compact transceivers. However, conventional silicon Mach-Zehnder modulators are fundamentally limited by low modulation efficiency and large physical dimensions that typically extend several millimeters, requiring high-swing drivers and power-intensive digital signal processing to achieve data rates exceeding 100 Gb/s per lane. Here we show a silicon photonic crystal waveguide slow-light modulator that overcomes the bandwidth-efficiency trade-off via a built-in electro-optic equalizer. By monolithically integrating optical delay lines and crossings within segmented slow-light phase shifters, we synthesized an on-chip feedforward response that extends the 3-dB bandwidth. The fabricated modulator with a small device length of 320 µm demonstrated 140-Gb/s on-off keying operation with an energy consumption of 26 fJ/bit using drive voltages compatible with fin field-effect transistor-based complementary metal-oxide-semiconductor technology. Furthermore, four-level pulse amplitude modulation achieved 180 Gb/s without digital signal processing and 200 Gb/s with a low-complexity 11-tap digital equalizer. By reducing reliance on power-dissipating electronics, this strategy provides a potential route toward future dense multi-Tb/s co-packaged optics for energy-constrained switching systems.

## Abstract (web summary)

Silicon photonic modulators are central to future high-capacity optical interconnects but face a trade-off between efficiency and bandwidth. This work addresses that challenge using a slow-light modulator with an integrated EO equalizer, enabling 200 Gb/s operation.

## Introduction

The transition to multi-Tb/s data-center interconnects for the AI era necessitates massive transceiver parallelism, positioning silicon (Si) photonics as a primary candidate^1–3^. Despite the commercial prevalence of Si Mach–Zehnder modulators, scaling the operating speed while minimizing power and footprint remains a challenge^4,5^. This difficulty stems from the low modulation efficiency of carrier-depletion p-n-doped phase shifters, which typically have a *V*_π_*L* of 2–5 V·cm, requiring millimeter-scale electrodes and high drive voltages^6–8^. These long electrodes incur severe RF propagation loss, which, combined with the RC time constant and electro-optic (EO) phase mismatch, limits the bandwidth to <50 GHz. To compensate for these limitations, state-of-the-art transceivers rely on stand-alone high-swing drivers^9–13^ and complex digital signal processing (DSP) with nearly 100-tap equalizers^14–20^. Since these components consume Watt-level power^21–24^, the primary challenge is to achieve 200 Gb/s/λ while minimizing dependence on such electronics.

Slow-light phase shifters address these efficiency constraints by exploiting enhanced light-matter interaction^25,26^. Photonic crystal waveguides (PCWs) exhibit a high group index, *n*_g_ > 20, which reduces *V*_π_*L* and device length to approximately 0.1 V·cm and 100 μm, respectively^27–29^. This has enabled complementary metal-oxide-semiconductor (CMOS) compatible low-voltage operation with energy consumption below 59 fJ/bit for the modulator alone^30^ and 0.78 pJ/bit even with a co-packaged driver^31^. In such short phase shifters, RF propagation loss is suppressed, and the operating speed is primarily determined by EO phase matching and the RC time constant^32^. However, previous demonstrations have shown that although EO phase-matched electrodes expanded the bandwidth, the RC time constant remained a bottleneck, limiting *f*_3dB_ to <40 GHz and the data rate to 64 Gb/s OOK^30–33^. Meanwhile, grating-based slow-light modulators have demonstrated broad bandwidths beyond 100 GHz along with 200 Gbaud pulse amplitude modulation 4-level (PAM4) operations^34,35^. However, their lower *n*_g_ results in a relatively higher *V*_π_*L* of 0.82 V·cm and a *V*_π_ of several tens of volts, necessitating high-power standalone drivers. Furthermore, high-speed operation relies heavily on computationally intensive neural-network-based DSP equalization, without which performance is limited to 112 Gb/s OOK even with *V*_pp_ = 5 V. Consequently, a Si slow-light modulator that simultaneously achieves low-voltage driving and high-speed operation without relying on DSP has not yet been realized.

Built-in EO equalizers enable stand-alone bandwidth enhancement without DSP by using signal delays in traveling-wave modulators to implement finite-impulse-response (FIR) filters^36,37^. A Si Mach-Zehnder modulator has achieved DSP-free >100 Gb/s OOK by employing an EO equalizer^38^. However, maintaining modulation depth during EO equalization requires increasing the phase shifter length or the drive voltage, resulting in a millimeter-scale footprint and a 5-V drive voltage. In addition, frequency-dependent RF losses in these long p-n-loaded electrodes have distorted the equalizer response and hindered PAM4 operation. Thin-film lithium niobate modulators with an EO equalizer support 200 Gbaud multi-level modulation, but their centimeter-scale phase shifters pose a barrier to massive parallelism^39,40^. In contrast, applying the EO equalizer to slow-light modulators fundamentally transcends the conventional bandwidth-efficiency trade-off as their high modulation efficiency eliminates footprint and voltage constraints and ensures ideal equalizer characteristics for multi-level modulation.

**Fig. 1:**
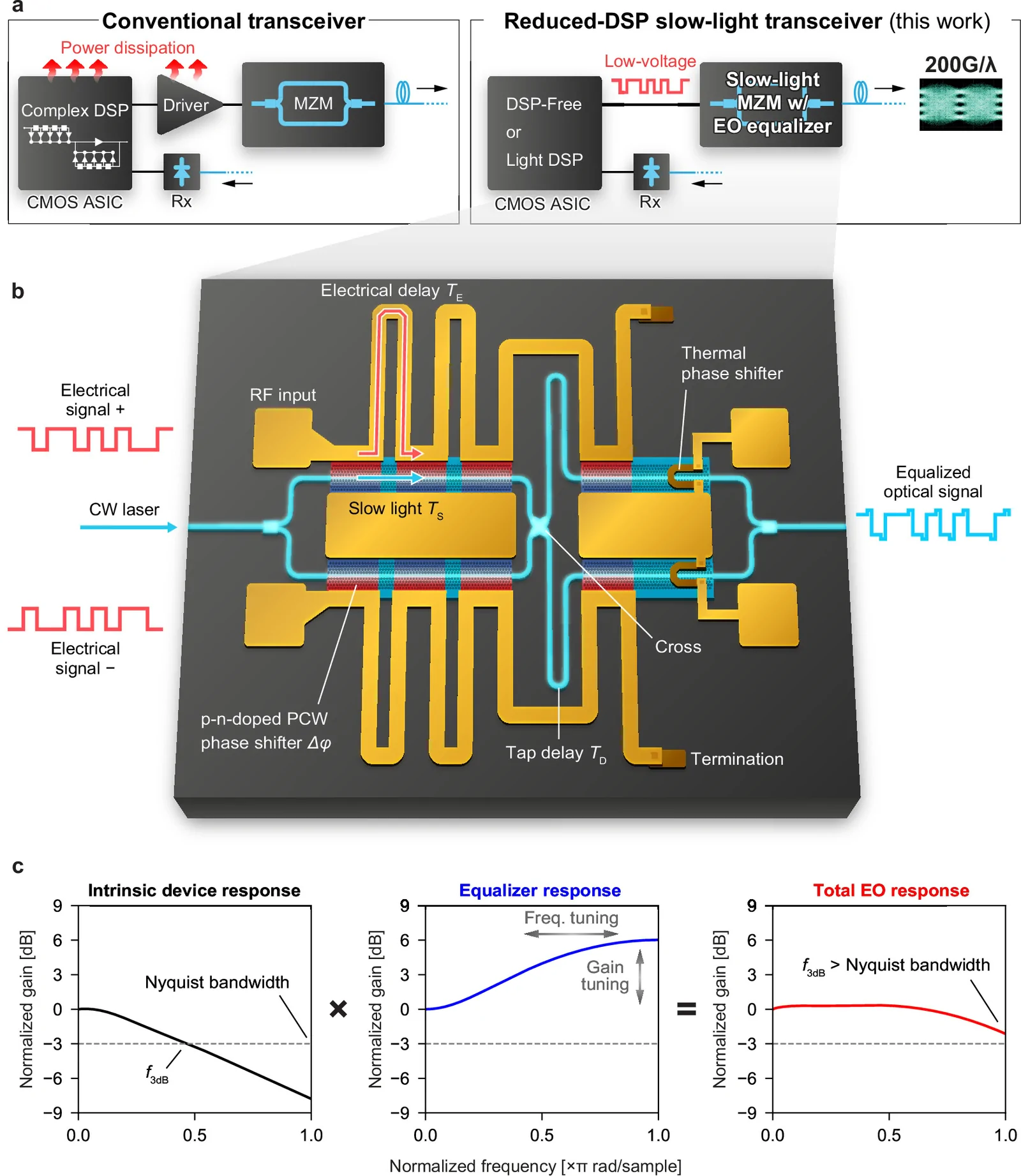
Concept of the reduced-DSP slow-light transceiver. **a** Comparison between a conventional transceiver architecture and the proposed architecture, designed for low-voltage 200 Gb/s/lane operation with minimal DSP requirements. **b** Schematic of the slow-light Mach-Zehnder modulator integrated with an EO equalizer. **c** Principle of bandwidth enhancement through the EO equalizer. The total electro-optic response is flattened by compensating for the intrinsic low-pass device response with the high-pass response of the equalizer, resulting in a 3-dB bandwidth that exceeds the Nyquist bandwidth.

In this paper, we present a Si PCW modulator that integrates a built-in EO equalizer to break the bandwidth limitation while preserving the benefits of the slow-light effect, as shown in Fig. 1. The equalizer compensates for the intrinsic roll-off with a high-pass response to extend *f*_3dB_. We demonstrated a Si modulator with a compact device length, while extending the equalized EO bandwidth beyond the Nyquist bandwidth required for 200-Gb/s PAM4. In modulation experiments using the device optimized for response flatness, we achieved 140-Gb/s OOK using FinFET-CMOS-compatible low-voltage driving, and 180-Gb/s PAM4 without DSP equalization. With the addition of low-complexity 11-tap DSP, we further reached 200-Gb/s PAM4 operation. From a modulator-design perspective, our proposal offers a path toward reducing reliance on power-dissipating DSPs and drivers in future multi-Tb/s co-packaged optics.

## Results

### EO equalizer concept

**Fig. 2:**
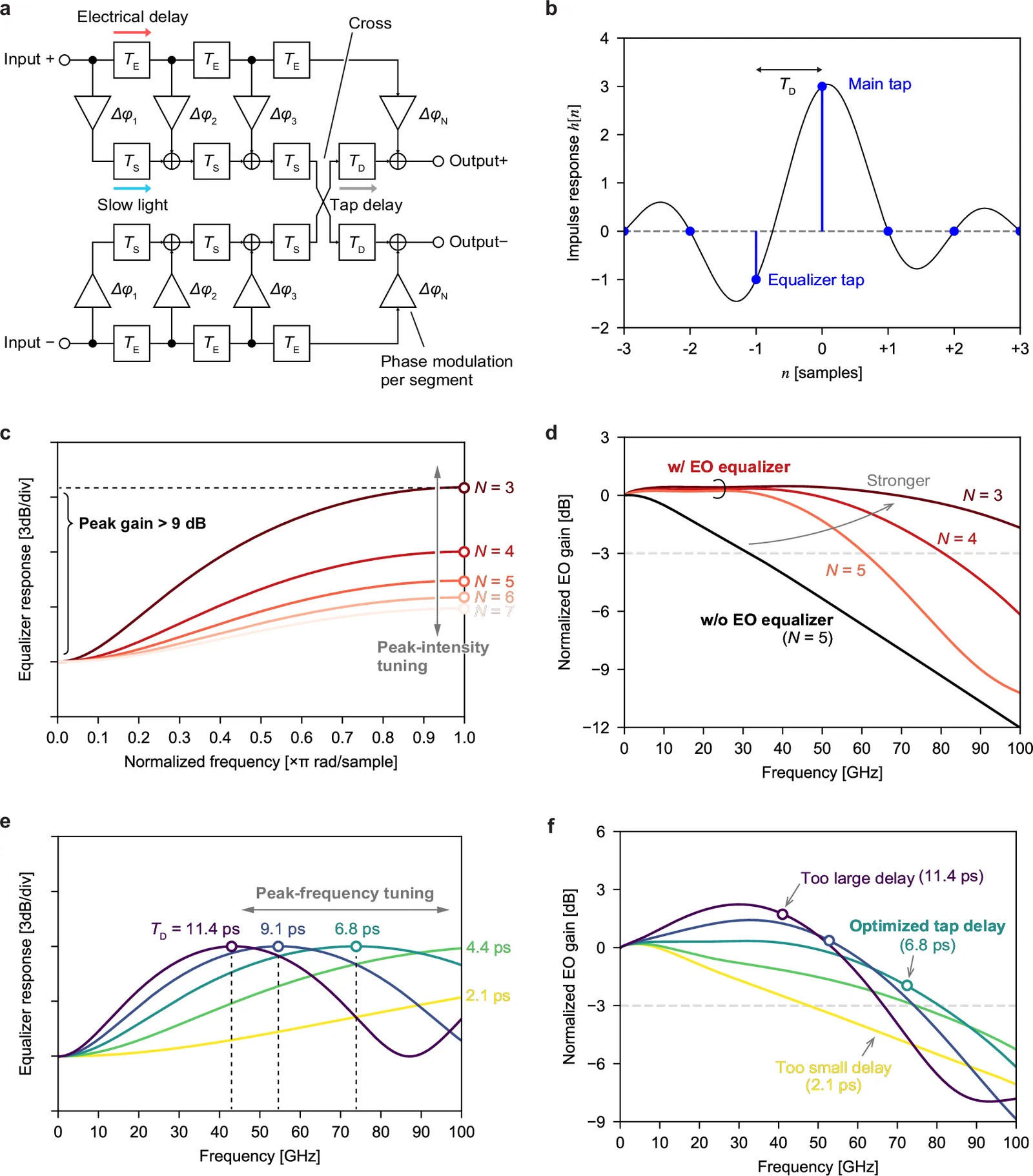
Design and optimization of the EO equalizer. **a** Block diagram of the equalizer. **b** Impulse response of the equalizer with its sinc interpolation (solid black line). **c** Equalizer response for varying segment count *N*, showing peak-intensity tuning with gains exceeding 9 dB. **d** Comparison of normalized EO gains for different *N* relative to the unequalized response. **e** Frequency response of the equalizer for various tap delays *T*_D_, demonstrating peak-frequency tuning. **f** Normalized EO gain for various *T*_D_ values at *N* = 4, identifying 6.8 ps as the optimized value for maximizing bandwidth while maintaining response flatness.

The block diagram of the built-in EO equalizer using a segmented Si PCW modulator is illustrated in Fig. 2a. The phase shifter was divided into *N* segments, with coplanar transmission lines inserted between segments. An optical delay line and an optical crossing were also inserted in front of the last segment, which shapes the frequency response. Each segment delays the electrical signal by *T*_E_ and the slow light by *T*_S_. The delay-line section applies *T*_D_ via a Si wire waveguide, and the crossing inverts the modulation. In the intended operating condition *T*_E_ = *T*_S_ and Δ*φ*_n_ = Δ*φ* (*n* = 1, …, *N*), the normalized impulse response is written as:

1 $$\frac{h\left(t\right)}{\Delta \varphi }={\sum }_{n=1}^{N-1}\delta \left(t\right)-\delta \left(t+{T}_{{{{\rm{D}}}}}\right)=\left(N-1\right)\delta \left(t\right)-\delta \left(t+{T}_{{{{\rm{D}}}}}\right).$$

All sections except the last one are delayed by *T*_D_ so that their phase shifts arrive in phase, whereas the inverted modulation from the last section is undelayed and therefore appears earlier relative to the others. This configuration realizes a two-tap feed-forward equalizer (FFE) with a main tap of amplitude (*N* − 1) at *t* = 0 and an equalizer tap of amplitude −1 at *t* = −*T*_D_, so that pulse shaping is performed without relying on conventional electronic DSP. Figure 2b shows the resulting impulse response for *N* = 4 and the corresponding continuous-time waveform obtained by convolution with a raised-cosine symbol pulse. The frequency response of the equalizer is obtained by the Fourier transform of Eq. (1) as:

2 $$\frac{H\left(\omega \right)}{\Delta \varphi }=\left(N-1\right)-{e}^{j\omega {T}_{{{{\rm{D}}}}}}.$$

The two parameters, *N* and *T*_D_, independently set the equalization strength and the peak frequency, respectively. Figure 2c shows the calculated equalizer gain for different *N*. Decreasing *N* increases the peak gain, and *N* = 3 provides more than 9 dB of high-frequency equalization. The corresponding EO gain in Fig. 2d shows that the bandwidth increases from 35 GHz without the equalizer to 61 GHz, 81 GHz, and >100 GHz at *N* = 5, 4, and 3, respectively. The peak frequency is determined by the relative phase, *ωT*_D_, between the main tap and the equalizer tap, as shown in Fig. 2e. As *T*_D_ increases, the same phase difference occurs at a lower *ω*, and the gain peak shifts to a lower frequency. A small *T*_D_ places the equalizer peak at an excessively high frequency, and the roll-off is undercompensated. In contrast, a large *T*_D_ shifts the peak to lower frequencies, resulting in overcompensation with an excess peak. For *N* = 4, *T*_D_ = 6.8 ps yields the flattest EO response, indicating that the equalizer peak effectively compensates for the intrinsic bandwidth limitation of the modulator. These design principles naturally extend to multi-tap configurations, where additional crossings and delay lines scale the equalizer to an arbitrary number of taps and increase design flexibility.

### Modulator implementation

**Fig. 3:**
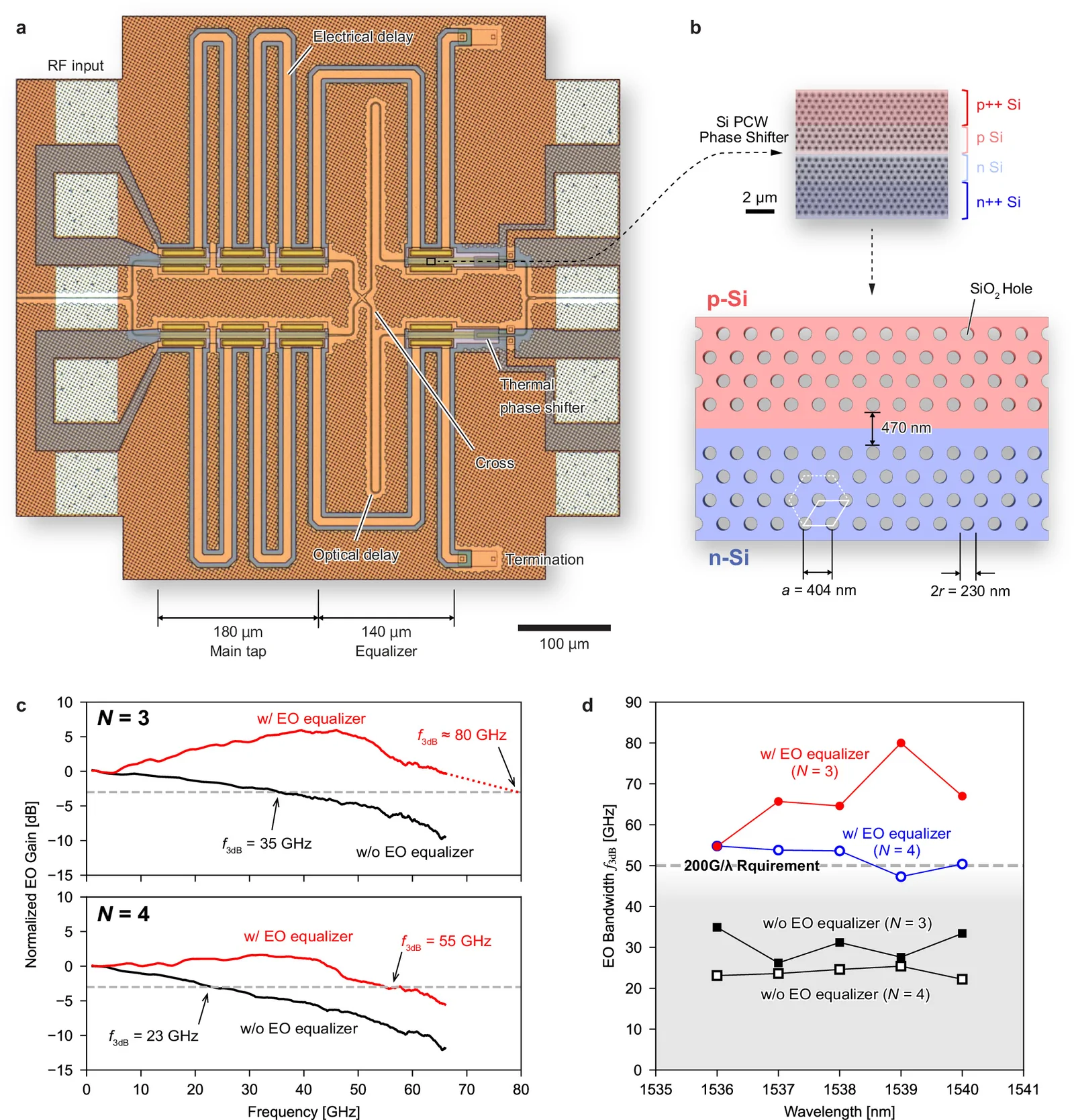
Fabricated modulator. **a** Optical micrograph. **b** Laser confocal micrograph and an enlarged view of the Si PCW phase shifter. **c** Measured normalized EO gain for *N* = 3 and 4 devices, demonstrating bandwidth enhancement from 35 to 80 GHz for *N* = 3 and from 23 to 55 GHz for *N* = 4 with the EO equalizer. The un-equalized responses were taken at 1536 nm to minimize the sinc roll-off from inter-segment EO phase mismatch, whereas the equalized responses were taken at the wavelength of maximum *f*_3dB_ within the 1536–1540 nm range. **d** Measured 3-dB bandwidth of the *N* = 3 and *N* = 4 devices across the operating wavelength range of the PCW.

We fabricated the PCW modulator in a 300-mm silicon-on-insulator (SOI) wafer foundry process, as shown in Fig. 3a. It is mainly composed of the segmented PCW phase shifters, an optical crossing, couplers, delay lines, coplanar electrodes, 50-Ω terminations, and thermo-optic heaters. The total footprint, including the electrodes, is only 0.4 mm². The active lengths are 180 µm for the main tap and 140 µm for the equalizer, including crossings and delay lines, yielding a total device length of 320 µm. For the PCW, the lattice constant and hole diameter are set to 404 nm and 230 nm, respectively, to obtain C-band light propagation, as shown in Fig. 3b. All structural dimensions exceed the minimum feature size of standard photolithography-based Si-photonics processes (~150 nm), ensuring tolerance to fabrication variations. The PCW provides *n*_g_ of 20–70, within which the measured *V*_π_*L* ranges from 0.51 to 0.16 V·cm (see Supplementary Note 1). Combined with moderately high p–n doping (*N*_A_ = 9.5 × 10¹⁷ cm⁻³, *N*_D_ = 1.0 × 10¹⁸ cm⁻³), the slow-light enhancement enables operation with only a few volts of drive despite the short interaction length. The phase shifters are divided into 50-µm segments, and 470-µm coplanar electrodes are inserted between segments to achieve simultaneous 50-Ω impedance matching and EO phase matching. This multi-segmented structure pushes the sinc-shaped roll-off caused by intra-segment EO phase mismatch beyond 100 GHz. With the roll-off shifted to such high frequencies, the equalizer can flatten the response with only a small peak gain (see Supplementary Note 2). Figure 3c shows the measured frequency responses of the *N* = 3 and *N* = 4 devices, with and without the EO equalizer (see Methods section for the experimental details). Consistent with the peak-intensity tuning discussed in Fig. 2c, the *N* = 3 device provided stronger high-frequency compensation and increased the 3-dB bandwidth from 35 to 80 GHz. The *N* = 4 device provided a more moderate equalization response, increasing the 3-dB bandwidth from 23 to 55 GHz with a flatter EO response. Figure 3d summarizes the measured 3-dB bandwidths of the *N* = 3 and *N* = 4 devices for the operating wavelengths of the PCW. Although the frequency response is slightly affected by the *n*_g_ dispersion of the PCW, a bandwidth exceeding 50 GHz was observed at almost all the wavelengths, satisfying the Nyquist criterion for 200-Gb/s PAM4 operation.

### Reduced-DSP 200G/λ operation

**Fig. 4:**
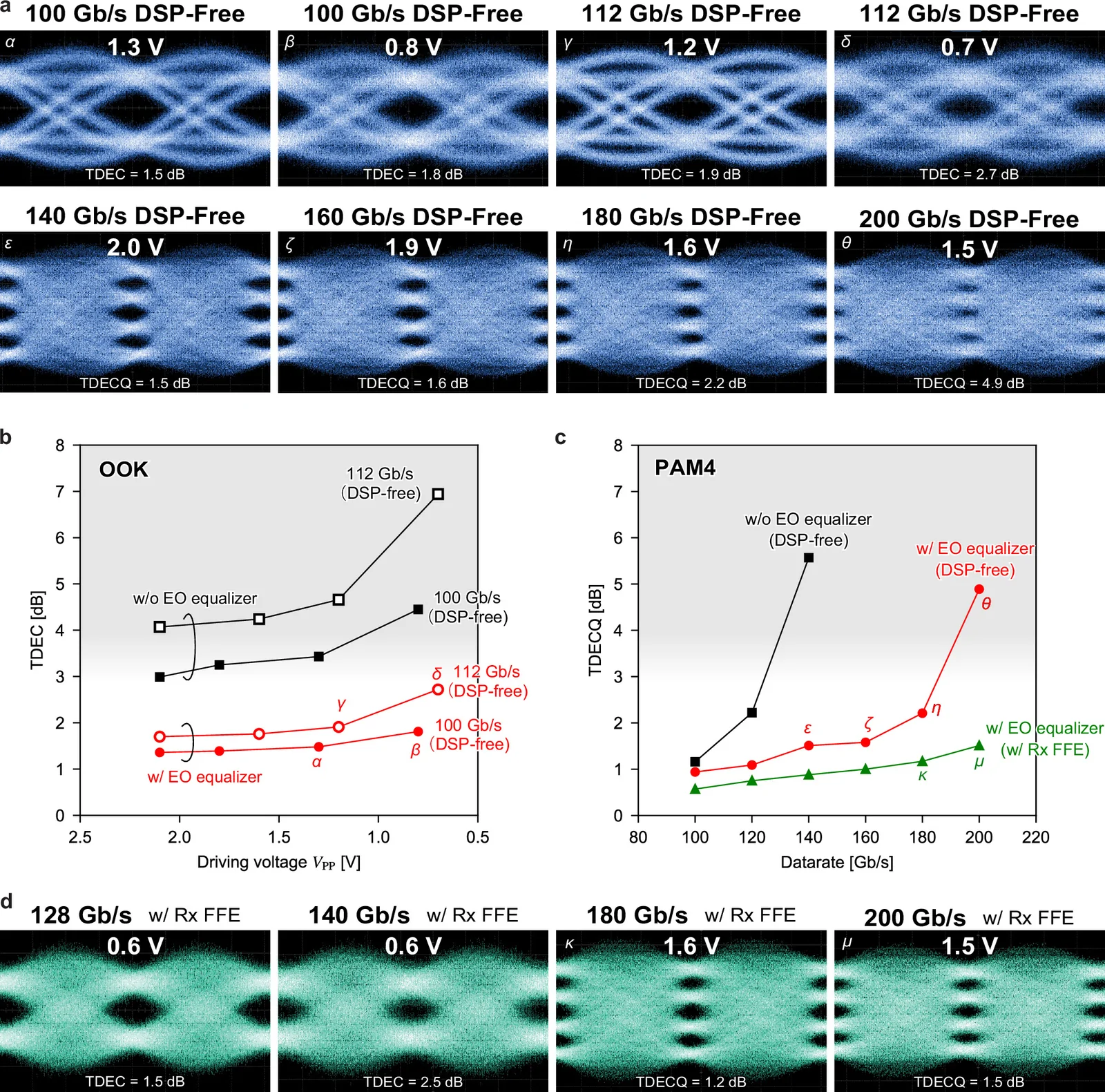
Measured optical modulation characteristics. **a** DSP-free eye diagrams for OOK (100–112 Gb/s) at *V*_pp_ = 1.3–0.7 V, *V*_DC_ = −2.5 V and PAM4 (140–200 Gb/s) at *V*_pp_ = 2.0–1.5 V, *V*_DC_ = −2.5 V. **b** Measured TDEC versus drive voltage for OOK signals. **c** TDECQ versus data rate for PAM4 signals up to 200 Gb/s. The shaded regions in (**b**) and (**c**) indicate typical limits for TDEC and TDECQ of approximately 3–4 dB. **d** Eye diagrams for 128–200-Gb/s OOK and PAM4 signals enabled by the EO equalizer and the low-complexity 11-tap Rx FFE.

To demonstrate high-speed optical modulation far beyond the intrinsic device bandwidth without relying on high-complexity DSP, we experimentally confirmed that the Si PCW modulator with a built-in EO equalizer operates up to 200 Gb/s PAM4. The modulation experiments were performed using the *N* = 4 device because of its flatter EO response, as shown in Fig. 3c. Figure 4a shows the measured eye diagrams, while Fig. 4b, c present the corresponding transmitter and dispersion eye closure (TDEC) and that for PAM4 (TDECQ) with and without the EO equalizer. All measurements used identical laser power and receiver settings (see the Methods section for experimental details).

We first benchmarked DSP-free OOK modulation under voltage levels compatible with 5-nm FinFET CMOS drivers^41^. As shown in the upper panel of Fig. 4a, clear eye openings at 100 Gb/s and 112 Gb/s were maintained down to *V*_pp_ = 0.8 V and 0.7 V, respectively. Without the EO equalizer, the modulator’s intrinsic bandwidth limitation caused TDEC values exceeding 3 dB across the entire voltage range, with severe degradation around *V*_pp_ ≈ 0.7 V. In contrast, applying the EO equalizer suppressed the increase in TDEC in the whole voltage range. Even under the lowest-voltage condition, the TDEC stayed as low as 1.8 dB at 100 Gb/s with *V*_pp_ = 0.8 V and 2.7 dB at 112 Gb/s with *V*_pp_ = 0.7 V. At this point, the energy required to drive the modulator corresponds to 43 fJ/bit. The lower panel of Fig. 4a demonstrates that the EO equalizer enables clear PAM4 eye openings at 140–200 Gb/s. As shown in Fig. 4c, without the EO equalizer, the TDECQ rapidly exceeds acceptable limits above 100 Gb/s because the intrinsic modulator bandwidth violates the Nyquist bandwidth. Due to this severe signal degradation, TDECQ could not be evaluated above 160 Gb/s. With the EO equalizer, the TDECQ was improved from 5.6 dB to 1.5 dB at 140 Gb/s and remained at or below 2.2 dB up to 180 Gb/s. Even at 200 Gb/s, although the TDECQ rose to 4.9 dB, a clear eye opening was still observed.

The data rate was further extended using a low-complexity 11-tap symbol-spaced FFE employed at the receiver, indicating the potential for practical optical interconnects. As shown in Fig. 4d, the achievable OOK data rate reaches 140 Gb/s at a low drive voltage of 0.6 V. The TDEC was improved to 1.5 dB at 128 Gb/s and 2.5 dB at 140 Gb/s, with a corresponding bit energy of 26 fJ/bit. For PAM4 modulation, the TDECQ was held to 1.5 dB even at 200 Gb/s with a corresponding bit energy of 63 fJ/bit. The remaining TDECQ margin suggests that higher data rates could be achieved under the same low-complexity FFE-only condition.

## Discussion

It has been recognized for years that Si photonics is a primary candidate for multi-Tb/s interconnects, yet the trade-off between modulation efficiency and operating bandwidth remains a fundamental bottleneck. As shown in Table 1, recent modulators have exceeded 200 Gb/s, although this progress has depended on high drive voltages^35,42,43^, long phase shifters^44,45^, and extensive DSP^14,17,35,42–47^. MRMs provide compact footprints and low bit energy, whereas their resonant operation requires wavelength control^17,46,47^. In this article, we have shown that integrating a built-in EO equalizer with a Si PCW slow-light modulator is effective for overcoming this trade-off while reducing reliance on DSP. The high modulation efficiency of the slow-light effect is critical for device miniaturization, which reduces the active length to the deep sub-millimeter scale. This short interaction length is vital for suppressing RF propagation loss, thereby allowing the integrated equalizer to maintain an ideal equalizer response for multi-level modulation. For this reason, we designed a segmented phase shifter with an integrated FIR filter and confirmed that the EO bandwidth increases beyond the Nyquist bandwidth. At the same time, the present modulator achieves the smallest *V*_π_*L* and the lowest drive voltage among those listed in Table 1, while retaining the non-resonant behavior. Following these designs, we have successfully demonstrated the DSP-free 180-Gb/s PAM4 operation using a Si slow-light modulator. We have also confirmed that the device achieves an energy consumption of 26 fJ/bit with a CMOS-compatible drive voltage and has achieved 200 Gb/s PAM4 using only low-complexity electronic assistance.

**Table 1:** Comparison with representative silicon modulators

Ref.	Type	Phase shifter	DSP-free data rate [Gb/s]	Data rate w/ DSP [Gb/s]	*V*_pp_ [V]	Driving bit energy^†^ [fJ/bit]	EO *f*_3dB_ [GHz]	Phase shifter length [mm]	Optical passband [nm]	*V*_π_*L* [V∙cm]	Insertion loss [dB]
This work	MZM	PCW	180 (PAM4) 112 (OOK)	200 (PAM4), 140 (OOK) 11-tap FFE	1.5 (PAM4) 0.6 (OOK)	63 (PAM4) 26 (OOK)	≈ 80 (*N* = 3) 55 (*N* = 4)	0.25^‡^ (*N* = 3) 0.32^‡^ (*N* = 4)	6	0.3	11.7 (*N* = 3) 15.5 (*N* = 4)
^35^	MZM	Bragg-grating	N.R.	400 (PAM4) bi-GRU/T-biGRU ANN equalizer	5	347	90	0.249	7	0.82	10.5
^14^	MZM	Rib	N.R.	350 (PAM4) 99FF/99FB-tap DFE	2.7	116	42	1.5	N.R.	1.7	3.6
^38^	MZM	Rib	140 (OOK)	N.R.	5	893	>110	0.9	N.R.	1.62	4.3
^42^	MZM	Rib	N.R.	100 (PAM4), 138 (PAM8) Pre-distortion + VNLE/NWF/BCJR	3.2	569 (PAM4) 318 (PAM8)	>35	2	N.R.	0.82	9.6
^43^	MZM	Rib	N.R.	106 (PAM4) Pre-emphasis + 21-tap FIR	4.5	531	7.7	1.6	N.R.	1.02	2.1
^44^	MZM	Rib	N.R.	Net 225 (PAM8) 2nd-order VNLE	2.3	72	47	2.5	N.R.	1.35	5.4
^45^	MZM	Rib	40 (PAM4)	46 (PAM4) FFE/NLE/MLSE	1.1, 2.1	611	27	3	N.R.	0.45	N.R.
^46^	MRM	Rib	N.R.	200 (PAM4) 21-tap FFE	1.6	6.3 (capacitive)	48.6	N.R.	≈ 0.35	0.6	6
^17^	MRM	Rib	N.R.	330 (PAM8), 280 (PAM-4) Pre-distortion/pre-emphasis + PNLE	1.8	3.1 (capacitive)	>50 (detuned)	N.R.	≈ 0.3	0.63	N.R.
^47^	MRM	Rib	N.R.	170 (PAM4) Pre-emphasis + 33FF/9FB-tap DFE	2.4	N.R.	47	N.R.	≈ 0.51	N.R.	N.R.

^†^ For MZM-based devices, driving bit energy was calculated from the drive voltage and *R*_T_ using the method described in Supplementary Note 5. *R*_T_ = 50 Ω was assumed when *R*_T_ was not reported.

^‡^ Including the built-in EO equalizer.

The achievement of such a high-speed and low-power-consumption modulator platform is important because it helps reduce reliance on power-intensive DSPs and external drivers. For practical CMOS driver implementation, the main constraints are the output swing and electrical bandwidth. In the present 200-Gb/s PAM4 experiment, the required drive swing at the modulator input was *V*_pp_ = 1.5 V, and the corresponding symbol rate was 100 Gbaud. This voltage swing can be handled by advanced CMOS drivers using cascode output stages to avoid device overvoltage stress^41^. The bandwidth requirement is also consistent with recent 5-nm FinFET driver demonstrations operating at 106.25 Gbaud^48^. At the same time, the present devices exhibit an insertion loss of 11.7–15.5 dB (see Supplementary Note 1), which was compensated by an erbium-doped fiber amplifier (EDFA) in the measurement setup. For future implementation in co-packaged optics, this loss needs to be reduced. The modulator architecture also provides a basis for future scaling through wavelength division multiplexing. In the present device, we used a simple line-defect triangular-lattice PCW, and the measured spectral window was 6 nm (see Supplementary Note 1). The available wavelength range can be broadened in future implementations either by integrating multiple PCW modulators, each designed to operate over a different wavelength range, or by using lattice-shifted PCWs with a broader spectral window. In particular, lattice-shifted PCWs have achieved a 35-nm bandwidth covering the C band^49^, suggesting a possible route to 16- to 32-channel integration with a projected total capacity of 3.2–6.4 Tb/s. Scaling the 0.4-mm^2^ modulator footprint to 32 channels would correspond to a projected total modulator area of 12.8 mm^2^, and the power consumption of the entire transmitter is projected to remain below several pJ/bit even after multiplexing. We believe that this architecture offers promise as a building block for future co-packaged optics in high-density data centers.

## Methods

### Design and fabrication

The modulator was designed using both device-level and circuit-level simulations. The structural parameters of optical components, including the multimode interference coupler, waveguide crossings, and the photonic-crystal waveguide, were determined by FDTD calculations (Lumerical FDTD). The doping profile of the p–n junction was optimized using a carrier-transport solver (Lumerical CHARGE), from which the RC time constants were extracted. The electrode parameters were designed using a method-of-moments solver (Keysight Momentum) to achieve the target impedance and electrical delay. The electrical delay line was designed with the coplanar-line geometry chosen to satisfy both impedance matching and EO phase matching^30^. Circuit simulations were performed in Keysight ADS using an in-house photonic device model library written in Verilog-A, enabling analysis of the EO frequency response and eye diagrams^50^. The model incorporates RC time constants, RF losses, EO phase mismatch, and optical loss. For the *N* = 4 device, the required time constants are *T*_E_ = *T*_S_ = 4.3 ps and *T*_D_ = 6.8 ps. The PCW length is 64.3 μm per segment, with a 50-μm active p–n region, providing *T*_S_ = 4.3 ps with *n*_g_ = 20. The electrical delay line is designed with a length of 470 μm to ensure EO phase matching. The optical delay line, which includes the waveguide crossings and transition tapers, has a total length of approximately 480 μm. The modulator was fabricated using the 300-mm SOI Si photonics process in the Super Clean Room (SCR) at the National Institute of Advanced Industrial Science and Technology (AIST). The silicon layer was patterned using ArF immersion lithography. The silicon ridge waveguide had a width of 450 nm, providing *n*_eq_ = 2.31, *n*_g_ = 4.34, and an optical loss of 2 dB/cm. After ion implantation, the devices underwent annealing, cladding deposition, TiN resistor formation, and finally aluminum electrode metallization.

### EO frequency response measurement

**Fig. 5:**
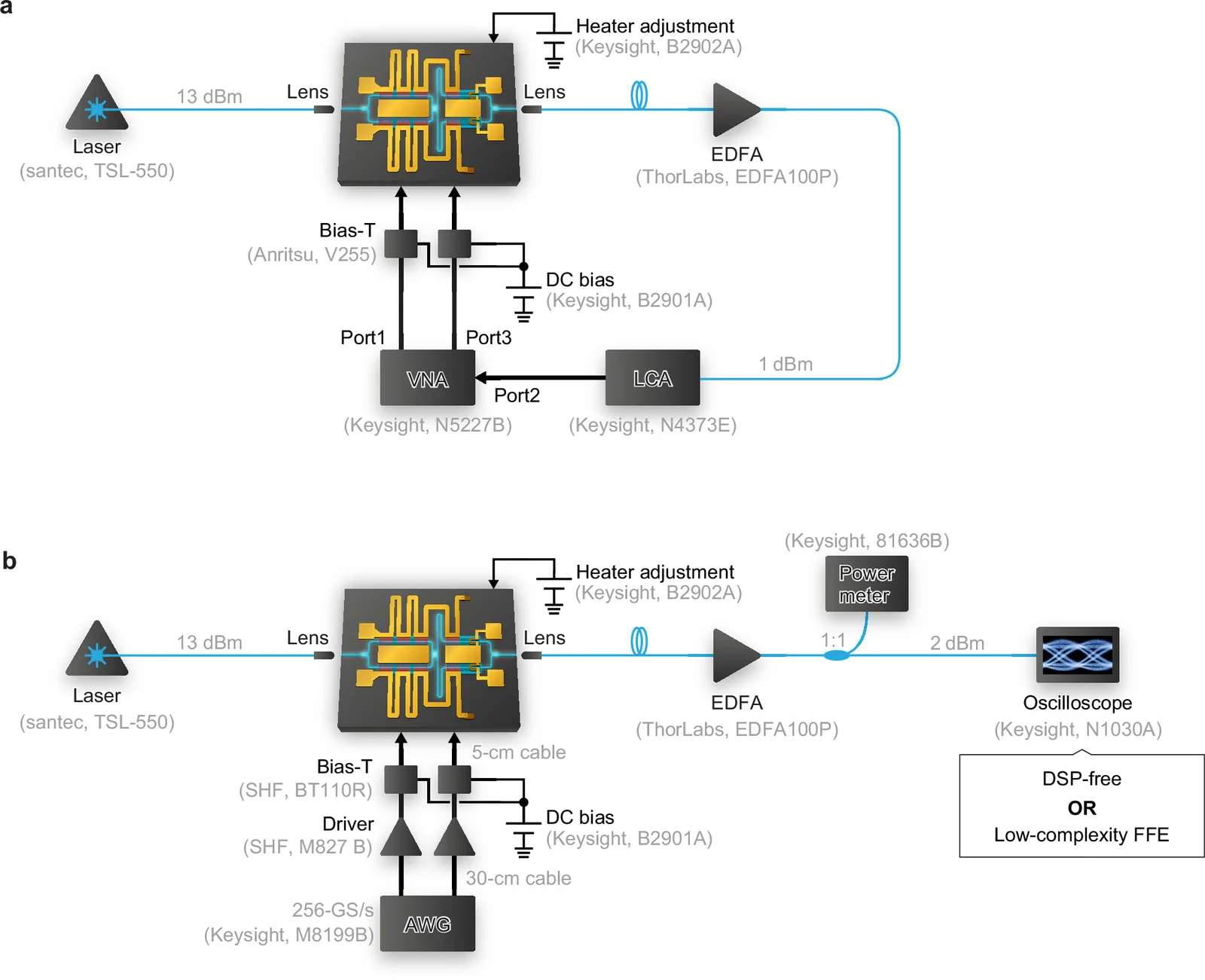
Experimental setups. **a** Small-signal EO response measurement setup consisting of a VNA and an LCA. **b** High-speed modulation measurement setup using an AWG and a sampling oscilloscope for evaluating reduced-DSP performance.

The EO frequency response was characterized using the setup shown in Fig. 5a. Transverse-electric (TE) polarized laser light at 13 dBm, generated using a tunable source, Santec TSL-550, was edge-coupled into the device via a lens-fiber module. The EO frequency response was measured over the wavelength range 1536–1540 nm. The electrical frequency response was measured using a vector network analyzer (VNA), Keysight N5227B, together with a lightwave component analyzer, Keysight N4373E LCA. The VNA’s IF bandwidth was set to 30 Hz. Ports 1 and 3 of the VNA were connected to the device’s differential input, with a port power of 3 dBm. A FormFactor 67-GHz Infinity GSGSG probe was used for electrical probing. The device’s optical output was detected by the LCA and fed into port 2 of the VNA. The p-n junction was biased at –3.5 V using Anritsu V255 65-GHz bias-tees. The modulator operating point was set to quadrature using an on-chip TiN heater. Port calibration of the VNA was performed using a Keysight N4694D ECal module. The electrical input network, consisting of the cables, bias-tees, and probe, was extracted using the short-open-load-thru (SOLT) method on a calibration substrate and de-embedded along with the internal response of the LCA. The output light was amplified to 1 dBm using a Thorlabs EDFA100P fiber amplifier. The measurement range was swept from 100 MHz to 67 GHz with a 100-MHz frequency step. The measured three-port S-parameters were converted to mixed-mode after measurement and smoothed with a 21-point window to suppress ripples. To estimate the response up to 80 GHz, a linear extrapolation was performed from the highest measured frequency of 67 GHz. The slope for this extrapolation was determined by a linear fit to the smoothed data in decibels over the last 10 GHz of the measurement range, from 57 to 67 GHz.

### Modulation experiments

The experimental setup for high-speed modulation is shown in Fig. 5b. Modulation experiments were performed using *N* = 4 devices. TE-polarized laser light at 1537 nm with an output power of 13 dBm was generated using the Santec TSL-550. The light was coupled into the device through a lens-fiber module and an edge coupler. An arbitrary waveform generator (AWG), Keysight M8199B 256-GS/s, generated electrical signals. After amplification using SHF M827B 70-GHz broadband amplifiers, the signals were delivered to the device through SHF BT110R 110-GHz bias-tees and a FormFactor 67-GHz GSGSG Infinity probe. The AWG in-system calibration compensated for the high-frequency roll-off of the electrical drive path by reducing the low-frequency gain. The external amplifiers were used to compensate for this amplitude reduction. The drive voltage applied to the modulator was controlled by adjusting the AWG output amplitude while keeping all other conditions fixed. The p–n junction of the modulator was biased at –2.5 V for both OOK and PAM4 modulation. 30-cm cables with 1-mm connectors and 5-cm cables with 1.85-mm connectors were used for the electrical interconnections. To suppress spurious signals, the signal bandwidth was limited by a raised-cosine roll-off filter inside the AWG. The timing mismatch between the differential signals was corrected by adjusting the AWG’s inter-channel delay. The modulator operating point was set to the quadrature point using an on-chip TiN heater. The modulated optical output was coupled to another lens-fiber module via an edge coupler. The Thorlabs EDFA100P fiber amplifier compensated for optical loss in the measurement setup. The amplified optical signal at 2 dBm was then directly launched into a digital communication analyzer (DCA), Keysight N1000A, equipped with a 65-GHz optical-to-electrical receiver module, Keysight N1030A. High-frequency losses in the electrical drive path, including the cables, drivers, and bias-tees, were compensated for up to 70 GHz using in-system calibration of the AWG. The intrinsic frequency response of the receiver module was corrected using a system-impulse-response correction filter. To suppress ASE noise from the EDFA, the acquired waveforms were averaged 16 times for OOK and 32 times for PAM4 modulation. These procedures were used only to correct the measurement-system response and were not used to equalize the modulator response. The optical power entering the receiver module was monitored using a power meter, Keysight 81636B, through a 1:1 fiber coupler. We used TDEC and TDECQ to compare the transmitter waveform quality with and without the EO equalizer under a common measurement procedure, thereby quantifying the reduction in waveform penalty achieved by the built-in EO equalizer. TDEC and TDECQ were evaluated using the FlexDCA software, with an SD-FEC threshold of *BER* = 2 × 10⁻² as the reference. While these metrics are suitable for transmitter characterization, evaluation of transceiver links will ultimately require BER-based measurements. For the DSP-free eye evaluation, no digital signal processing was applied. At higher symbol rates, a low-complexity symbol-spaced 11-tap FFE was applied on the DCA.

## Supplementary information

Supplementary information: Reduced-DSP 200G/λ low-voltage slow-light silicon modulator enabled by built-in EO equalizer

Dataset 1

Dataset 2

Transparent Peer Review file

## Data Availability

All data supporting the findings of this study are provided in the Article, Supplementary Information, and Source Data file.

## Code availability

The Verilog-A photonic device model library used for the circuit simulations is available on GitHub at https://github.com/keikawa/Verilog-A-photonic-model-library
