# Supplementary material for: Reduced-DSP 200G/λ low-voltage slow-light silicon modulator enabled by built-in EO equalizer
Source: Nat Commun. 2026 Oct 22;17:10446. doi: 10.1038/s41467-026-78128-1 (PMC13690699; doi:10.1038/s41467-026-78128-1)
Supplement: Supplementary file 1 [file 41467_2026_78128_MOESM1_ESM.pdf]

Supplementary information: Reduced-DSP 200G/λ low-voltage slow-light silicon modulator enabled by built-in EO equalizer

Keisuke Kawahara[1,2*], Tai Tsuchizawa[3], Noritsugu Yamamoto[3], Yuriko Maegami[3], Koji Yamada[3], Nobuhiko Nishiyama[1,4], and Toshihiko Baba[2, 5]

[1]*Department of Electrical and Electronic Engineering, Institute of Science Tokyo, Tokyo 152-8550, Japan*

[2]*Department of Electrical and Computer Engineering, Yokohama National University, Yokohama 240-8501, Japan*

[3]*National Institute of Advanced Industrial Science and Technology (AIST), Tsukuba 305-8568, Japan*

[4]*Photonics Electronics Technology Research Association (PETRA), Tokyo 112-0014, Japan*

[5]*Institute of Advanced Sciences (IAS), Institute of Multidisciplinary Sciences (IMS), Yokohama National University, Yokohama 240-8501, Japan*

***Corresponding author** Phone: +81-3-5734-3823, E-mail: keisuke@ee.eng.isct.ac.jp

Supplementary Note 1 – Optical Static Characteristics

Supplementary Figures 1(a) and 1(b) show the measured transmission spectra of the PCW modulator without and with the EO equalizer at $N = 3$. The total p-n-doped PCW phase shifter length is 150 μm. The measured on-chip insertion losses of the $N = 3$ device were 10.0 dB without the EO equalizer and 11.7 dB with the EO equalizer. For the $N = 4$ device used in the modulation experiment, the corresponding insertion losses were 13.4 dB and 15.5 dB, respectively. In this fabrication, the slab thickness decreased slightly due to process variations, which slightly raised the optical loss and shifted the spectral window toward shorter wavelengths. Nevertheless, the 6-nm spectral window is sufficiently wide to eliminate the need for temperature compensation. In our previous co-packaged transmitter experiment, driver-induced heating shifted the PCW spectrum by 2.6 nm, while the 3-dB operating bandwidth of the PCW was 5.9 nm. This result showed that a PCW modulator can operate without laser wavelength tuning or active temperature control when the spectral shift remains within the operating band [1]. The additional loss introduced by the equalizer is primarily caused by the transitions between the PCW and wire waveguides (approximately 0.4 dB × 2), as well as waveguide crossings and bends. Supplementary Figure 1(c) shows the measured n_g spectrum of the PCW, and Supplementary Figure 1(d) shows the evaluated $V_\pi L$ per phase shifter versus wavelength. The $V_\pi L$ values in

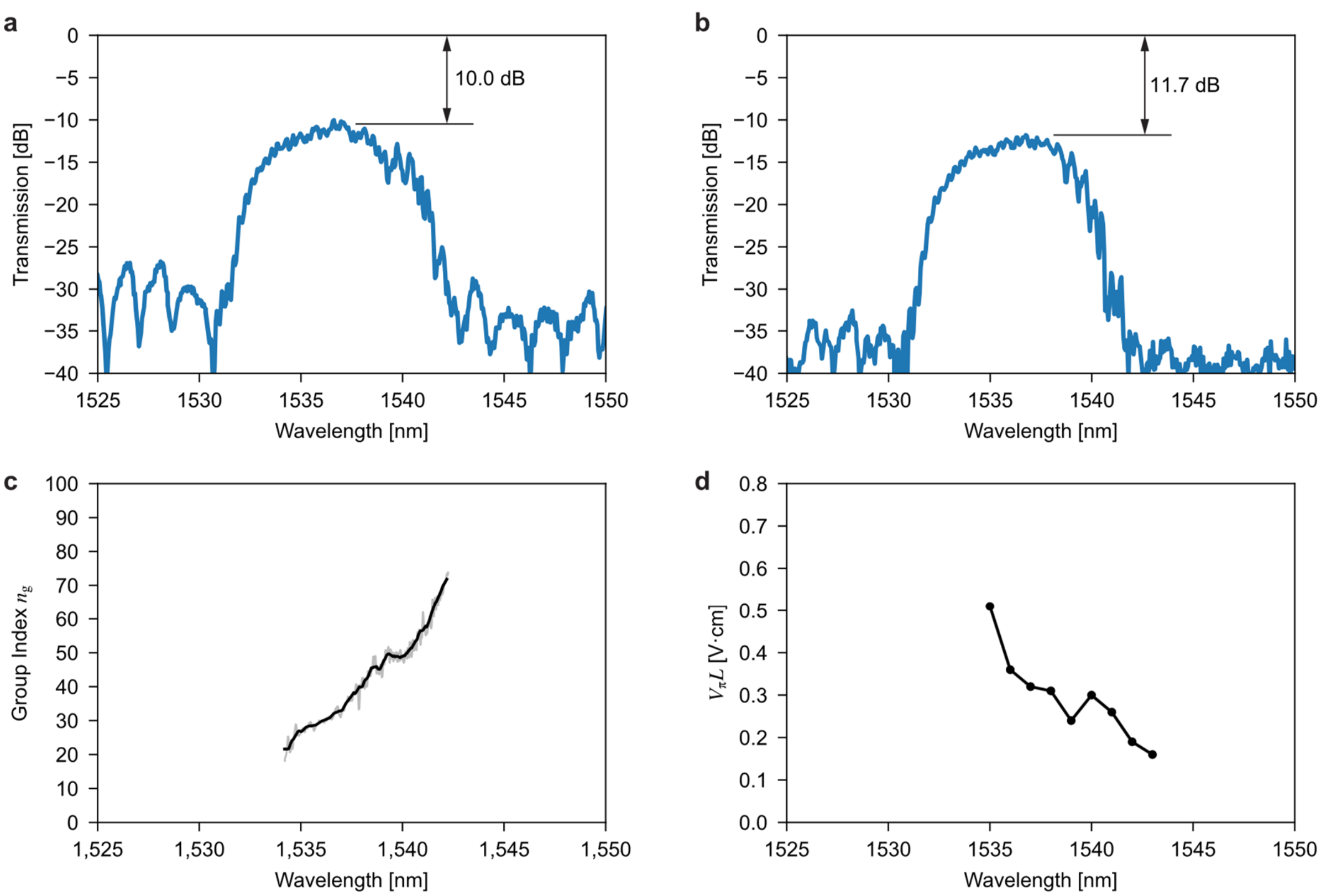

Supplementary Figure 1. Measured static characteristics of the PCW modulator. (a) Measured transmission spectrum of the PCW modulator without the EO equalizer at $N = 3$. (b) Measured transmission spectrum with the EO equalizer at $N = 3$. (c) Measured n_g spectrum of the PCW. (d) Measured $V_\pi L$ per phase shifter versus wavelength.

Supplementary Figure 1(d) were evaluated using the $N = 3$ device without the EO equalizer to characterize the intrinsic performance of the phase shifter. To avoid the influence of the frequency response, the extinction ratio was measured under OOK modulation at a low data rate of 2.4 Gb/s and quadrature bias with $V_{pp,diff} = 6.34$ V. We extracted V_π from the measured extinction ratio using the sinusoidal Mach–Zehnder transfer function, and then calculated $V_\pi L$ per phase shifter. The modulation efficiency improves at longer wavelengths due to the higher n_g, and the $V_\pi L$ reaches 0.16 V·cm at the longest wavelength, supporting CMOS-compatible low-voltage driving. The increase in n_g also causes carrier absorption in the p-n-doped PCW, which should be addressed by improving the PCW structure and doping condition. For example, periodic placement of the p-n junction can increase the overlap between the depletion region and the slow-light mode [2], thereby maintaining modulation efficiency even at lower doping levels.

Supplementary Note 2 – Frequency Responses

Supplementary Figure 2(a) shows the simulated intrinsic EO frequency response of the PCW modulator without an EO equalizer where the phase shifter length is fixed at 250 μm. The frequency response of the modulator without an equalizer is fundamentally determined by the RC time constant, the phase mismatch between the RF signal and slow light within a segment, and the propagation loss of the RF signal. The theoretical background is discussed in our previous paper [3], and the normalized electro-optic frequency response $G_{EO}(\omega)$ is expressed by

$$G_{\mathrm{EO}}(\omega) = \left|\frac{1}{1 + j\omega R_{\mathrm{pn}} C_{\mathrm{pn}}}\right| \cdot \frac{1 - e^{-N\alpha_{\mathrm{RF}} l_{\mathrm{U}}}}{N(1 - e^{-\alpha_{\mathrm{RF}} l_{\mathrm{U}}})}, \tag{1}$$

where R_{pn} is the resistance of the pn junction, C_{pn} is the capacitance of the pn junction, α_{RF} is the propagation loss of the RF signal, and l_U is the length of each segment. When N increases while the total length of the phase shifter remains constant, the length of each segment l_U becomes proportionally shorter. As indicated in Supplementary Equation 1, the roll-off caused by the phase mismatch between the RF signal and slow light within the segment depends on l_U. Therefore, the shortened segment length mitigates this phase mismatch. As a result, increasing N reduces the effect of the sinc roll-off in the high-frequency region, which improves the overall frequency response. Supplementary Figure 2(b) summarizes the simulated f_{3dB} and f_{6dB} as a function of N. The f_{3dB} saturates at $N = 2$ while the f_{6dB} is enhanced by increasing N. The more gradual roll-off and improved f_{6dB} at larger N values make the response easier to compensate with an EO equalizer and enable a broad bandwidth.

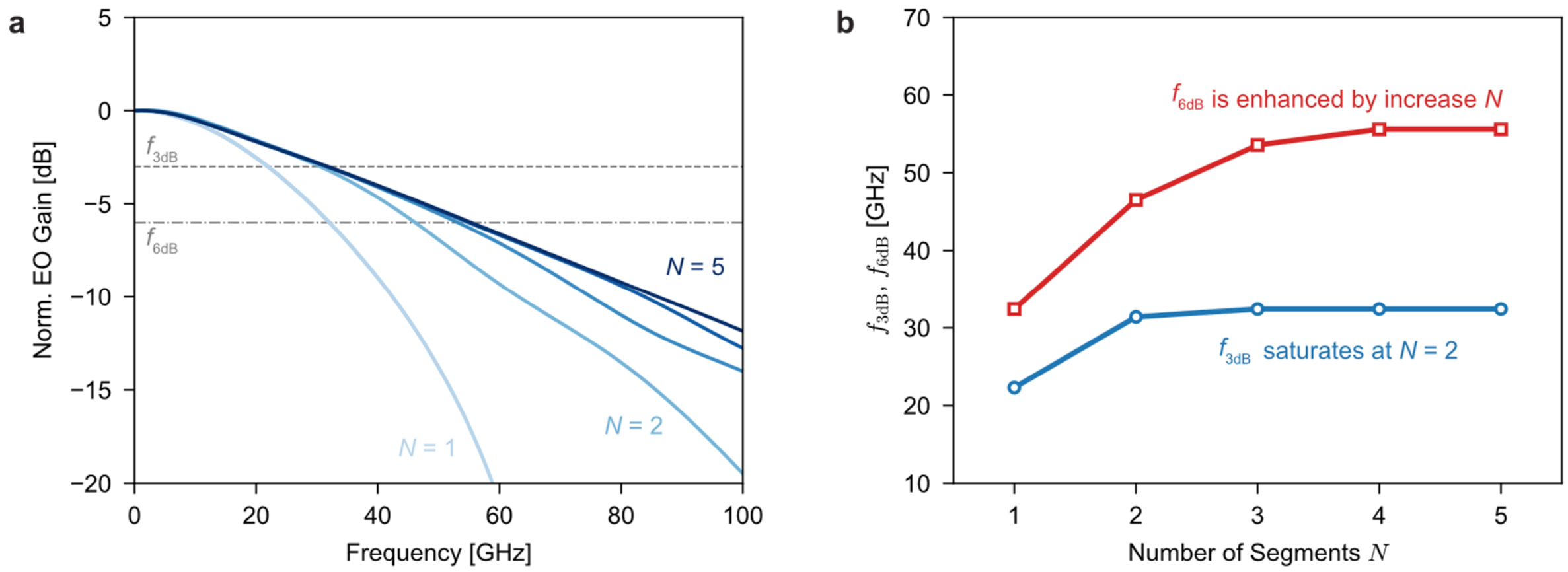

Supplementary Figure 2. Intrinsic EO frequency response of the modulator without an EO equalizer with the phase shifter length fixed at 250 μm. (a) Normalized EO gain for segment numbers N from 1-5, where increasing N mitigates the sinc roll-off effect. (b) Simulated f_{3dB} and f_{6dB} bandwidths as a function of N. The more gradual roll-off and improved f_{6dB} at larger N values make the response easier to compensate with an EO equalizer and enable a broad bandwidth.

Supplementary Figures 3 and 4 show the measured EO frequency responses of the PCW modulator using an EO equalizer for N = 3 and 4, respectively. Because the simple line-defect triangular-lattice PCW has a wavelength-dependent n_g, shifts of the n_g spectrum caused by fabrication variations or temperature drift change n_g at the operating wavelength. This changes the EO phase matching and the tap delay T_D of the equalizer, and consequently modifies the EO response. While phase mismatch typically narrows the bandwidth in conventional traveling-wave modulators, it can induce high-frequency peaking when an EO equalizer is employed. In Supplementary Figure 3, the bandwidth for $N = 3$ was maximized at 1539 nm, where the estimated f_{3dB} reached 80 GHz. The f_{3dB} exceeded 50 GHz at almost all the wavelengths for both N = 3 and 4, which supports 200 Gb/s PAM4 operation. The modulation experiment was conducted using the $N = 4$ device at 1537 nm, which was selected for its superior flatness. Changes in the EO response caused by fabrication variations and temperature changes can be mitigated by using lattice-shifted PCWs with a flatter n_g spectrum. For example, previous calculations showed that a lattice-shifted PCW designed around $n_g \approx 20$ can provide an almost flat n_g spectrum over a 35-nm wavelength range [4]. In PCW modulators, the lattice-shift design has also enabled athermal operation over 19–124°C [5]. Such a flatter n_g spectrum also helps preserve the EO-equalizer response.

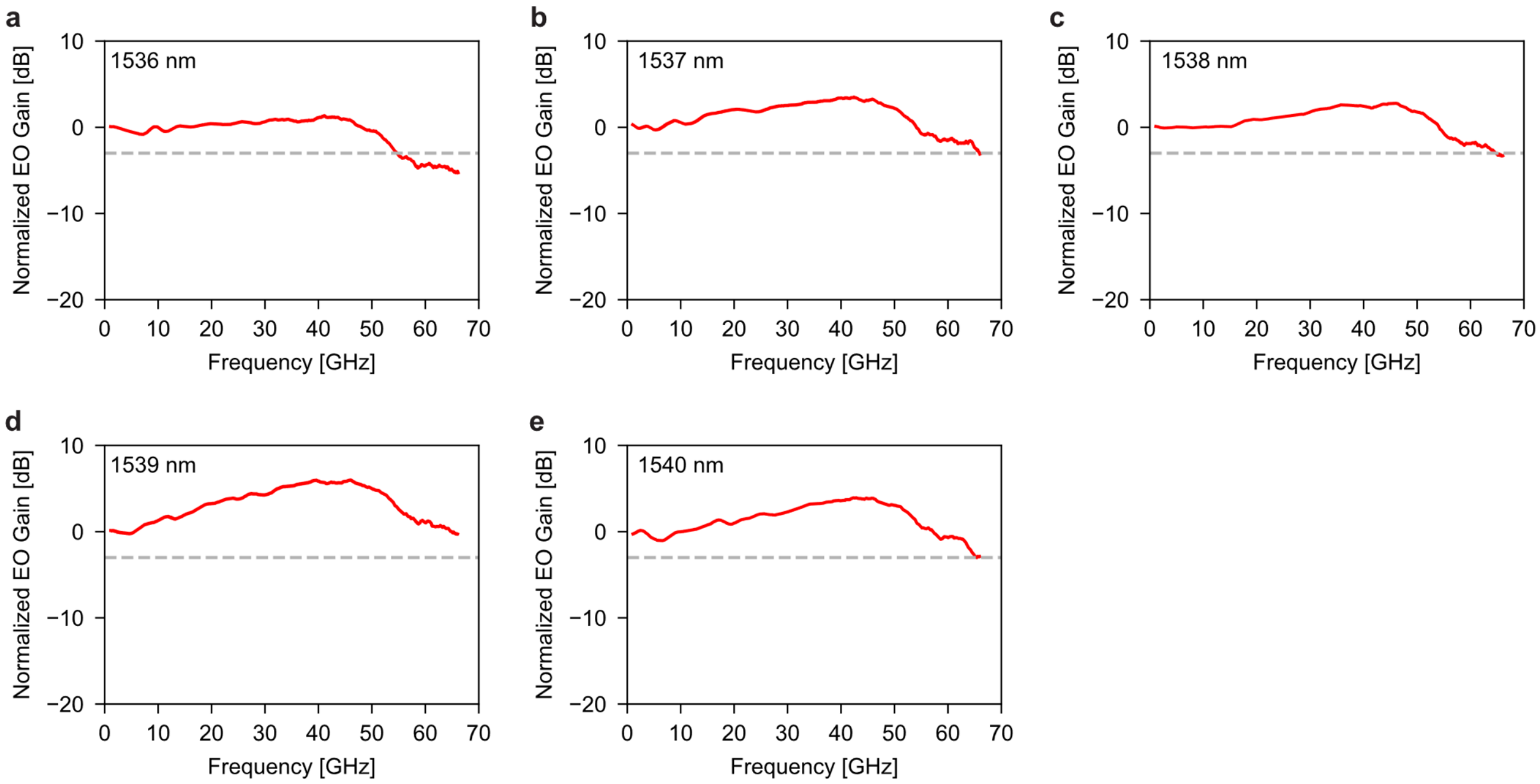

Supplementary Figure 3. Measured EO frequency response of the EO equalized PCW modulator (*N* = 3) for optical wavelengths λ ranging from (a) 1536 nm to (e) 1540 nm. The dashed grey lines indicate the 3-dB bandwidth threshold.

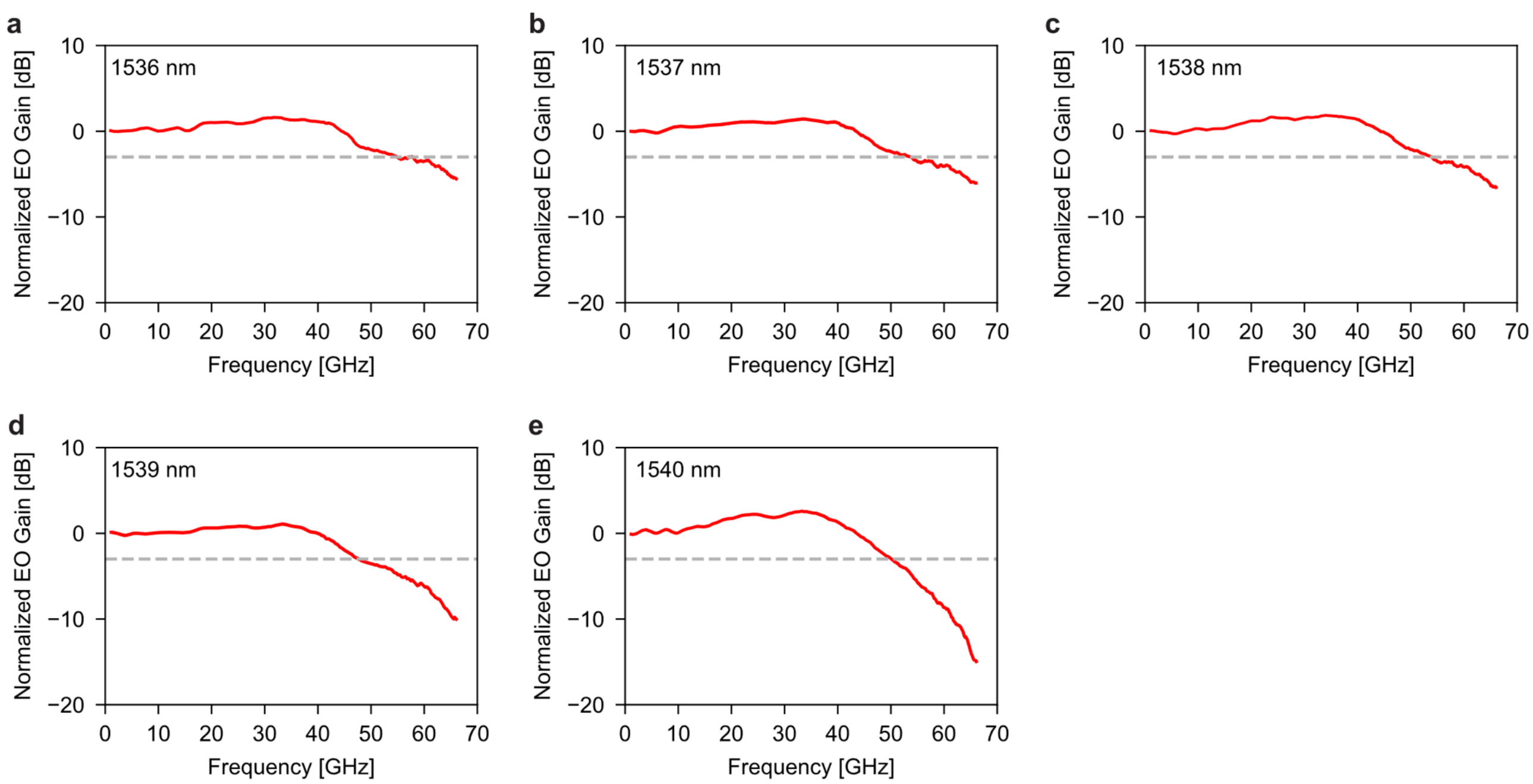

Supplementary Figure 4. Measured EO frequency response of the EO equalized PCW modulator (*N* = 4) for optical wavelengths λ ranging from (a) 1536 nm to (e) 1540 nm. The dashed grey lines indicate the 3-dB bandwidth threshold.

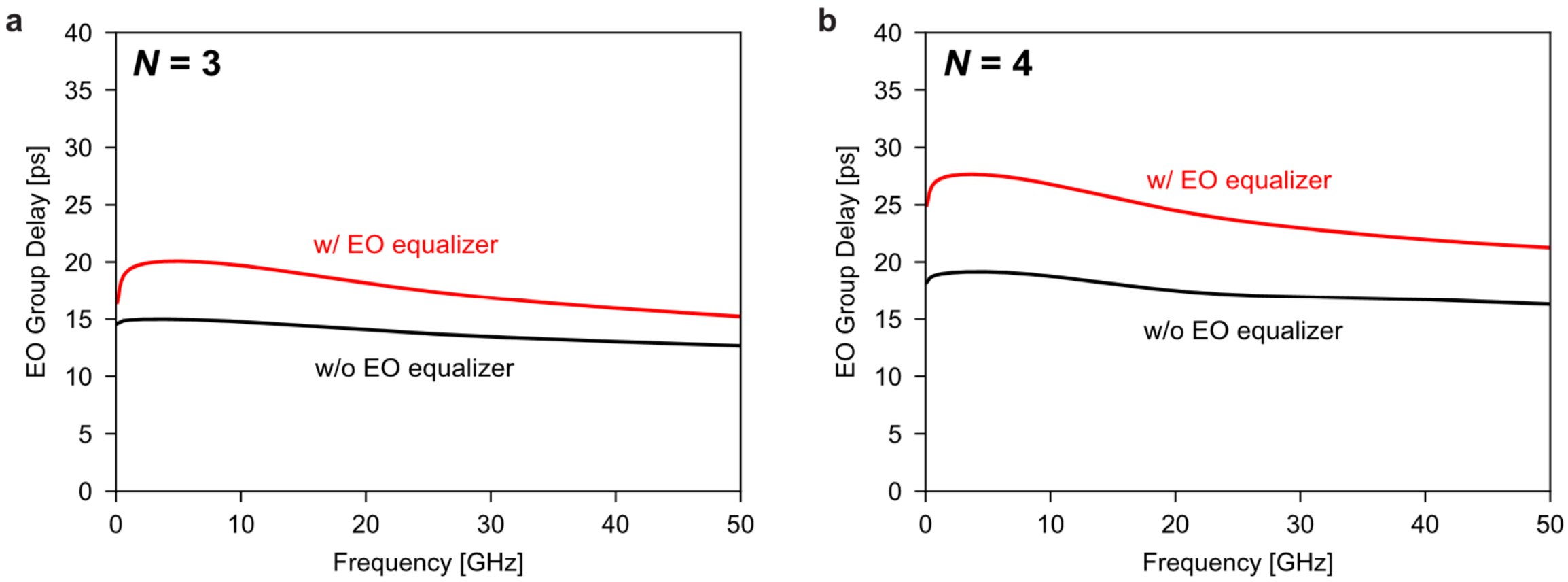

Supplementary Figure 5. Simulated EO group-delay frequency responses of the PCW modulators with and without the built-in EO equalizer for (a) N = 3 and (b) N = 4 devices.

Supplementary Figure 5 shows the simulated group-delay frequency responses of the $N = 3$ and $N = 4$ devices with and without the built-in EO equalizer. As discussed in our previous report [3], an appropriately designed Si PCW modulator intrinsically exhibits a favorable group-delay response. In our simulation, the Si PCW modulator without the built-in EO equalizer shows a small Nyquist-range group-delay variation of approximately ±1.2 ps for the $N = 3$ device and ±1.4 ps for the $N = 4$ device. The present built-in EO equalizer was designed primarily to reduce the high-frequency amplitude roll-off of the modulator. As a result, the Nyquist-range group-delay variation is somewhat increased to approximately ±2.4 ps for the $N = 3$ device and ±3.2 ps for the $N = 4$ device. However, the substantial reduction of the high-frequency roll-off suggests that the built-in EO equalizer provides a net benefit to the transmitter waveform quality, even when this increase in group-delay variation is considered. In addition, the effect of group-delay variation on the time-domain waveform, including pattern-dependent jitter, is reflected in the measured TDEC and TDECQ values by appropriately setting the histogram positions, as discussed in Supplementary Note 3.

Supplementary Note 3 – TDEC and TDECQ Measurement

Supplementary Figure 6 shows the TDEC and TDECQ measurement configurations used in this work. To evaluate the waveform quality while capturing the effect of time-domain waveform degradation, such as pattern-dependent jitter, we intentionally placed the histograms used for the TDEC and TDECQ measurements away from the center region of the eye pattern. Specifically, two 0.04-UI-wide histograms separated by 0.2 UI were used for TDEC measurements, whereas two 0.04-UI-wide histograms separated by 0.1 UI were used for TDECQ measurements. This histogram placement makes the TDEC and TDECQ values sensitive to timing jitter and other time-domain waveform degradations. Supplementary Figure 7 shows the TDEC and TDECQ results evaluated using the more conservative HD-FEC threshold of $BER = 3.8 \times 10^{-3}$. The results show that the built-in EO equalizer remains effective even under this stricter reference. In particular, for PAM4

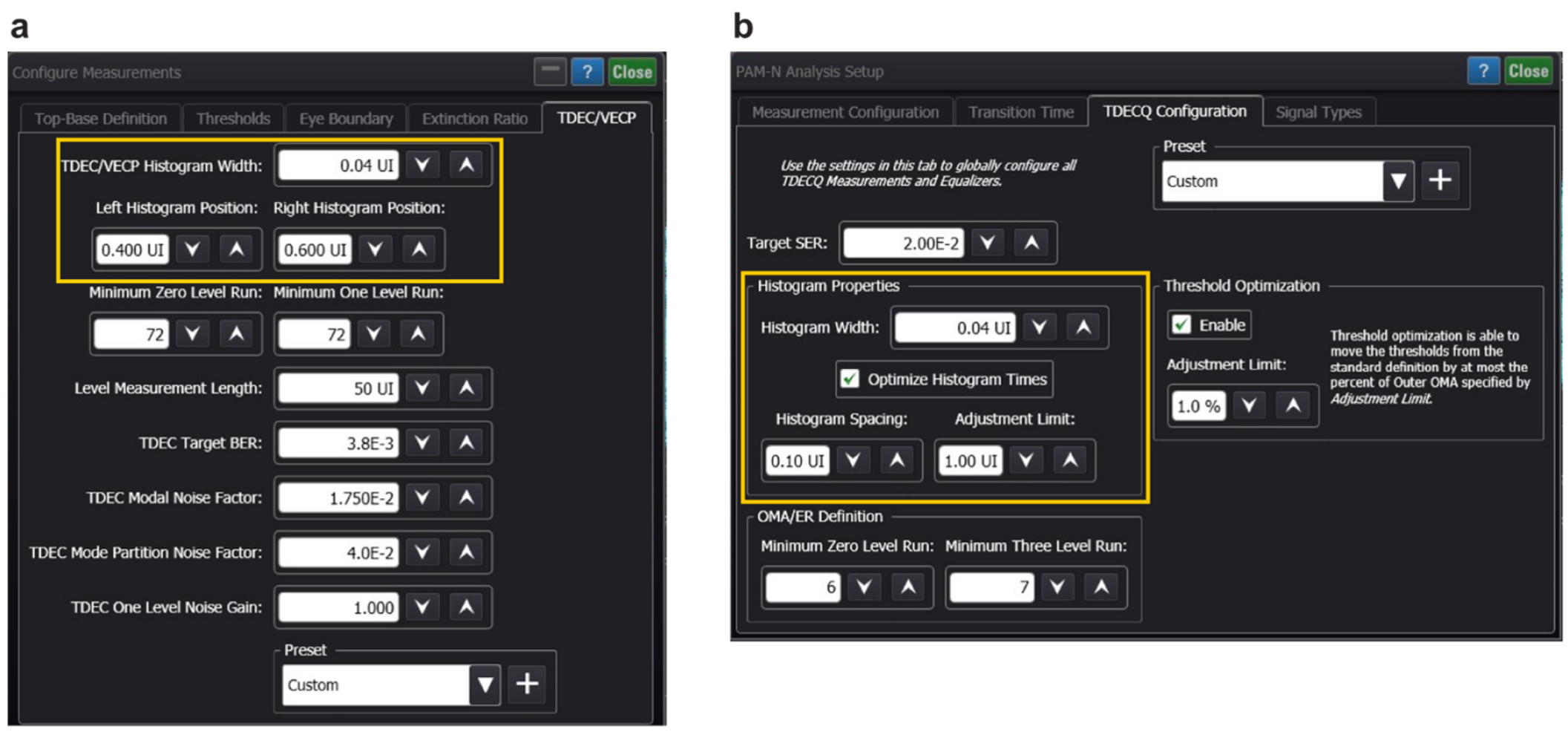

Supplementary Figure 6. Measurement configurations for (a) TDEC and (b) TDECQ. The yellow boxes indicate the histogram width and position settings.

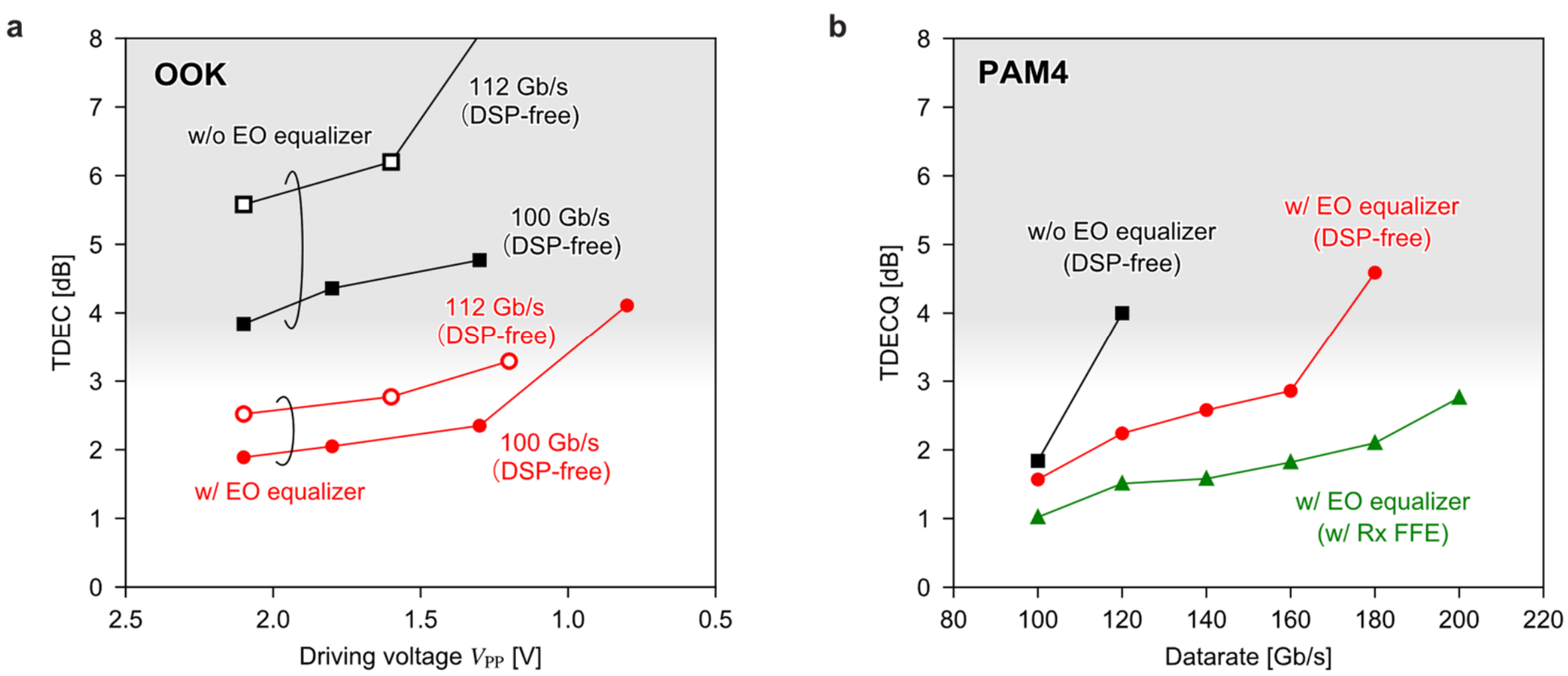

Supplementary Figure 7. TDEC and TDECQ evaluated using the HD-FEC threshold of $BER = 3.8 \times 10^{-3}$. (a) Measured TDEC versus drive voltage for OOK signals with and without the built-in EO equalizer. (b) TDECQ versus data rate for PAM4 signals. The shaded regions in (a) and (b) indicate typical limits for TDEC and TDECQ of approximately 3–4 dB.

modulation, TDECQ < 3 dB was maintained up to 160 Gb/s without DSP and up to 200 Gb/s with the 11-tap FFE.

Supplementary Note 4 – Electrical Eye Diagrams

Supplementary Figure 8 shows the measurement setup of the electrical drive signal waveform. In this setup, the output signal from the AWG (Keysight M8199B) passed through the driver amplifier (SHF M827B) and bias-T (SHF BT110R), and was then connected to a 20-dB attenuator (Anritsu 41V-20) through a 5-cm cable. The attenuated signal was then input to Channel 2A of the DCA through a remote sampling head (Keysight

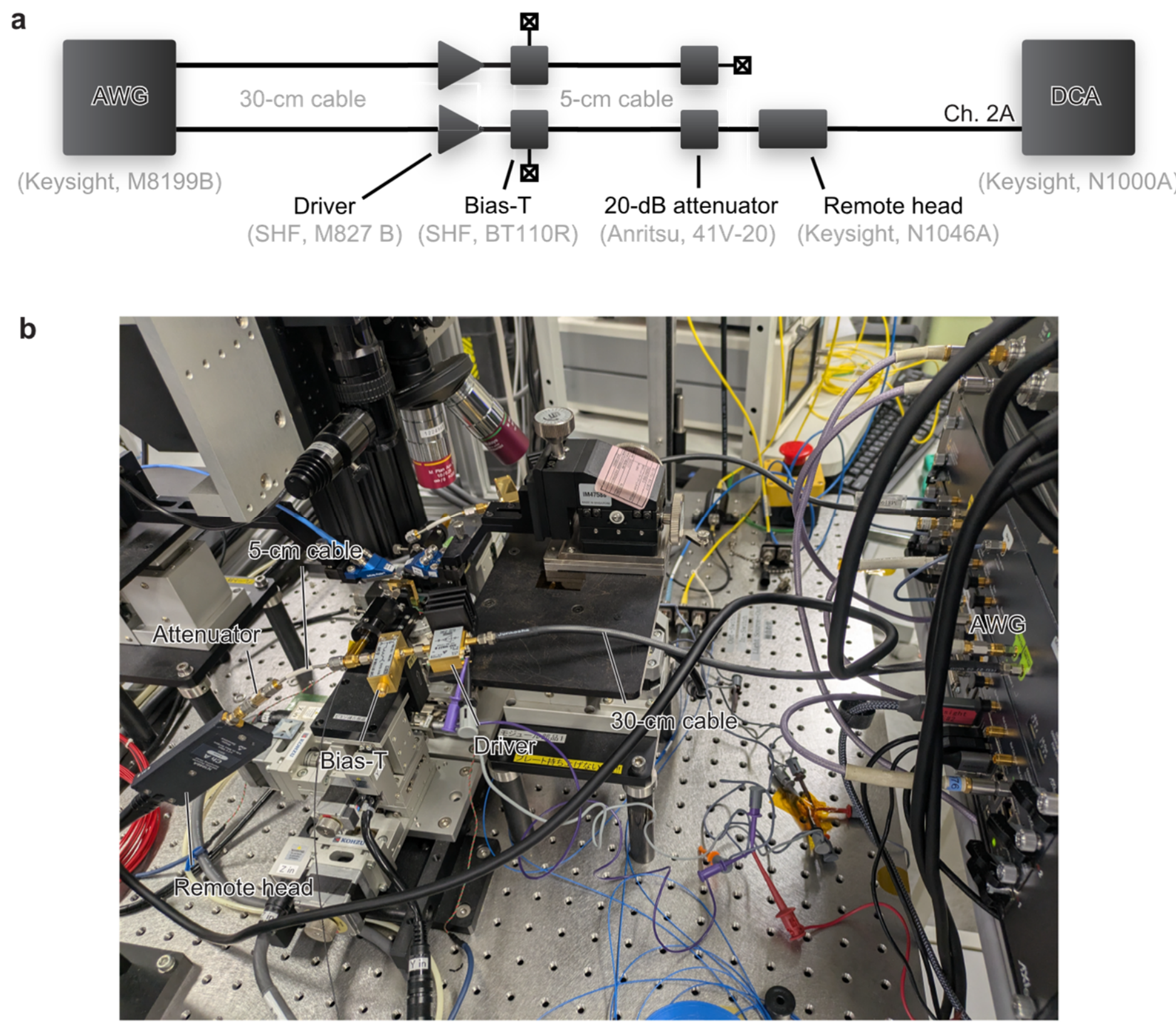

Supplementary Figure 8. Measurement setup for the electrical driving signal waveforms. (a) Block diagram. (b) Photograph.

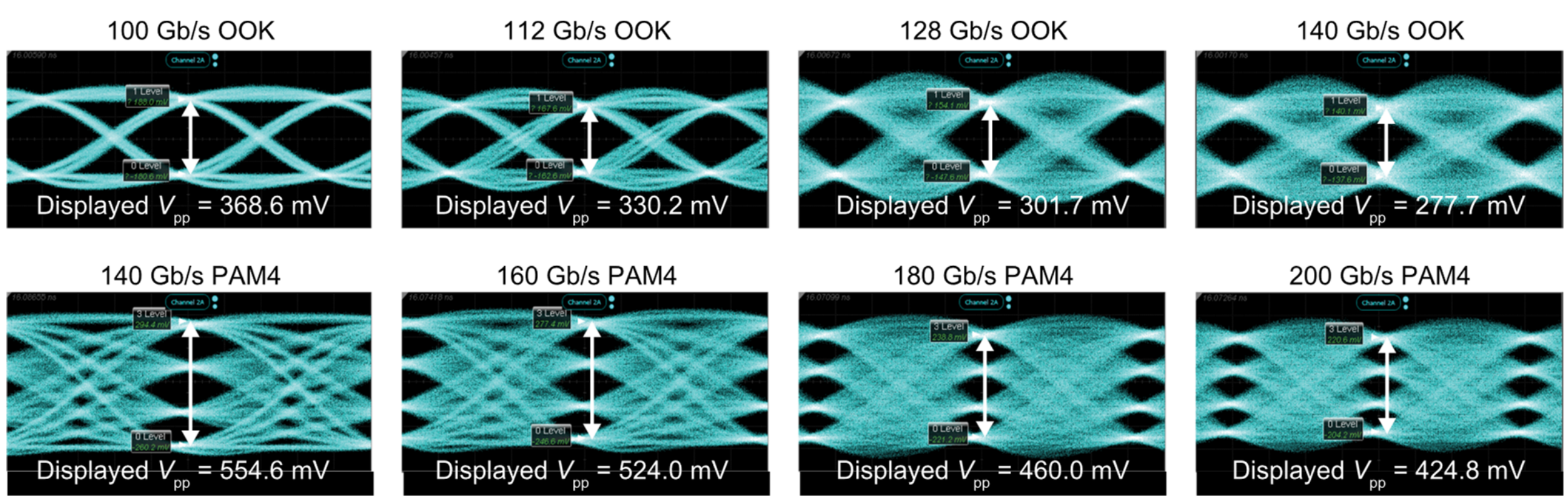

Supplementary Figure 9. Measured eye diagrams of the electrical input signals when the AWG output amplitude was set to V_{pp} = 1 V as the setting value. The top row shows the OOK signals for data rates ranging from 100 Gb/s to 140 Gb/s and the bottom row presents the PAM4 signals for data rates from 140 Gb/s to 200 Gb/s. Clear eye openings are maintained across all measured bit rates.

N1046A). The 20-dB attenuator was inserted before the remote sampling head to prevent damage to the receiver input.

Supplementary Figure 9 shows the eye diagrams of the electrical input waveforms measured using this setup. During the measurement, the DCA channel was configured with an internal 9-dB attenuator

compensation setting (InstrumentSetup.setx, see Supplementary Data 1). Therefore, the voltage displayed on the DCA already included a compensation factor of 2.818. To obtain the peak-to-peak drive voltage at the bias-T output, the displayed voltage was further multiplied by the remaining compensation factor for the external attenuator, corresponding to 11 dB or a factor of 3.55. For example, when the DCA displayed a peak-to-peak voltage of 368.6 mV, applying this compensation factor yielded a drive voltage of V_{pp} = 1.3 V at the bias-T output. For the AWG, an in-system calibration was applied to compensate for the high-frequency roll-off caused by the high-frequency cables, the driver amplifier, and the bias-T. Additionally, a raised-cosine roll-off filter was used to suppress alias signals. No digital pre-emphasis was applied in the AWG. Under these conditions, clear eye openings were observed for OOK and PAM4 signals at data rates up to 200 Gb/s.

Supplementary Note 5 – Power Consumption and Bit Energy Efficiency

The driving power consumption P and the bit energy E_{bit} were calculated for a random data sequence. For continuous signal transitions, the signal voltage $v(t)$ can be expressed as a superposition of the voltage level of each symbol a_k and the elementary pulse shape $g(t)$:

$$v(t) = \sum_{k=-\infty}^{\infty} a_k g(t - kT)\,, \tag{2}$$

where T represents the symbol period. The average power P was obtained from the product of the mean square value of the symbol levels $E[a_k^2]$ and the energy integral of the pulse shape as

$$P = \frac{E[a_k^2]}{R_\mathrm{T}} \cdot \frac{1}{T} \int_{-\infty}^{\infty} g(t)^2 dt\,. \tag{3}$$

Assuming an ideal Nyquist pulse or a root-raised-cosine waveform, the integral term is always unity. Therefore, the average power is simplified to $P = E[a_k^2] / R_T$. For OOK, assuming a single-ended peak-to-peak voltage of V_{pp}, the voltage levels are $\pm V_{pp}/2$, yielding a mean square value of $E[a_k^2] = V_{pp}^2/4$. Because the differential drive consumes power across two signal lines, the total average power for OOK is given by

$$P_\mathrm{OOK} = \frac{V_\mathrm{pp}^2}{2R_\mathrm{T}}\,. \tag{4}$$

For PAM4, the four voltage levels are $\pm V_{pp}/2$ and $\pm V_{pp}/6$, resulting in a mean square value of $E[a_k^2] = 5V_{pp}^2/36$, and the total average power for PAM4 is given by

$$P_\mathrm{PAM4} = \frac{5V_\mathrm{pp}^2}{18R_\mathrm{T}}\,. \tag{5}$$

The bit energy was obtained by dividing the average power by the bit rate.

Supplementary References

[1]. Kawahara, K. et al. High-efficiency compact optical transmitter with a total bit energy of 0.78 pJ/bit including silicon slow-light modulator and open-collector current-mode driver. *IEEE J. Sel. Top. Quantum Electron.* **32**, 3400111 (2025).

[2]. Terada, Y., Tatebe, T., Hinakura, Y. & Baba, T. Si Photonic Crystal Slow-Light Modulators with Periodic p–n Junctions. *J. Lightwave Technol.* **35**, 1684–1692 (2017).

[3]. Kawahara, K. et al. High-speed, low-voltage, low-bit-energy silicon photonic crystal slow-light modulator with impedance-engineered distributed electrodes. *Optica* **11**, 1212–1219 (2024).

[4]. Hirotani, K., Shiratori, R. & Baba, T. Si photonic crystal slow-light waveguides optimized through informatics technology. *Opt. Lett.* **46**, 4422 (2021).

[5]. Nguyen, H. C. et al. Sub-100 μm Photonic Crystal Si Optical Modulators: Spectral, Athermal, and High-Speed Performance. *IEEE J. Sel. Top. Quantum Electron.* **19**, 127–137 (2013).
