# Supplementary material for: Reduced-DSP 200G/λ low-voltage slow-light silicon modulator enabled by built-in EO equalizer
Source: Nat Commun. 2026 Oct 22;17:10446. doi: 10.1038/s41467-026-78128-1 (PMC13690699; doi:10.1038/s41467-026-78128-1)
Supplement: Supplementary file 2 [file 41467_2026_78128_MOESM4_ESM.pdf]

nature portfolio

Peer Review File

Reduced-DSP 200G/λ low-voltage slow-light silicon modulator enabled by built-in EO equalizer

Corresponding Author: Dr Keisuke Kawahara

This file contains all reviewer reports in order by version, followed by all author rebuttals in order by version.

Version 0:

Reviewer comments:

Reviewer #1

(Remarks to the Author)
This manuscript presents a silicon slow-light modulator integrated with an on-chip electro-optic equalizer, aiming to overcome the fundamental bandwidth-efficiency trade-off. By combining photonic crystal waveguides with delay lines, the authors report an extended 80 GHz bandwidth and data rates of 140 Gb/s OOK and 200 Gb/s PAM4, claiming low-voltage, light-DSP operation within a compact footprint.
However, despite promising figures of merit, the manuscript exhibits critical deficiencies in methodological consistency and data completeness. The presented experimental evidence is insufficient to robustly validate the central claims. These fundamental issues must be addressed before publication:
1. To substantiate the proposed advantages, a direct comparison with state-of-the-art silicon modulators is essential. The authors must include a comprehensive comparison table in the main text, listing key metrics and specific test conditions. Moreover, the authors should define a composite Figure of Merit （like bandwidth × efficiency） to quantify the overall system advantage, justifying the design trade-offs regarding individual parameters.
2. Several key parameters are absent or lack sufficient explanation: 1) While OOK modulation driving metrics are provided, the drive voltage, bias voltage, and power consumption for the PAM4 modulation are omitted. 2) The Methods section refers to "optimized electrodes" and "optical delay lines" but lacks detailed geometric dimensions. The authors must specify the electrode and waveguide dimensions corresponding to the EO equalization time constants (TE, TS, TD). Furthermore, the authors should clarify how these constants are engineered and discuss their sensitivity to fabrication tolerances. 3) The theoretical model or fitting procedure used for the 80 GHz bandwidth extrapolation must be provided. Additionally, please detail the derivation methods for modulation efficiency and power consumption.
3. Certain descriptions in the manuscript are ambiguous and require clarification. First, the authors employ “AWG in-system calibration”, “receiver equalization”, and “16×/32× averaging”; it should be clarified whether these are considered part of the DSP. Second, the claim of CMOS compatibility is questionable; the OOK modulation requires a drive voltage of 2 Vpp and still relies on an external amplifier during testing. The statement “eliminates reliance on power-dissipating DSPs and drivers” is unsubstantiated. Furthermore, the use of different devices for bandwidth (N=3) and modulation (N=4) experiments undermines the validity of the results. The authors should provide data from a unified device configuration to ensure consistency.
4. In the Discussion section, the authors assert that the proposed modulator is suitable for WDM applications; however, this claim is undermined by critical performance bottlenecks. Specifically, the usable slow-light bandwidth of the proposed modulator is severely limited (~6 nm), and the on-chip insertion loss is prohibitive (> 10 dB). Furthermore, the device exhibits significant performance non-uniformity across the spectrum; notably, the modulation efficiency varies by a factor of more than three within the bandwidth. Additionally, the inherent sensitivity of photonic crystal structures to fabrication tolerances poses a scalability challenge. A rigorous and comprehensive analysis reconciling these limitations with the claimed WDM compatibility is mandatory.
5. How does N affect the frequency response of the modulator without an EO equalizer in Fig. S2, since the phase shifter length remains constant?

Reviewer #2

(Remarks to the Author)
Key results
The authors report a slow-light silicon photonic crystal waveguide (PCW) Mach–Zehnder modulator with a built-in

electro-optic feed-forward equalizer extend the 3-dB bandwidth by a >2x factor. The buit-in electro-optic equalizer is the primary innovation reported. The device is presented as a compact, low-voltage silicon modulator architecture that improves EO bandwidth while reducing dependence on electronic equalization. The manuscript reports an EO 3-dB bandwidth increase from ~35 GHz to ~80 GHz for the demonstrated condition, DSP-free eye openings up to 180 Gb/s PAM4, and 200 Gb/s PAM4 operation with a low-complexity 11-tap receiver FFE.

Validity

The device concept is interesting and the measurements are promising. However, the current evidence is not yet sufficient to substantiate deployment-level DSP-independent performance for practical CPO based links, even though the manuscript reports DSP-independent high-speed operation demonstrations.

A central issue is that the manuscript primarily uses eye diagrams and TDEC/TDECQ-style metrics to support DSP-free or DSP-light feasibility, but it does not provide BER vs OMA (or BER vs received power) data. For a 200 Gb/s/λ Intensity Modulation (IM) Direct Detection (DD) transmitter claim, BER-based evidence is important to connect the demonstrated eye quality to FEC-relevant operating margins and realistic link budgets.

In addition, the reported eye behavior of the built-in equalizer appears to show residual pattern dependence/timing distortion. This may indicate remaining impairments not fully addressed by bandwidth improvement alone or potentially caused by it (for example, phase/group-delay effects have not been discussed). It would be helpful if the authors either quantified this or clarified the expected limitation of the built-in equalizer, including frequency agility.

The manuscript also frames TDEC/TDECQ results using a Soft Detection (SD) FEC threshold of BER = 2×10^{-2}. Because the target application is IM/DD OOK/PAM4 transmission, the authors should justify the relevance of this threshold to the intended use case and/or provide comparison to a direct-detect relevant baseline such as Ethernet 802.3.

Finally, while the voltage swings are described as advanced CMOS technology compatible, the experimental setup relies on a 256-GS/s AWG and 70-GHz high-voltage broadband amplifiers. This does not invalidate the device result, but it does leave open an important practical question: what bandwidth/voltage/reliability constraints would apply in a realizable CMOS driver implementation at the claimed rates?

Significance

Reducing DSP and driver burden at 200 Gb/s/λ is an important problem, and integrating an EO equalizer into the modulator is a potentially valuable device-level approach. The manuscript therefore has potential significance.

However, the practical significance is difficult to assess fully without:

• BER vs OMA (or BER vs received power),
• device-level insertion loss (all frequency plots are normalized to 0dB),
• and a clearer application-appropriate FEC framing.

These additions would allow readers to better judge whether the work supports deployment-level DSP-free performance, or more conservatively, a “DSP-light” transmitter and/or receiver architecture.

Data and methodology

The experiments use a Keysight M8199B AWG (256 GS/s), 70 GHz broadband amplifiers, and a high speed optical receiver; eyes and TDEC/TDECQ are reported for OOK and PAM4. This methodology is reasonable for device level characterization, however, the absence of BER vs OMA and device insertion loss prevents translation of the measurements into deployable link budgets.

Analytical approach

The manuscript provides a convincing device concept and amplitude-response-oriented EO characterization. To strengthen the analytical case for DSP-free (or DSP-reduced) operation, I suggest the authors connect the measured EO response more directly to system-level impairments. In particular, if phase/group-delay effects are expected to contribute to pattern-dependent timing distortion, this should be discussed and, if possible, characterized. Even a concise discussion would help readers understand what the integrated EO equalizer corrects effectively and what residual impairments may remain or enhanced.

Suggested improvements

1. Add BER vs OMA (or BER vs received power) at the demonstrated OOK/PAM4 operating points, especially for the headline high-rate cases. This is the most important missing evidence for evaluating practical performance and FEC margin.
2. Clarify the “DSP-free” / “DSP-independent” claim and align the claim language with the evidence.
3. Justify the FEC baseline used for TDEC/TDECQ interpretation (SD-FEC BER = 2×10^{-2}), or provide an additional comparison using a direct-detection relevant baseline for the intended application.
4. Report insertion loss. This is necessary to assess the practical value of the demonstrated EO bandwidth enhancement.
5. Discuss driver feasibility more explicitly in the context of the AWG + 70-GHz amplifier-based setup. A short discussion of expected driver constraints (swing, bandwidth, reliability/device stress) would improve realism without requiring a full electronic co-design demonstration.
6. Address temperature robustness / calibration sensitivity of the built-in optical equalizer. Because the manuscript is motivated by practical deployment, it would help to clarify how temperature drift affects equalizer timing/response and whether any compensation is required during operation. This may require additional DSP to allow for dynamic calibration to compensate for temperature drift.
7. Improve CPO context with quantitative comparisons. A comparison on bandwidth density, total link power (with clear accounting), and PAM4 linearity/DSP requirements relative to representative alternatives (such as Silicon Photonics ring-based resonators) would help readers evaluate system-level impact.

Clarity and context

The manuscript is generally clear and technically interesting. However, the central messaging would benefit from a sharper distinction between what is directly demonstrated (EO bandwidth enhancement, high-speed eyes, TDEC/TDECQ results, 200 Gb/s PAM4 with low-complexity Rx FFE), and what is inferred/projected for practical links (deployment-level DSP-free

performance, link-budget sufficiency, CPO-level system impact). I recommend revising the wording accordingly unless additional BER and insertion-loss evidence is added.

References
The manuscript cites relevant prior work on slow-light silicon modulators and high-speed silicon photonic links. If the authors continue to emphasize CPO significance, a more explicit quantitative comparison to representative CPO-oriented modulator approaches would improve context and help clarify the manuscript's distinct contribution.

Version 1:

Reviewer comments:

Reviewer #1

(Remarks to the Author)
I would like to thank the authors for their thorough and constructive responses to all the comments from the first round of review. The manuscript has been significantly improved. I have only three minor suggestions for the authors before publication. First, given the extensive amount of experimental data, the authors should carefully double-check the consistency between the text and figures throughout the paper. For instance, in lines 115–116, the text states that the 100G and 112G eye diagrams in Fig. 4a remain clear down to 0.7 V, but the figure actually shows the 100G eye diagram at 0.8 V. Second, I suggest that the authors consider replacing the N=3 data in Fig. 3d with the corresponding N=4 data. I understand that using the N=3 device highlights the bandwidth enhancement from the EO equalizer more clearly, but the subsequent DSP-independent experiments are still demonstrated using the N=4 device. Since readers will naturally be more interested in the actual performance (such as bandwidth and modulation efficiency) of the N=4 device, presenting the N=4 data in Fig. 3d would allow for a more direct and coherent comparison. Lastly, please briefly clarify how the driving voltage was adjusted in the experimental setup (e.g., via the AWG output amplitude or by tuning the electrical attenuators). I believe the manuscript will be ready for publication once these minor details are addressed.

Reviewer #2

(Remarks to the Author)
I thank the authors for a thorough revision. The disclosure of the drive-voltage error, the added comparison table, insertion-loss values, HD-FEC re-analysis, group-delay discussion, and the sharper “DSP-free” vs. “DSP-independent” distinction all strengthen the manuscript.

One point does not fit the corrected drive voltages. The 140-Gb/s PAM4 drive voltage changed 5.2→2.0 V (ratio 0.385), whereas a uniform correction predicts ≈1.85 V. This discrepancy is beyond rounding and should be explained or corrected.

The core claim that a built-in EO equalizer improves transmitter waveform quality on a slow-light Si modulator is correctly and sufficiently supported by the with/without-equalizer TDEC/TDECQ comparison under identical conditions. I accept TDEC/TDECQ as the appropriate metric for that claim and do not require BER for it. However, the manuscript also carries a deployment-level framing (title “DSP-independent 200G/λ,” CPO discussion, multi-Tb/s capacity projections) that the present evidence does not establish. Please choose one of the following:
- Scope the claims to transmitter characterization, retaining TDEC/TDECQ and removing or clearly qualifying the link-level and CPO assertions. No new measurements needed, or
- retain the deployment framing, in which case at least one BER-vs-received-power curve is required. This is needed because TDECQ is anchored to assumed receiver and target-BER conditions, the eyes use averaging that suppresses real receiver noise, and the 200-Gb/s result combines the transmitter with a receiver FFE.

The “DSP-free” vs. “DSP-independent” clarification is part of this same issue. The new definitions are reasonable. The problem is that the title still advertises “DSP-independent 200G/λ,” yet the only 200G/λ result with acceptable quality uses receiver DSP; an 11-tap symbol-spaced FFE is DSP. The strong term thus covers precisely the rate at which the DSP-free claim fails. Please remove “DSP-independent” from the 200G/λ framing (e.g., “DSP-light” or “reduced-DSP” would be accurate).

The reported 80 GHz bandwidth is obtained from the N = 3 device by linear-in-dB extrapolation beyond the 67 GHz measurement limit, while all modulation data come from the N = 4 device, which shows a measured bandwidth of 62 GHz. The two-device rationale is reasonable, but the abstract highlights the 35 to 80 GHz improvement from a device with no modulation data. Since the revision now includes the N = 4 response, the modulation discussion should highlight the 62 GHz result and clearly identify which device each value refers to.

The measured on-chip loss (up to 15.5 dB for N = 4) requires a downstream EDFA in the present setup, which is in tension with the CPO claims. A brief, explicit link-budget statement or at least an acknowledgment that CPO viability is contingent on reducing this loss would close the gap.

Version 2:

Reviewer comments:

Reviewer #1

(Remarks to the Author)
All previous comments have been addressed. The paper is ready for publication. I recommend acceptance in its current form.

Reviewer #2

(Remarks to the Author)
I thank the authors for their thorough revisions and for carefully addressing the reviewers' comments and suggestions. I am satisfied with the responses provided and believe the manuscript has been significantly improved.

Open Access This Peer Review File is licensed under a Creative Commons Attribution 4.0 International License, which permits use, sharing, adaptation, distribution and reproduction in any medium or format, as long as you give appropriate credit to the original author(s) and the source, provide a link to the Creative Commons license, and indicate if changes were made.
In cases where reviewers are anonymous, credit should be given to 'Anonymous Referee' and the source.
The images or other third party material in this Peer Review File are included in the article's Creative Commons license, unless indicated otherwise in a credit line to the material. If material is not included in the article's Creative Commons license and your intended use is not permitted by statutory regulation or exceeds the permitted use, you will need to obtain permission directly from the copyright holder.
To view a copy of this license, visit https://creativecommons.org/licenses/by/4.0/

Response to the reviewers' comments

The reviewers' comments are enclosed in boxes. The authors' responses are written in blue. The modified parts in the manuscript are written in red, and the retained parts are written in black. The references in this document are listed at the end and do not correspond to the reference numbers in the original manuscript.

Reviewers' comments

Authors' responses

Manuscript modified parts

Manuscript retained parts

General Response:

We would like to express our sincere gratitude to the editors and reviewers for their constructive feedback and careful evaluation of our manuscript. Before addressing the specific comments, we report an important correction resulting from a re-examination of our data. During the preparation of this revised manuscript, we discovered an error in the calculated values of the drive voltage in the original manuscript. This error occurred because compensation for the external attenuator was applied twice during the measurement of the drive electrical signal waveform. Due to this calculation error, we reported values in the original manuscript that overestimated the actual voltage applied to the device. Consequently, we underestimated the performance of our device. In this revised manuscript, we have entirely replaced all corresponding figures and numerical values with data based on the correct conversion. The revised data confirms operation at a lower drive voltage than the values reported in the original manuscript.

In the following subsection, we detail the exact cause of this calculation error. Following that, we specify the exact locations of the revisions within the manuscript and explain the newly added Supplementary Data. Our point-by-point responses to the reviewers' comments are provided after this general response.

Correction of the calculated drive voltage

Figure R1 shows the measurement setup of the electrical drive signal waveform. In this setup, the output signal from the AWG (Keysight M8199B) passed through the driver amplifier (SHF M827B) and bias-T (SHF BT110R), and was then connected to a 20-dB attenuator (Anritsu 41V-20) through a 5-cm cable. The signal was then input to Channel 2A of the DCA through a remote sampling head (Keysight N1046A).

During the initial data processing, we manually multiplied the peak-to-peak voltage by a factor of 10 to compensate for the 20-dB attenuation. However, a subsequent re-examination revealed that an internal 9-dB compensation setting (a factor of 2.818) from a previous measurement remained active on the DCA. This configuration is recorded in the DCA settings file (InstrumentSetup.setx, See Supplementary Data 1) saved during the measurement as follows.

```
<Setting Name="CAttenuatorStateSetting.Channel2A">On</Setting>
<Setting Name="CAttenuatorRatioSetting.Channel2A">2.818</Setting>
```

For example, the DCA displayed a peak-to-peak voltage of 368.6 mV in the 100-Gb/s OOK measurement, as shown in Fig. R2. Originally, we manually applied the 20-dB compensation to this displayed value, resulting in a reported drive voltage of V_{pp} = 3.7 V. However, because the

displayed 368.6 mV already included the internal 9 dB compensation, the correct manual compensation is 11 dB, corresponding to a factor of 3.55. Applying this correct factor yields an actual drive voltage of V_{pp} = 1.3 V. This duplication in the compensation process resulted in the overestimation of the drive voltages reported in the original manuscript, and consequently, an underestimation of the device performance.

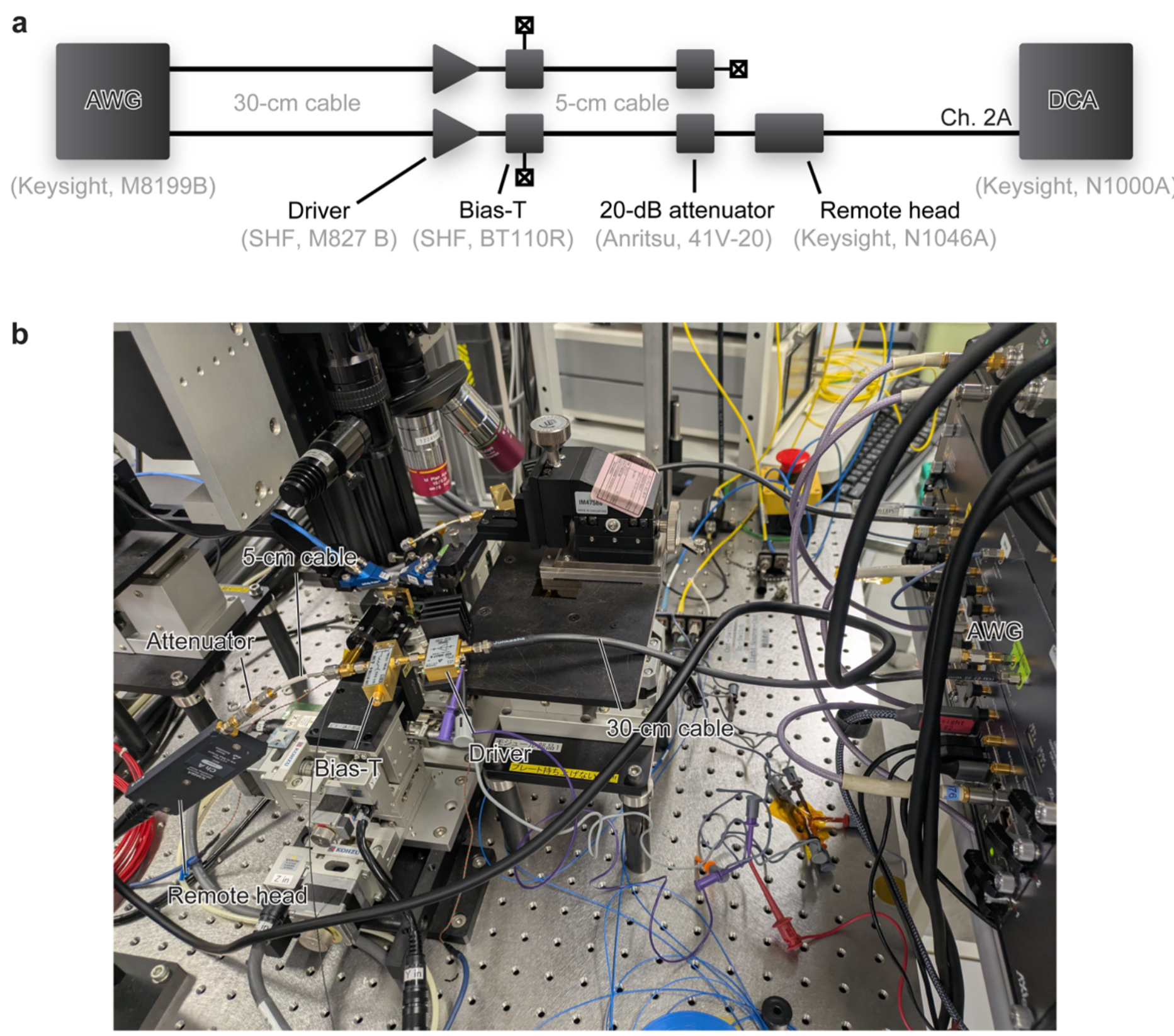

Fig. R1 Measurement setup for the electrical driving signal waveforms. (a) Block diagram. (b) Photograph.

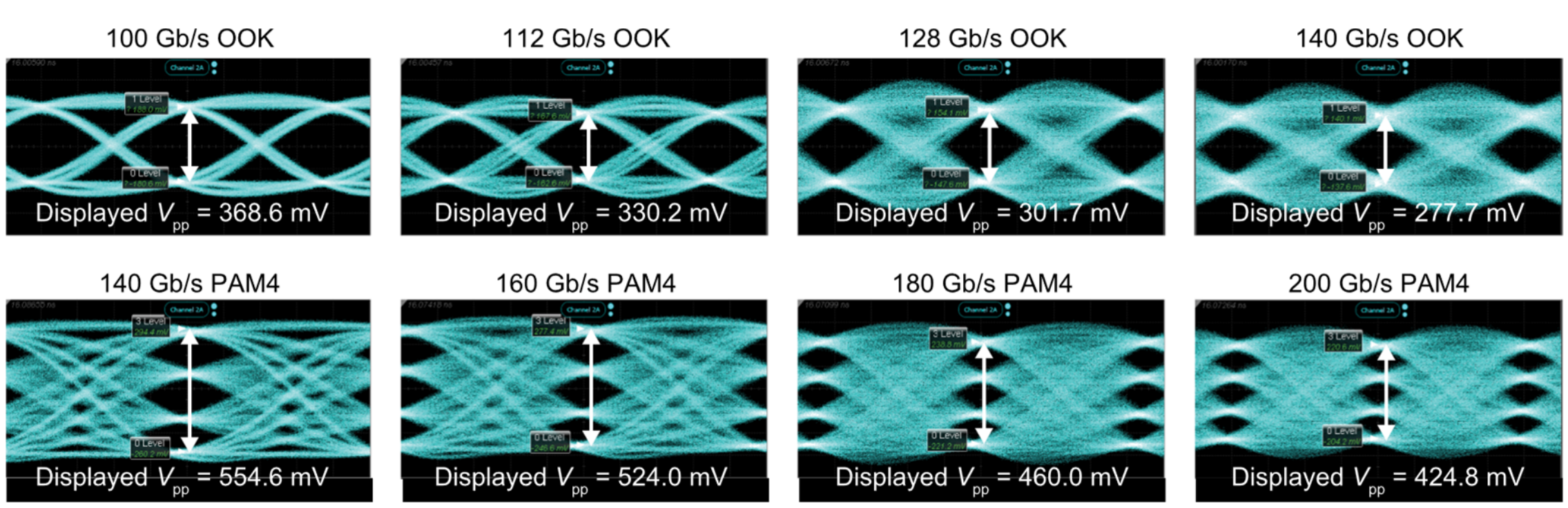

Fig. R2 Measured eye diagrams of the electrical input signals when the AWG output amplitude was set to V_{pp} = 1 V as the setting value. The top row shows the OOK signals for data rates ranging from 100 Gb/s to 140 Gb/s and the bottom row presents the PAM4 signals for data rates from 140 Gb/s to 200 Gb/s. Clear eye openings are maintained across all measured bit rates.

Accordingly, we have made the following revisions to the manuscript.

Figure 4: We have corrected the drive voltages.

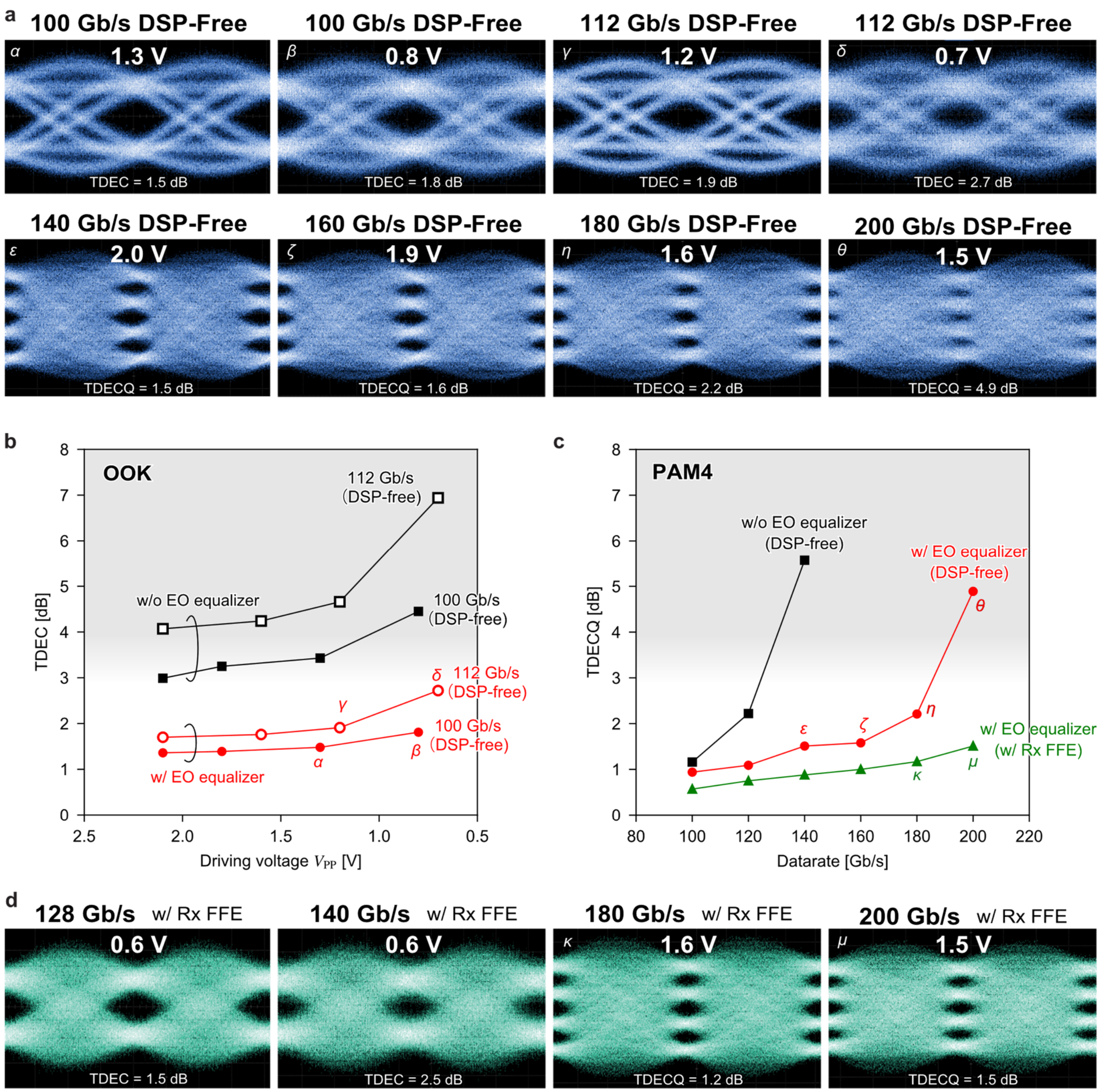

Fig. 4 Measured optical modulation characteristics. (a) DSP-free eye diagrams for OOK (100–112 Gb/s) at V_{pp} = 1.3–0.7 V, V_{DC} = −2.5 V and PAM4 (140–200 Gb/s) at V_{pp} = 2.0–1.5 V, V_{DC} = −2.5 V. (b) Measured TDEC versus drive voltage for OOK signals. (c) TDECQ versus data rate for PAM4 signals up to 200 Gb/s. (d) Eye diagrams for 128–200-Gb/s OOK and PAM4 signals enabled by the EO equalizer and the low-complexity 11-tap Rx FFE.

Main text lines 115–120: As shown in the upper panel of Fig. 4a, clear eye openings at 100 Gb/s and 112 Gb/s were maintained down to V_{pp} = 0.7 V. Without the EO equalizer, the modulator's

intrinsic bandwidth limitation caused TDEC values exceeding 3 dB across the entire voltage range, with severe degradation around $V_{pp} \approx 0.7$ V. In contrast, applying the EO equalizer suppressed the increase in TDEC in the whole voltage range. Even under the lowest-voltage condition, the TDEC stayed as low as 1.8 dB at 100 Gb/s with $V_{pp} = 0.8$ V and 2.7 dB at 112 Gb/s with $V_{pp} = 0.7$ V.

Main text lines 129–130: As shown in Fig. 4d, the achievable OOK data rate reaches 140 Gb/s at a low drive voltage of 0.6 V.

Supplementary information lines 80–91: Figure S8 shows the measurement setup of the electrical drive signal waveform. In this setup, the output signal from the AWG (Keysight M8199B) passed through the driver amplifier (SHF M827B) and bias-T (SHF BT110R), and was then connected to a 20-dB attenuator (Anritsu 41V-20) through a 5-cm cable. The attenuated signal was then input to Channel 2A of the DCA through a remote sampling head (Keysight N1046A). The 20-dB attenuator was inserted before the remote sampling head to prevent damage to the receiver input.

Figure S9 shows the eye diagrams of the electrical input waveforms measured using this setup. During the measurement, the DCA channel was configured with an internal 9-dB attenuator compensation setting (InstrumentSetup.setx, See Supplementary Data 1). Therefore, the voltage displayed on the DCA already included a compensation factor of 2.818. To obtain the peak-to-peak drive voltage at the bias-T output, the displayed voltage was further multiplied by the remaining compensation factor for the external attenuator, corresponding to 11 dB or a factor of 3.55. For example, when the DCA displayed a peak-to-peak voltage of 368.6 mV, applying this compensation factor yielded a drive voltage of $V_{pp} = 1.3$ V at the bias-T output.

Supplementary data 1: The DCA measurement dataset of the electrical signal waveforms corresponding to Fig. R2, including the InstrumentSetup.setx file, has been added.

Response to Reviewer #1:

This manuscript presents a silicon slow-light modulator integrated with an on-chip electro-optic equalizer, aiming to overcome the fundamental bandwidth-efficiency trade-off. By combining photonic crystal waveguides with delay lines, the authors report an extended 80 GHz bandwidth and data rates of 140 Gb/s OOK and 200 Gb/s PAM4, claiming low-voltage, light-DSP operation within a compact footprint.

However, despite promising figures of merit, the manuscript exhibits critical deficiencies in methodological consistency and data completeness. The presented experimental evidence is insufficient to robustly validate the central claims. These fundamental issues must be addressed before publication:

We appreciate the reviewer's valuable comments on our manuscript. We have carefully addressed all points raised by the reviewer and have revised the manuscript accordingly. Our detailed responses to the points are provided below.

1. To substantiate the proposed advantages, a direct comparison with state-of-the-art silicon modulators is essential. The authors must include a comprehensive comparison table in the main text, listing key metrics and specific test conditions. Moreover, the authors should define a composite Figure of Merit (like bandwidth × efficiency) to quantify the overall system advantage, justifying the design trade-offs regarding individual parameters.

We agree that a direct comparison with other Si modulators is essential. We have added Table 1 to the main text to compare the present device with representative Si modulators. The table comprehensively summarizes the performance metrics, including the data rate with and without DSP, drive voltage, driving bit energy, EO bandwidth, phase-shifter length, optical passband, modulation efficiency, and insertion loss. This comparison shows that our PCW modulator achieves the highest DSP-free data rate among the listed devices and reaches 200-Gb/s PAM4 operation with only an 11-tap receiver FFE. At the same time, it achieves the smallest $V_\pi L$ and the lowest drive voltage, while retaining the non-resonant behavior of MZMs.

We also carefully considered the reviewer's suggestion to define a composite FoM, such as a bandwidth–efficiency metric. In fact, a metric based on EO bandwidth divided by $V_\pi L$ would favor our device among those listed in Table 1. Nevertheless, we did not introduce a new FoM because such a comparison would depend strongly on how the FoM is defined, rather than on the full set of

experimentally relevant conditions. Therefore, we explicitly present all relevant metrics and test conditions in Table 1 so that performance can be evaluated directly. We believe that this form of comparison provides a more transparent and balanced assessment of the advantages and limitations of the proposed modulator than a newly defined composite FoM. The specific revisions made to the manuscript are as follows.

Main text lines 136–152: It has been recognized for years that Si photonics is a primary candidate for multi-Tb/s interconnects, yet the trade-off between modulation efficiency and operating bandwidth remains a fundamental bottleneck. As shown in Table 1, recent modulators have exceeded 200 Gb/s, although this progress has depended on high drive voltages[35,45,46], long phase shifters[47,48], and extensive DSP[14,17,35,45-50]. MRMs provide compact footprints and low bit energy, whereas their resonant operation requires wavelength control[17,49,50]. In this article, we have shown that integrating a built-in EO equalizer with a Si PCW slow-light modulator is essential to overcome this trade-off without relying on DSP. The extraordinary modulation efficiency of the slow-light effect is critical for device miniaturization, which reduces the active length to the deep sub-millimeter scale. This short interaction length is vital for suppressing RF propagation loss, thereby allowing the integrated equalizer to maintain an ideal equalizer response for multi-level modulation. For this reason, we designed a segmented phase shifter with an integrated FIR filter and confirmed that the EO bandwidth increases from 35 GHz to 80 GHz. At the same time, the present modulator achieves the smallest $V_{\pi}L$ and the lowest drive voltage among those listed in Table 1, while retaining the non-resonant behavior. Following these designs, we have successfully demonstrated the first DSP-free 180-Gb/s PAM4 operation using a Si slow-light modulator. We have also confirmed that the device achieves an extremely low energy consumption of 26 fJ/bit with a CMOS-compatible drive voltage and has achieved 200 Gb/s PAM4 using only low-complexity electronic assistance.

<u>Table 1:</u>

Table 1. Comparison with representative silicon modulators.

Ref.	Type	Phase shifter	DSP-free data rate [Gb/s]	Data rate w/ DSP [Gb/s]	V_{pp} [V]	Driving bit energy† [fJ/bit]	EO f_{3dB} [GHz]	Phase shifter length [mm]	Optical passband [nm]	$V_\pi L$ [V•cm]	Insertion loss [dB]
**This work**	**MZM**	**PCW**	**180 (PAM4)<br>112 (OOK)**	**200 (PAM4), 140 (OOK)<br>11-tap FFE**	**1.5 (PAM4)<br>0.6 (OOK)**	**63 (PAM4)<br>26 (OOK)**	**≈ 80 (N = 3)<br>62 (N = 4)**	**0.25‡ (N = 3)<br>0.32‡ (N = 4)**	**6**	**0.3**	**11.7 (N = 3)<br>15.5 (N = 4)**
[35]	MZM	Bragg-grating	N.R.	400 (PAM4)<br>bi-GRU/T-biGRU ANN equalizer	5	347	90	0.249	7	0.82	10.5
[14]	MZM	Rib	N.R.	350 (PAM4)<br>99FF/99FB-tap DFE	2.7	116	42	1.5	N.R.	1.7	3.6
[38]	MZM	Rib	140 (OOK)	N.R.	5	893	>110	0.9	N.R.	1.62	4.3
[45]	MZM	Rib	N.R.	100 (PAM4), 138 (PAM8)<br>Pre-distortion + VNLE/NWF/BCJR	3.2	569 (PAM4)<br>318 (PAM8)	>35	2	N.R.	0.82	9.6
[46]	MZM	Rib	N.R.	106 (PAM4)<br>Pre-emphasis + 21-tap FIR	4.5	531	7.7	1.6	N.R.	1.02	2.1
[47]	MZM	Rib	N.R.	Net 225 (PAM8)<br>2nd-order VNLE	2.3	72	47	2.5	N.R.	1.35	5.4
[48]	MZM	Rib	40 (PAM4)	46 (PAM4)<br>FFE/NLE/MLSE	1.1, 2.1	611	27	3	N.R.	0.45	N.R.
[49]	MRM	Rib	N.R.	200 (PAM4)<br>21-tap FFE	1.6	6.3<br>(capacitive)	48.6	N.R.	≈ 0.35	0.6	6
[17]	MRM	Rib	N.R.	330 (PAM8), 280 (PAM-4)<br>Pre-distortion/pre-emphasis + PNLE	1.8	3.1<br>(capacitive)	>50<br>(detuned)	N.R.	≈ 0.3	0.63	N.R.
[50]	MRM	Rib	N.R.	170 (PAM4)<br>Pre-emphasis + 33FF/9FB-tap DFE	2.4	N.R.	47	N.R.	≈ 0.51	N.R.	N.R.

† For MZM-based devices, driving bit energy was calculated from the drive voltage and R_T using the method described in Supplementary Information 5. R_T = 50 Ω was assumed when R_T was not reported.

‡ Including the built-in EO equalizer.

2. 1) Several key parameters are absent or lack sufficient explanation: 1) While OOK modulation driving metrics are provided, the drive voltage, bias voltage, and power consumption for the PAM4 modulation are omitted.

We have added the bias voltage and drive voltage for the PAM4 modulation to the main text and Fig. 4 as follows.

Main text lines 223–224: The p–n junction of the modulator was biased at –2.5 V for both OOK and PAM4 modulation.

Figure 4: We have added drive voltages and bias voltage as follows:

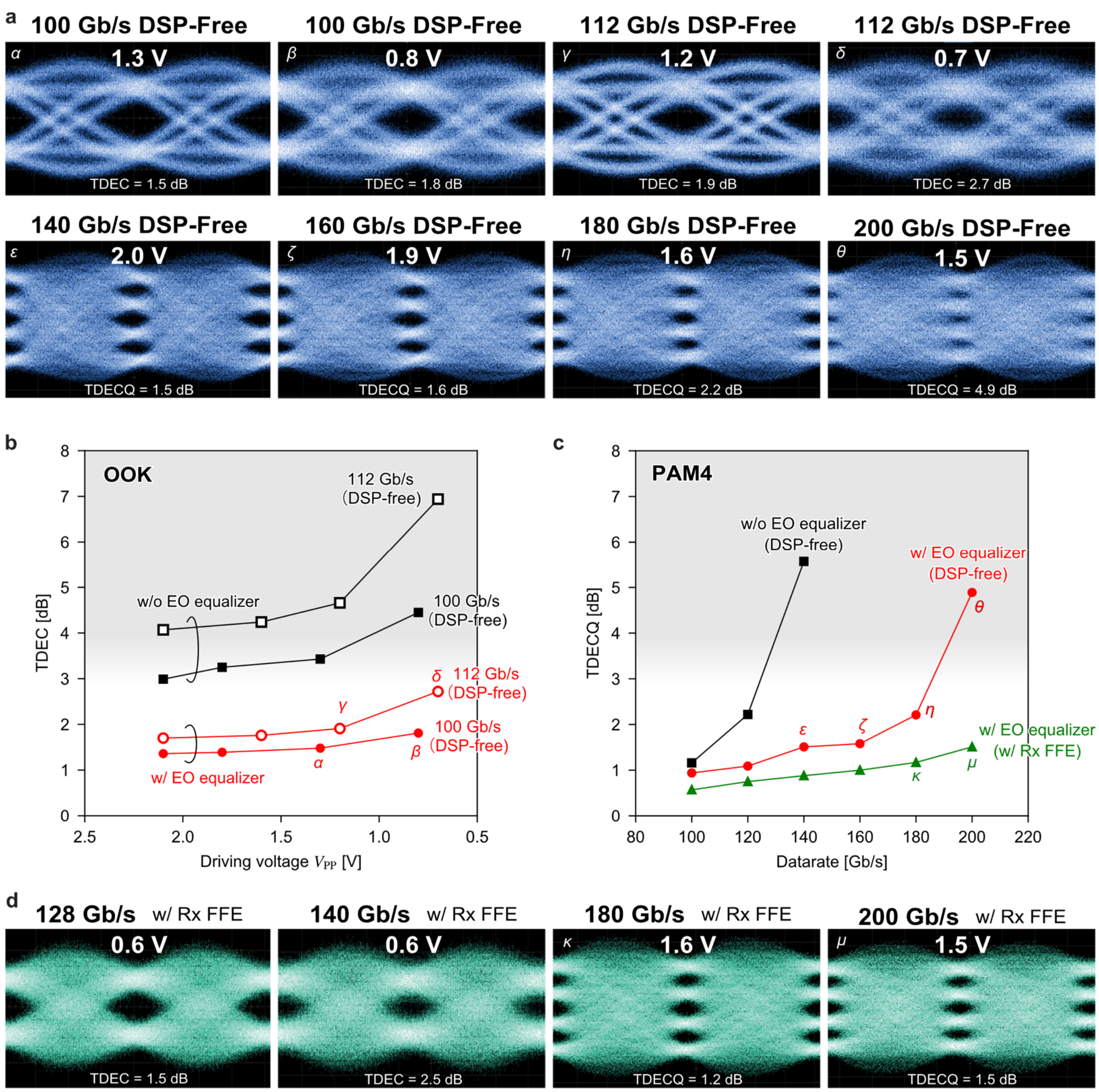

Fig. 4 Measured optical modulation characteristics. (a) DSP-free eye diagrams for OOK (100–112 Gb/s) at V_{pp} = 1.3–0.7 V, V_{DC} = −2.5 V and PAM4 (140–200 Gb/s) at V_{pp} = 2.0–1.5 V, V_{DC} = −2.5 V. (b) Measured TDEC versus drive voltage for OOK signals. (c) TDECQ versus data rate for PAM4 signals up to 200 Gb/s. (d) Eye diagrams for 128–200-Gb/s OOK and PAM4 signals enabled by the EO equalizer and the low-complexity 11-tap Rx FFE

Regarding the power consumption, we have revised the calculation method to evaluate the average power for random data sequences more rigorously. By assuming an ideal Nyquist pulse or a root-raised-cosine waveform, the average power is derived directly from the mean square value of the symbol levels. This approach properly accounts for continuous signal transitions and provides a valid power estimation for both OOK and PAM4. We have added the detailed explanation to the Supplementary Information 5.

Supplementary information lines 97–112: The driving power consumption P and the bit energy E_{bit} were calculated for a random data sequence. For continuous signal transitions, the signal voltage $v(t)$ can be expressed as a superposition of the voltage level of each symbol a_k and the elementary pulse shape $g(t)$:

$$v(t) = \sum_{k=-\infty}^{\infty} a_k g(t - kT)\,, \tag{3}$$

where T represents the symbol period. The average power P was obtained from the product of the mean square value of the symbol levels $E[a_k^2]$ and the energy integral of the pulse shape as

$$P = \frac{E[a_k^2]}{R_T} \cdot \frac{1}{T} \int_{-\infty}^{\infty} g(t)^2 dt \tag{4}$$

Assuming an ideal Nyquist pulse or a root-raised-cosine waveform, the integral term is always unity. Therefore, the average power is simplified to $P = E[a_k^2] / R_T$. For OOK, assuming a single-ended peak-to-peak voltage of V_{pp}, the voltage levels are $\pm V_{pp}/2$, yielding a mean square value of $E[a_k^2] = V_{pp}^2/4$. Because the differential drive consumes power across two signal lines, the total average power for OOK is given by

$$P_{OOK} = \frac{V_{pp}^2}{2R_T} \tag{5}$$

For PAM4, the four voltage levels are $\pm V_{pp}/2$ and $\pm V_{pp}/6$, resulting in a mean square value of $E[a_k^2] = 5V_{pp}^2/36$, and the total average power for PAM4 is given by

$$P_{\mathrm{PAM4}} = \frac{5V_{\mathrm{pp}}^2}{18R_{\mathrm{T}}} \quad (6)$$

The bit energy was obtained by dividing the average power by the bit rate.

Based on this update, we recalculated the energy consumption for both OOK and PAM4 and added the revised values to the main text.

Abstract lines 10–11: The fabricated modulator demonstrated 140-Gb/s OOK operation with an energy consumption of 26 fJ/bit using FinFET-CMOS-compatible drive voltages.

Main text lines 120–121: At this point, the energy required to drive the modulator corresponds to 43 fJ/bit.

Main text lines 130–134: The TDEC was improved to 1.5 dB at 128 Gb/s and 2.5 dB at 140 Gb/s, with a corresponding bit energy of 26 fJ/bit. For PAM4 modulation, the TDECQ was held to 1.5 dB even at 200 Gb/s with a corresponding bit energy of 63 fJ/bit. The remaining TDECQ margin suggests that higher data rates could be achieved under the same low-complexity FFE-only condition.

Main text lines 150–152: We have also confirmed that the device achieves an extremely low energy consumption of 26 fJ/bit with a CMOS-compatible drive voltage and has achieved 200 Gb/s PAM4 using only low-complexity electronic assistance.

2. 2) The Methods section refers to "optimized electrodes" and "optical delay lines" but lacks detailed geometric dimensions. The authors must specify the electrode and waveguide dimensions corresponding to the EO equalization time constants (T_{E}, T_{S}, T_{D}).

Furthermore, the authors should clarify how these constants are engineered and discuss their sensitivity to fabrication tolerances.

In response, we have revised the Methods section to describe how the electrode parameters and delay-line lengths were designed. Specifically, we clarified that the electrode parameters were designed to achieve the target impedance and electrical delay, and that the electrical delay line was designed following the same principle as in our previous work [R1-1]. We also specified the required time constants for the $N = 4$ device, $T_{\mathrm{E}} = T_{\mathrm{S}} = 4.3$ ps and $T_{\mathrm{D}} = 6.8$ ps, together with the corresponding PCW segment length, electrical delay-line length, and optical delay-line length.

Main text lines 176–186: The electrode parameters were designed using a method-of-moments solver (Keysight Momentum) to achieve the target impedance and electrical delay. The electrical delay line was designed following the same principle as in our previous work[30], where the coplanar-line geometry was chosen to satisfy both impedance matching and EO phase matching. Circuit simulations were performed in Keysight ADS using an in-house photonic device model library written in Verilog-A, enabling analysis of the EO frequency response and eye diagrams[44]. The model incorporates RC time constants, RF losses, EO phase mismatch, and optical loss. For the N = 4 device, the required time constants are $T_E = T_S$ = 4.3 ps and T_D = 6.8 ps. The PCW length is 64.3 µm per segment, with a 50-µm active p–n region, providing T_S = 4.3 ps with n_g = 20. The electrical delay line is designed with a length of 470 µm to ensure EO phase matching. The optical delay line, which includes the waveguide crossings and transition tapers, has a total length of approximately 480 µm.

We have also clarified the sensitivity to fabrication variations in Supplementary Information 2. In the present demonstration, we used a simple line-defect triangular-lattice PCW. Fabrication- and temperature-induced structural variations mainly shift the PCW n_g spectrum, thereby changing T_S. These spectrum shifts can be mitigated by using lattice-shifted PCWs with a flatter n_g spectrum. For example, our previous calculation showed that a lattice-shifted PCW with $n_g \approx 20$ can maintain an approximately constant n_g over a 35-nm wavelength range [R1-2]. In PCW modulators, the lattice-shift design has also enabled athermal operation over 19–124°C [R1-3]. Such a flatter n_g spectrum also helps preserve the EO-equalizer response. We have added this explanation as follows.

Supplementary information lines 51–55: Changes in the EO response caused by fabrication variations and temperature changes can be mitigated by using lattice-shifted PCWs with a flatter n_g spectrum. For example, previous calculations showed that a lattice-shifted PCW designed around $n_g \approx 20$ can provide an almost flat n_g spectrum over a 35-nm wavelength range [S4]. In PCW modulators, the lattice-shift design has also enabled athermal operation over 19–124°C [S5]. Such a flatter n_g spectrum also helps preserve the EO-equalizer response.

2. 3) The theoretical model or fitting procedure used for the 80 GHz bandwidth extrapolation must be provided.

In response, we have clarified the fitting procedure used for the 80-GHz bandwidth extrapolation in the main text. The 3-dB bandwidth was estimated by linearly extrapolating the measured EO

response in decibels from the highest measured frequency of 67 GHz. The slope used for the extrapolation was obtained by a linear fit to the smoothed data over the last 10 GHz of the measurement range, from 57 to 67 GHz. This procedure provides a direct and reproducible estimate of the 3-dB bandwidth from the measured high-frequency roll-off trend. The specific revision made to the manuscript is as follows.

Main text lines 209–212: To estimate the response up to 80 GHz, a linear extrapolation was performed from the highest measured frequency of 67 GHz. The slope for this extrapolation was determined by a linear fit to the smoothed data in decibels over the last 10 GHz of the measurement range, from 57 to 67 GHz.

2. 4) Additionally, please detail the derivation methods for modulation efficiency and power consumption.

The derivation method for the power consumption has been clarified in the Methods section, as described in our response to Comment 2-(1).

The derivation method for the modulation efficiency, $V_\pi L$, has been clarified in Supplementary Information 1. The $V_\pi L$ values were evaluated from low-data-rate extinction-ratio measurements using the $N = 3$ device without the EO equalizer. To avoid the influence of the frequency response, the extinction ratio was measured under OOK modulation at 2.4 Gb/s and quadrature bias with $V_{pp,diff} = 6.34$ V. We extracted V_π from the measured extinction ratio using the sinusoidal Mach–Zehnder transfer function, and then calculated $V_\pi L$ per phase shifter.

Supplementary information lines 14–21: Figure S1(c) shows the measured n_g spectrum of the PCW, and Fig. S1(d) shows the evaluated $V_\pi L$ per phase shifter versus wavelength. The $V_\pi L$ values in Fig. S1(d) were evaluated using the $N = 3$ device without the EO equalizer to characterize the intrinsic performance of the phase shifter. To avoid the influence of the frequency response, the extinction ratio was measured under OOK modulation at a low data rate of 2.4 Gb/s and quadrature bias with $V_{pp,diff} = 6.34$ V. We extracted V_π from the measured extinction ratio using the sinusoidal Mach–Zehnder transfer function, and then calculated $V_\pi L$ per phase shifter. The modulation efficiency improves at longer wavelengths due to the higher n_g, and the $V_\pi L$ reaches 0.16 V·cm at the longest wavelength, supporting CMOS-compatible low-voltage driving.

3. 1) Certain descriptions in the manuscript are ambiguous and require clarification. First, the

authors employ "AWG in-system calibration", "receiver equalization", and "16×/32× averaging"; it should be clarified whether these are considered part of the DSP.

We have clarified the roles of the AWG in-system calibration, the system-impulse-response correction filter, and the waveform averaging in the Methods section. The calibration procedures compensated only for the response of the measurement setup, and waveform averaging was used to suppress the ASE noise from the EDFA. These procedures were used only to correct the measurement-system response and were not used to equalize the modulator response. We have revised the Methods section as follows.

Main text lines 232–238: High-frequency losses in the electrical drive path, including the cables, drivers, and bias-tees, were compensated for up to 70 GHz using in-system calibration of the AWG. The intrinsic frequency response of the receiver module was corrected using a system-impulse-response correction filter. To suppress ASE noise from the EDFA, the acquired waveforms were averaged 16 times for OOK and 32 times for PAM4 modulation. These procedures were used only to correct the measurement-system response and were not used to equalize the modulator response.

3. 2) Second, the claim of CMOS compatibility is questionable; the OOK modulation requires a drive voltage of 2 V_{pp} and still relies on an external amplifier during testing. The statement "eliminates reliance on power-dissipating DSPs and drivers" is unsubstantiated.

As described in the General Response, we found and corrected a voltage calibration error in the original manuscript. After this correction, the revised data show lower drive voltages than those in the original manuscript, including DSP-free OOK operation at 100–112 Gb/s with V_{pp} = 1.3–0.7 V and 140-Gb/s OOK operation with an 11-tap receiver FFE at V_{pp} = 0.6 V. These results support the compatibility of the required voltage swing with advanced CMOS driver technologies.

In our setup, the use of external broadband amplifiers does not mean that the modulator required a large drive voltage. The AWG in-system calibration compensated for the high-frequency roll-off of the electrical drive path by reducing the low-frequency gain, which also reduced the overall signal amplitude. The external amplifiers were used to recover this calibration-induced amplitude reduction. The actual drive voltage applied to the modulator was set by the AWG output amplitude and was confirmed by the electrical eye diagram measurement. We have clarified this point in the Methods section as follows.

Main text lines 217–223: An arbitrary waveform generator (AWG), Keysight M8199B 256-GS/s,

generated electrical signals. After amplification using SHF M827B 70-GHz broadband amplifiers, the signals were delivered to the device through SHF BT110R 110-GHz bias-tees and a FormFactor 67-GHz GSGSG Infinity probe. The AWG in-system calibration compensated for the high-frequency roll-off of the electrical drive path by reducing the low-frequency gain. The external amplifiers were used to compensate for this amplitude reduction. The drive voltage applied to the modulator was controlled by adjusting the AWG output amplitude.

3. 3) Furthermore, the use of different devices for bandwidth (N = 3) and modulation (N = 4) experiments undermines the validity of the results. The authors should provide data from a unified device configuration to ensure consistency.

In this work, the $N = 3$ device was used to evaluate the maximum bandwidth enhancement enabled by the built-in EO equalizer, and its estimated 3-dB bandwidth reached 80 GHz. In contrast, the modulation experiments were performed using the $N = 4$ device because it provided a flatter EO response at the operating wavelength of 1537 nm. For high-speed data modulation, response flatness is more important than maximizing the 3-dB bandwidth alone, because response variation directly affects the output waveform quality. Although the $N = 4$ device did not provide the largest 3-dB bandwidth among the measured devices, its EO bandwidth exceeded the Nyquist requirement for 200-Gb/s PAM4 operation and its flatter response made it more suitable for the modulation experiments. Thus, the $N = 3$ device was used to demonstrate the bandwidth-extension capability of the EO equalizer, whereas the $N = 4$ device was used to demonstrate high-speed data modulation with an EO response better suited to preserving waveform quality. To support this clarification, we added the EO frequency response of the $N = 4$ device used for the modulation experiments to Fig. 3(c) in the revised manuscript. In addition, we clarified this discussion in the main text as follows.

Figure 3: We have added the EO frequency responses of the $N = 4$ device as follows:

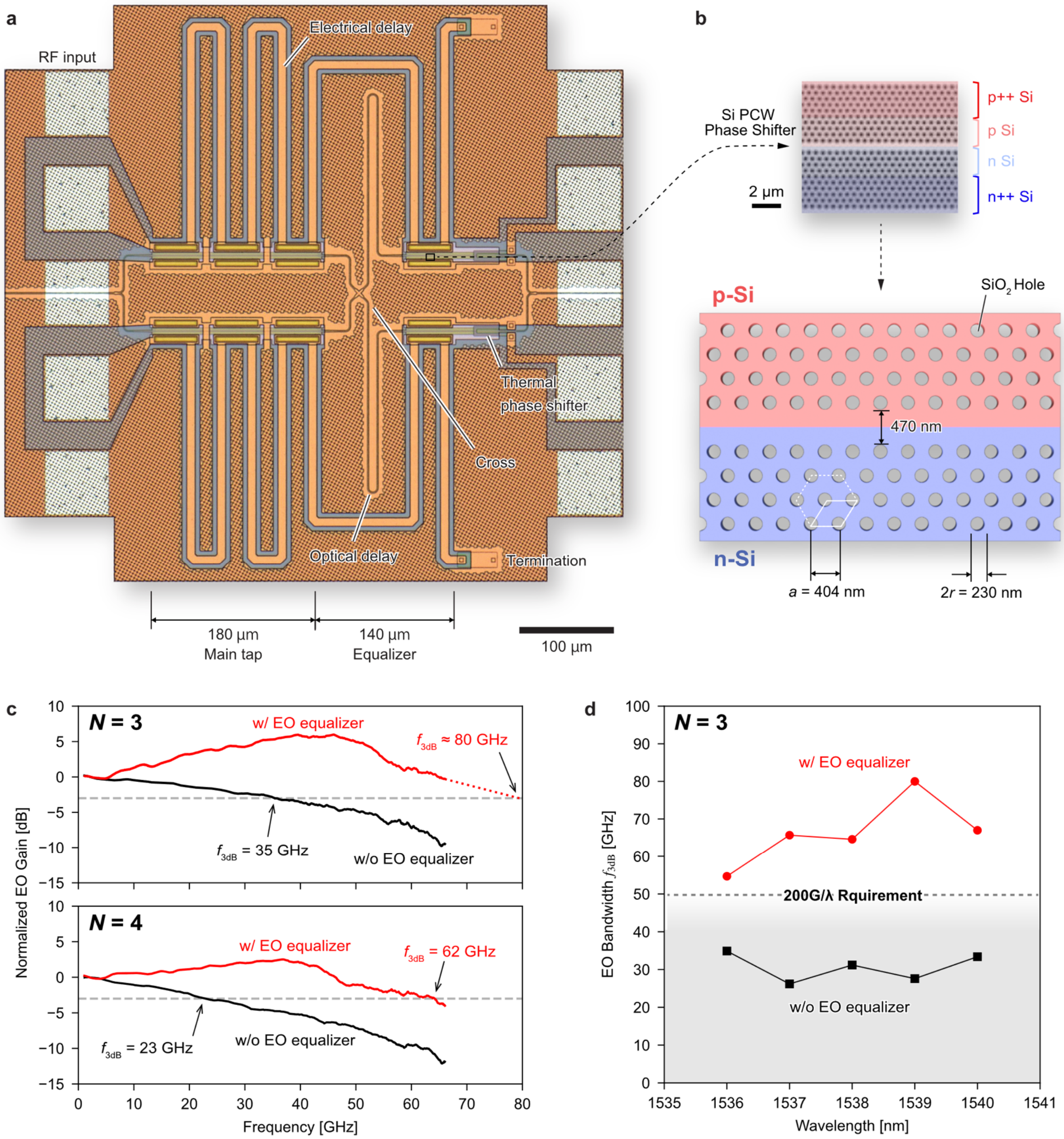

Fig. 3 Fabricated modulator. (a) Optical micrograph. (b) Laser confocal micrograph and an enlarged view of the Si PCW phase shifter. (c) Measured normalized EO gain for $N = 3$ and 4 devices, demonstrating bandwidth enhancement from 35 GHz to 80 GHz for $N = 3$ and from 23 GHz to 62 GHz for $N = 4$ with the EO equalizer. (d) Measured 3-dB bandwidth of the $N = 3$ device across the operating wavelength range of the PCW exceeding the requirement for 200 Gb/s/λ PAM4 transmission.

Main text lines 97–103: Figure 3c shows the measured frequency responses of the $N = 3$ and $N = 4$ devices, with and without the EO equalizer (See Methods section for the experimental details).

Consistent with the peak-intensity tuning discussed in Fig. 2c, the $N = 3$ device provided stronger high-frequency compensation and increased the 3-dB bandwidth from 35 GHz to 80 GHz. The $N = 4$ device provided a more moderate equalization response, increasing the 3-dB bandwidth from 23 GHz to 62 GHz with a flatter EO response. Figure 3d summarizes the measured 3-dB bandwidths of the $N = 3$ device for wavelengths from 1536 nm to 1540 nm.

Main text lines 108–110: ..., we experimentally confirmed that the Si PCW modulator with a built-in EO equalizer operates up to 200 Gb/s PAM4. The modulation experiments were performed using the $N = 4$ device because of its flatter EO response, as shown in Fig. 3c.

In addition, we revised the Abstract and Introduction to avoid a mismatch between the bandwidth-enhancement result and the device length. In the Abstract, the device length (320 μm for the $N = 4$ device) was moved from the bandwidth-enhancement statement to the following sentence describing the fabricated modulator. In the Introduction, the device-length phrase was removed from the bandwidth-enhancement statement, and the modulation experiments were clarified as being performed using the device optimized for response flatness.

Abstract lines 9–10: By monolithically integrating optical delay lines and crossings within segmented slow-light phase shifters, we synthesized an on-chip feedforward response that extends the 3-dB bandwidth from 35 GHz to 80 GHz ~~with a small device length of 320 μm~~. The fabricated modulator with a small device length of 320 μm demonstrated 140-Gb/s OOK operation with an energy consumption of 26 fJ/bit using FinFET-CMOS-compatible drive voltages.

Main text lines 43–46: We demonstrated a Si modulator with a compact device length ~~of only 320 μm~~, while extending the intrinsic EO bandwidth from 35 GHz to 80 GHz. In modulation experiments using the device optimized for response flatness, we achieved 140-Gb/s OOK using FinFET-CMOS-compatible low-voltage driving, and 180-Gb/s PAM4 without DSP equalization.

4. In the Discussion section, the authors assert that the proposed modulator is suitable for WDM applications; however, this claim is undermined by critical performance bottlenecks. Specifically, the usable slow-light bandwidth of the proposed modulator is severely limited (~6 nm), and the on-chip insertion loss is prohibitive (> 10 dB). Furthermore, the device exhibits significant performance non-uniformity across the spectrum; notably, the modulation efficiency varies by a factor of more than three within the bandwidth. Additionally, the inherent sensitivity of photonic crystal structures to fabrication tolerances poses a scalability challenge. A rigorous and

comprehensive analysis reconciling these limitations with the claimed WDM compatibility is mandatory.

In the present device, we used a simple line-defect triangular-lattice PCW. This PCW showed a 6-nm optical passband with wavelength-dependent n_g. This wavelength dependence changes the slow-light delay T_S and the tap delay T_D, thereby affecting the response of the EO equalizer. Nevertheless, the measured f_{3dB} exceeded 50 GHz over the measured wavelength range for both N = 3 and N = 4, supporting 200-Gb/s PAM4 operation within this range. This result indicates that the wavelength dependence of the present PCW does not prevent high-speed operation within its optical passband. For WDM scaling, multiple PCW modulators designed for different wavelength ranges can be integrated. In addition, lattice-shifted PCWs with a broader spectral window and a flatter n_g spectrum can be applied to suppress the wavelength dependence of T_S and T_D. Therefore, the present PCW design does not preclude WDM operation. We revised the Discussion and Supplementary Information to clarify these points. Regarding fabrication tolerance, the detailed mechanism was already addressed in our response to Comment 2. 2).

Main text lines 160–164: In the present device, we used a simple line-defect triangular-lattice PCW, and the measured spectral window was 6 nm (See Supplementary Information 1). The available wavelength range can be broadened either by integrating multiple PCW modulators, each designed to operate over a different wavelength range, or by using lattice-shifted PCWs with a broader spectral window.

Supplementary information lines 42–55: Figure S3 and Figure S4 show the measured EO frequency responses of the PCW modulator using an EO equalizer for N = 3 and 4, respectively. Because the simple line-defect triangular-lattice PCW has a wavelength-dependent n_g, shifts of the n_g spectrum caused by fabrication variations or temperature drift change n_g at the operating wavelength. This changes the EO phase matching and the tap delay T_D of the equalizer, and consequently modifies the EO response. While phase mismatch typically narrows the bandwidth in conventional traveling-wave modulators, it can induce high-frequency peaking when an EO equalizer is employed. In Figure S3, the bandwidth for N = 3 was maximized at 1539 nm, where the estimated f_{3dB} reached 80 GHz. The f_{3dB} exceeded 50 GHz at all other wavelengths for both N = 3 and 4, which supports 200 Gb/s PAM4 operation. The modulation experiment was conducted using the N = 4 device at 1537 nm, which was selected for its superior flatness. Changes in the EO response caused by fabrication variations and temperature changes can be mitigated by using lattice-shifted PCWs with

a flatter n_g spectrum. For example, previous calculations showed that a lattice-shifted PCW designed around $n_g \approx 20$ can provide an almost flat n_g spectrum over a 35-nm wavelength range [S4]. In PCW modulators, the lattice-shift design has also enabled athermal operation over 19–124°C [S5]. Such a flatter n_g spectrum also helps preserve the EO-equalizer response.

The relatively large insertion loss is related to the same slow-light characteristics. A larger n_g enhances phase modulation efficiency and reduces $V_\pi L$, but it also increases carrier absorption in the p-n-doped PCW. This trade-off should be further addressed by improving the PCW structure and doping condition. For example, periodic placement of the p-n junction can increase the overlap between the depletion region and the slow-light mode, thereby improving modulation efficiency without changing the doping conditions [S2]. We clarified this discussion in the Supplementary Information 1.

Supplementary information lines 21–25: The increase in n_g also causes carrier absorption in the p-n-doped PCW, which should be addressed by improving the PCW structure and doping condition. For example, periodic placement of the p-n junction can increase the overlap between the depletion region and the slow-light mode [S2], thereby maintaining modulation efficiency even at lower doping levels.

5. How does N affect the frequency response of the modulator without an EO equalizer in Fig. S2, since the phase shifter length remains constant?

We have clarified the effect of N on the intrinsic EO frequency response in Supplementary Information 2 as follows.

Supplementary information lines 27–41: Figure S2 shows the simulated intrinsic EO frequency response of the PCW modulator without an EO equalizer where the phase shifter length is fixed at 250 μm. The frequency response of the modulator without an equalizer is fundamentally determined by the RC time constant, the phase mismatch between the RF signal and slow light within a segment, and the propagation loss of the RF signal. The theoretical background is discussed in our previous paper [S3], and the normalized electro-optic frequency response $G_{EO}(\omega)$ is expressed by

$$G_{\mathrm{EO}}(\omega) = \left|\frac{1}{1 + j\omega R_{\mathrm{pn}} C_{\mathrm{pn}}}\right| \cdot \frac{1 - e^{-N\alpha_{\mathrm{RF}} l_{\mathrm{U}}}}{N(1 - e^{-\alpha_{\mathrm{RF}} l_{\mathrm{U}}})}, \quad \text{(S1)}$$

where R_{pn} is the resistance of the pn junction, C_{pn} is the capacitance of the pn junction, α_{RF} is the

propagation loss of the RF signal, and l_U is the length of each segment. When N increases while the total length of the phase shifter remains constant, the length of each segment l_U becomes proportionally shorter. As indicated in the equation, the roll-off caused by the phase mismatch between the RF signal and slow light within the segment depends on l_U. Therefore, the shortened segment length mitigates this phase mismatch. As a result, increasing N reduces the effect of the sinc roll-off in the high-frequency region, which improves the overall frequency response. Figure S2(b) summarizes the simulated f_{3dB} and f_{6dB} as a function of N. The f_{3dB} is saturated at $N = 2$ while the f_{6dB} is enhanced by increasing N. The more gradual roll-off and improved f_{6dB} at larger N values make the response easier to compensate with an EO equalizer and enable a broad bandwidth.

References

[R1-1]. Kawahara, K. et al. High-speed, low-voltage, low-bit-energy silicon photonic crystal slow-light modulator with impedance-engineered distributed electrodes. *Optica* **11**, 1212–1219 (2024).

[R1-2]. Hirotani, K., Shiratori, R. & Baba, T. Si photonic crystal slow-light waveguides optimized through informatics technology. *Opt. Lett.* **46**, 4422 (2021).

[R1-3]. Nguyen, H. C. et al. Sub-100 μm Photonic Crystal Si Optical Modulators: Spectral, Athermal, and High-Speed Performance. *IEEE J. Sel. Top. Quantum Electron.* **19**, 127–137 (2013).

Response to Reviewer #2:

Key results

The authors report a slow-light silicon photonic crystal waveguide (PCW) Mach–Zehnder modulator with a built-in electro-optic feed-forward equalizer extend the 3-dB bandwidth by a >2x factor. The buit-in electro-optic equalizer is the primary innovation reported. The device is presented as a compact, low-voltage silicon modulator architecture that improves EO bandwidth while reducing dependence on electronic equalization. The manuscript reports an EO 3-dB bandwidth increase from ~35 GHz to ~80 GHz for the demonstrated condition, DSP-free eye openings up to 180 Gb/s PAM4, and 200 Gb/s PAM4 operation with a low-complexity 11-tap receiver FFE.

We appreciate the reviewer's valuable comments on our manuscript. We have carefully addressed all points raised by the reviewer and have revised the manuscript accordingly. Our detailed responses to the points are provided below.

Validity

The device concept is interesting and the measurements are promising. However, the current evidence is not yet sufficient to substantiate deployment-level DSP-independent performance for practical CPO based links, even though the manuscript reports DSP-independent high-speed operation demonstrations.

A central issue is that the manuscript primarily uses eye diagrams and TDEC/TDECQ-style metrics to support DSP-free or DSP-light feasibility, but it does not provide BER vs OMA (or BER vs received power) data. For a 200 Gb/s/λ Intensity Modulation (IM) Direct Detection (DD) transmitter claim, BER-based evidence is important to connect the demonstrated eye quality to FEC-relevant operating margins and realistic link budgets.

The manuscript also frames TDEC/TDECQ results using a Soft Detection (SD) FEC threshold of BER = 2×10^{-2}. Because the target application is IM/DD OOK/PAM4 transmission, the authors should justify the relevance of this threshold to the intended use case and/or provide comparison to a direct-detect relevant baseline such as Ethernet 802.3.

Finally, while the voltage swings are described as advanced CMOS technology compatible, the experimental setup relies on a 256-GS/s AWG and 70-GHz high-voltage broadband amplifiers.

This does not invalidate the device result, but it does leave open an important practical question: what bandwidth/voltage/reliability constraints would apply in a realizable CMOS driver implementation at the claimed rates?

Because the points raised here are presented again as specific requests in the Suggested improvements, we have addressed them individually in the corresponding responses below.

Specifically, the point regarding BER-versus-OMA or BER-versus-received-power data is addressed in our response to Suggested improvement 1. The FEC reference used for the TDEC/TDECQ evaluation is addressed in Suggested improvement 3. The practical driver constraints associated with the AWG and 70-GHz-amplifier-based setup are addressed in Suggested improvement 5.

Significance

Reducing DSP and driver burden at 200 Gb/s/λ is an important problem, and integrating an EO equalizer into the modulator is a potentially valuable device-level approach. The manuscript therefore has potential significance.

However, the practical significance is difficult to assess fully without:

- BER vs OMA (or BER vs received power),
- device-level insertion loss (all frequency plots are normalized to 0dB),
- and a clearer application-appropriate FEC framing.

These additions would allow readers to better judge whether the work supports deployment-level DSP-free performance, or more conservatively, a “DSP-light” transmitter and/or receiver architecture.

Because the remaining points raised here are presented again as specific requests in the Suggested improvements, we have addressed them individually in the corresponding responses below.

Specifically, the point regarding BER-versus-OMA or BER-versus-received-power data is addressed in our response to Suggested improvement 1. The point regarding device insertion loss is addressed in Suggested improvement 4. The FEC reference used for the TDEC/TDECQ evaluation is addressed in Suggested improvement 3. The distinction between “DSP-free” operation is addressed in Suggested improvement 2.

Data and methodology

The experiments use a Keysight M8199B AWG (256 GS/s), 70 GHz broadband amplifiers, and a high speed optical receiver; eyes and TDEC/TDECQ are reported for OOK and PAM4. This methodology is reasonable for device level characterization, however, the absence of BER vs OMA and device insertion loss prevents translation of the measurements into deployable link budgets.

Because the points raised here are presented again as specific requests in the Suggested improvements, we have addressed them individually in the corresponding responses below.

Specifically, the point regarding BER-versus-OMA data is addressed in our response to Suggested improvement 1. The point regarding device insertion loss is addressed in Suggested improvement 4.

Analytical approach

The manuscript provides a convincing device concept and amplitude-response-oriented EO characterization. To strengthen the analytical case for DSP-free (or DSP-reduced) operation, I suggest the authors connect the measured EO response more directly to system-level impairments. In particular, if phase/group-delay effects are expected to contribute to pattern-dependent timing distortion, this should be discussed and, if possible, characterized. Even a concise discussion would help readers understand what the integrated EO equalizer corrects effectively and what residual impairments may remain or enhanced.

We agree that this point is important because phase and group-delay responses can affect time-domain waveform quality, including pattern-dependent jitter.

As discussed in our previous report [R2-1], an appropriately designed Si PCW modulator intrinsically exhibits a favorable group-delay response. In our simulation, the Si PCW modulator without the built-in EO equalizer shows a small Nyquist-range group-delay variation of approximately ±1.2 ps for the $N = 3$ device and ±1.4 ps for the $N = 4$ device. The present built-in EO equalizer was designed primarily to reduce the high-frequency amplitude roll-off of the modulator. As a result, the Nyquist-range group-delay variation is somewhat increased to approximately ±2.4 ps for the $N = 3$ device and ±3.2 ps for the $N = 4$ device. However, the substantial reduction of the high-frequency roll-off suggests that the built-in EO equalizer provides a net benefit to the transmitter waveform quality, even when this increase in group-delay variation

is considered.

The effect of group-delay variation on the time-domain waveform, including pattern-dependent jitter, is also included in the measured TDEC/TDECQ values. The histograms used for the TDEC and TDECQ measurements were intentionally placed away from the center region of the eye diagram. Specifically, two 0.04-UI-wide histograms separated by 0.2 UI were used for TDEC measurements, whereas two 0.04-UI-wide histograms separated by 0.1 UI were used for TDECQ measurements. This histogram placement makes the TDEC/TDECQ values sensitive to timing jitter and other time-domain waveform degradations. Even under this evaluation condition, the TDEC/TDECQ values were improved by the built-in EO equalizer, as shown in Figs. 4b, 4c, and S7. We have added this explanation of the TDEC/TDECQ evaluation procedure to Supplementary Information 3.

Supplementary information lines 56–67: Figure S5 shows the simulated group-delay frequency responses of the $N = 3$ and $N = 4$ devices with and without the built-in EO equalizer. As discussed in our previous report [S3], an appropriately designed Si PCW modulator intrinsically exhibits a favorable group-delay response. In our simulation, the Si PCW modulator without the built-in EO equalizer shows a small Nyquist-range group-delay variation of approximately ±1.2 ps for the $N = 3$ device and ±1.4 ps for the $N = 4$ device. The present built-in EO equalizer was designed primarily to reduce the high-frequency amplitude roll-off of the modulator. As a result, the Nyquist-range group-delay variation is somewhat increased to approximately ±2.4 ps for the $N = 3$ device and ±3.2 ps for the $N = 4$ device. However, the substantial reduction of the high-frequency roll-off suggests that the built-in EO equalizer provides a net benefit to the transmitter waveform quality, even when this increase in group-delay variation is considered. In addition, the effect of group-delay variation on the time-domain waveform, including pattern-dependent jitter, is reflected in the measured TDEC and TDECQ values by appropriately setting the histogram positions, as discussed in Supplementary Information 3.

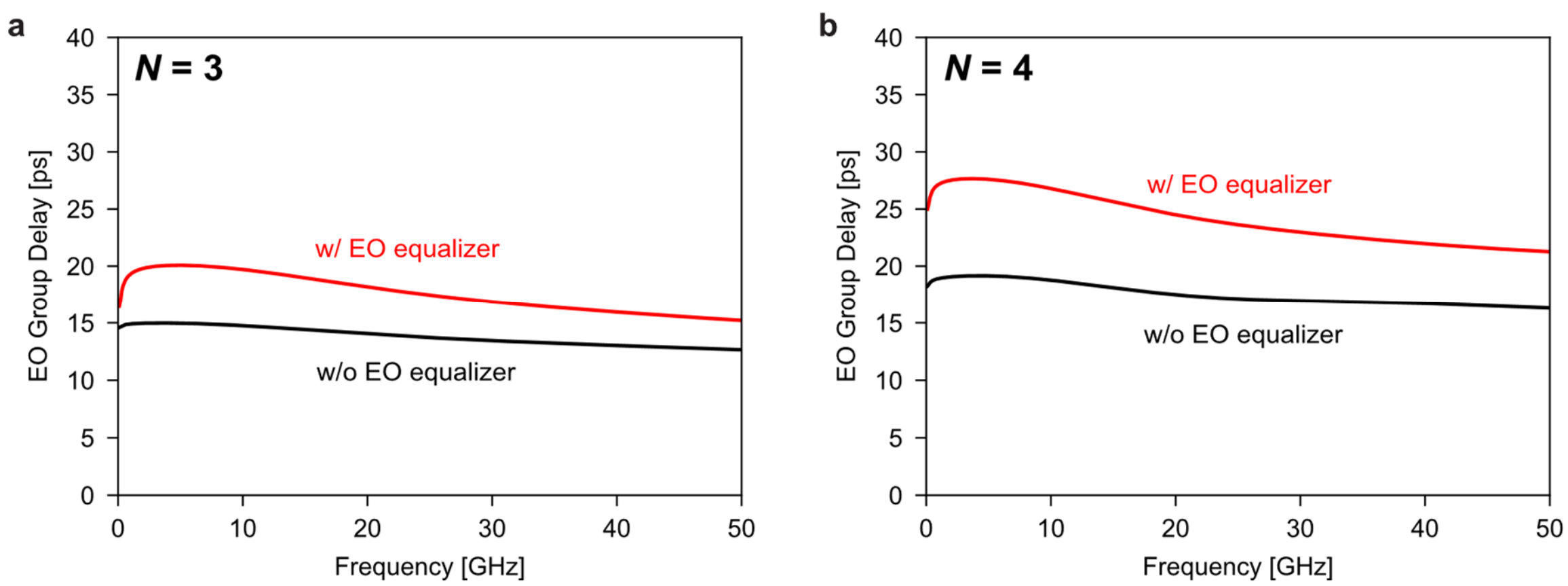

Fig. S5 Simulated EO group-delay frequency responses of the PCW modulators with and without the built-in EO equalizer for (a) N = 3 and (b) N = 4 devices.

Supplementary information lines 69–75: Figure S6 shows the TDEC and TDECQ measurement configurations used in this work. To evaluate the waveform quality while capturing the effect of time-domain waveform degradation, such as pattern-dependent jitter, we intentionally placed the histograms used for the TDEC and TDECQ measurements away from the center region of the eye pattern. Specifically, two 0.04-UI-wide histograms separated by 0.2 UI were used for TDEC measurements, whereas two 0.04-UI-wide histograms separated by 0.1 UI were used for TDECQ measurements. This histogram placement makes the TDEC and TDECQ values sensitive to timing jitter and other time-domain waveform degradations.

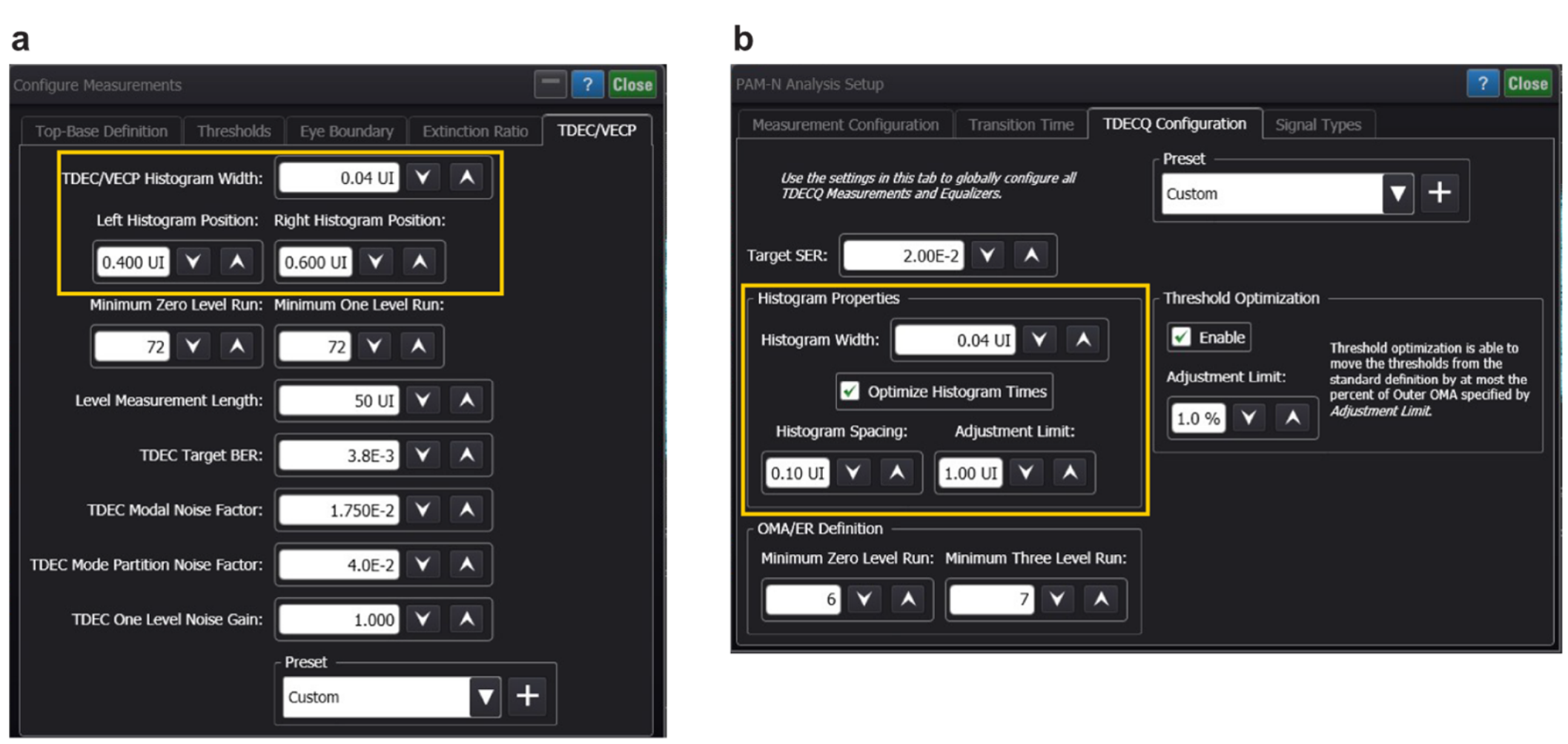

Fig. S6 Measurement configurations for (a) TDEC and (b) TDECQ. The yellow boxes indicate the histogram width and position settings.

Suggested improvements

1. Add BER vs OMA (or BER vs received power) at the demonstrated OOK/PAM4 operating points, especially for the headline high-rate cases. This is the most important missing evidence for evaluating practical performance and FEC margin.

We agree that such measurements provide important evidence for assessing the practical performance and FEC margin of a complete IM/DD link.

The central claim of this work, however, is different from an absolute FEC-margin demonstration. The built-in EO equalizer is intended to reduce the transmitter-induced waveform penalty caused by the limited EO response of the Si PCW modulator. The quantity affected most directly by this mechanism is therefore not the BER of a complete link, but the degradation of the transmitted optical waveform relative to an ideal transmitter waveform. TDEC and TDECQ express this degradation as an equivalent power penalty using a defined reference receiver, and are therefore the appropriate metrics for comparing the modulator output with and without the EO equalizer under identical evaluation conditions. In contrast, a BER-vs-OMA curve is essential for establishing the FEC margin of a complete transceiver link, but the measured BER also depends on the receiver condition and other link-dependent factors. For this reason, the present manuscript uses TDEC and TDECQ to support the specific claim that the built-in EO equalizer improves the transmitter waveform quality by reducing the transmitter-induced penalty.

To avoid overstatement, we have revised the manuscript so that the reported results are not presented as a direct demonstration of complete-link performance. We now clarify that the demonstrated improvement is a transmitter-waveform improvement quantified by TDEC/TDECQ, and that BER-based characterization will be required in future work to determine the practical performance and FEC margin in an IM/DD transceiver link.

Main text lines 239–244: We used TDEC and TDECQ to compare the transmitter waveform quality with and without the EO equalizer under a common measurement procedure, thereby quantifying the reduction in waveform penalty achieved by the built-in EO equalizer. TDEC and TDECQ were evaluated using the FlexDCA software, with an SD-FEC threshold of $BER = 2 \times 10^{-2}$ as the reference. While these metrics are suitable for transmitter characterization, evaluation of transceiver links will ultimately require BER-based measurements.

Abstract lines 13–15: By eliminating reliance on power-dissipating electronics, this strategy ~~enables~~ provides a route toward dense multi-Tb/s co-packaged optics in future energy-constrained switching systems.

Main text lines 44–46: In transmission modulation experiments using the device optimized for response flatness, we achieved 140-Gb/s OOK using FinFET-CMOS-compatible low-voltage driving, and 180-Gb/s PAM4 without DSP equalization.

Main text lines 128–129: The data rate was further extended in practical optical interconnections, with a using a low-complexity 11-tap symbol-spaced FFE at the receiver, indicating the potential for practical optical interconnects.

Figure 4 caption: Data transmission results. Measured optical modulation characteristics.

2. Clarify the "DSP-free" / "DSP-independent" claim and align the claim language with the evidence.

In this manuscript, "DSP-free" means that no FFE or other digital waveform equalization is applied to the measured optical waveform. "DSP-independent" means that the main bandwidth compensation is performed in the modulator by the built-in EO equalizer, rather than by high-complexity electronic DSP.

We have revised the Results section to make this distinction clearer. In the DSP-free measurement, the PAM4 eye opening was clearly maintained up to 180 Gb/s. At 200 Gb/s, a PAM4 eye opening was still observed, but the TDECQ increased to 4.9 dB. We therefore revised the wording for the 200-Gb/s case to state that the eye opening was observed before applying the receiver FFE, while the 200-Gb/s PAM4 result with improved waveform quality is described as operation with a low-complexity 11-tap receiver FFE. This revision avoids overstating the DSP-free result while preserving the main conclusion that the built-in EO equalizer reduces the required electronic equalization.

Main text lines 107–109: To demonstrate high-speed optical modulation far beyond the intrinsic device bandwidth without any DSP without relying on high-complexity DSP, we experimentally confirmed that the Si PCW modulator with a built-in EO equalizer operates up to 200 Gb/s PAM4.

Main text lines 126–127: Even at 200 Gb/s, although the TDECQ rose to 4.9 dB, a clear eye opening was still observed, indicating that the Si PCW modulator with the built-in EO equalizer maintains DSP-free PAM4 operation at this data rate.

3. Justify the FEC baseline used for TDEC/TDECQ interpretation (SD-FEC BER = 2×10^{-2}), or provide an additional comparison using a direct-detection relevant baseline for the intended

application.

We agree that, for an IM/DD OOK/PAM4 application, it is important to discuss the results not only with respect to an SD-FEC reference of $BER = 2 \times 10^{-2}$ but also with respect to a more conservative direct-detection-relevant FEC baseline.

In the original manuscript, $BER = 2 \times 10^{-2}$ was used as the main reference because SD-FEC thresholds in this range are commonly adopted in proof-of-concept demonstrations of high-speed PAM4 optical links. As discussed in our response to Suggested improvement 1, the purpose of the present TDEC and TDECQ evaluation is to assess the device-level effect of the built-in EO equalizer on the transmitter waveform quality, and it was not intended as a compliance test for a specific communication standard such as Ethernet 802.3. Therefore, rather than applying the detailed conditions of a specific standard, we considered it appropriate to compare the results using general FEC references relevant to high-speed direct-detection optical links. Following this rationale, we additionally evaluated the TDEC and TDECQ results using the more conservative HD-FEC threshold of $BER = 3.8 \times 10^{-3}$. The additional analysis confirms that the built-in EO equalizer remains effective under this stricter reference. In particular, for PAM4 modulation, TDECQ < 3 dB was maintained up to 160 Gb/s without DSP and up to 200 Gb/s with the 11-tap FFE.

In the revised manuscript, we clarified in the Methods section that SD-FEC with $BER = 2 \times 10^{-2}$ was used as the reference for the main TDEC/TDECQ evaluation, and we added the corresponding TDEC/TDECQ results using the HD-FEC threshold of $BER = 3.8 \times 10^{-3}$ to the Supplementary Information.

Supplementary information lines 75–78: Figure S7 shows the TDEC and TDECQ results evaluated using the more conservative HD-FEC threshold of BER = 3.8×10^{-3}. The results show that the built-in EO equalizer remains effective even under this stricter reference. In particular, for PAM4 modulation, TDECQ < 3 dB was maintained up to 160 Gb/s without DSP and up to 200 Gb/s with the 11-tap FFE.

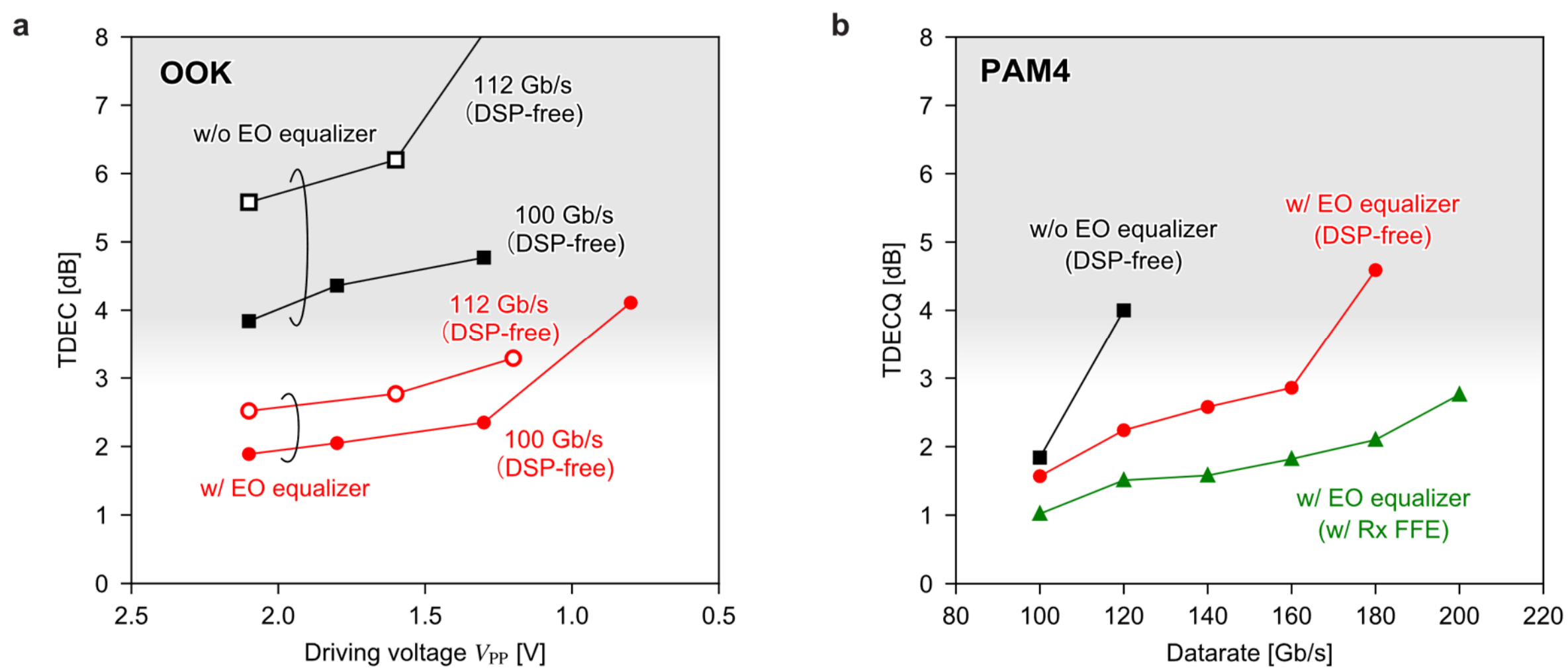

Fig. S7 TDEC and TDECQ evaluated using the HD-FEC threshold of BER = 3.8×10^{-3}. (a) Measured TDEC versus drive voltage for OOK signals with and without the built-in EO equalizer. (b) TDECQ versus data rate for PAM4 signals.

4. Report insertion loss. This is necessary to assess the practical value of the demonstrated EO bandwidth enhancement.

We have added the measured on-chip optical insertion losses to Supplementary Information 1, where the static optical characteristics are described. For the $N = 3$ device used to demonstrate the largest bandwidth enhancement, the losses were 10.1 dB without the EO equalizer and 11.7 dB with the EO equalizer. For the $N = 4$ device used in the modulation experiment, the corresponding values were 13.4 dB and 15.5 dB.

We have also clarified the origin and implication of the loss in the revised Supplementary Information 1. The increase in n_g also enhances carrier absorption in the p-n-doped PCW, indicating a trade-off between modulation efficiency and optical loss. This trade-off should be further addressed by improving the PCW structure and doping condition. For example, periodic placement of the p-n junction can increase the overlap between the depletion region and the slow-light mode [S2], thereby maintaining modulation efficiency even at lower doping levels.

Supplementary information lines 3–6: The measured on-chip insertion losses of the $N = 3$ device were 10.1 dB without the EO equalizer and 11.7 dB with the EO equalizer. For the $N = 4$ device used in the modulation experiment, the corresponding insertion losses were 13.4 dB and 15.5 dB, respectively.

Supplementary information lines 21–25: The increase in n_g also causes carrier absorption in the p-n-doped PCW, which should be addressed by improving the PCW structure and doping condition.

For example, periodic placement of the p-n junction can increase the overlap between the depletion region and the slow-light mode [S2], thereby maintaining modulation efficiency even at lower doping levels.

5. Discuss driver feasibility more explicitly in the context of the AWG + 70-GHz amplifier-based setup. A short discussion of expected driver constraints (swing, bandwidth, reliability/device stress) would improve realism without requiring a full electronic co-design demonstration.

For a CMOS driver, the relevant requirements are the output swing and electrical bandwidth. In the present 200-Gb/s PAM4 experiment, the required drive swing at the modulator input was V_{pp} = 1.5 V, and the corresponding symbol rate was 100 Gbaud. This voltage swing can be handled by advanced CMOS drivers using cascode output stages to avoid device overvoltage stress [R2-2]. The bandwidth requirement is also consistent with recent 5-nm FinFET driver demonstrations operating at 106.25 Gbaud [R2-3]. This result closely matches both the voltage swing and symbol rate required for the present 200-Gb/s PAM4 experiment. We added this short discussion of output swing and bandwidth to the Discussion section.

Main text lines 153–159: The achievement of such a high-speed and low-power consumption modulator platform is important because it eliminates reliance on power-intensive DSPs and external drivers. For practical CMOS driver implementation, the main constraints are the output swing and electrical bandwidth. In the present 200-Gb/s PAM4 experiment, the required drive swing at the modulator input was V_{pp} = 1.5 V, and the corresponding symbol rate was 100 Gbaud. This voltage swing can be handled by advanced CMOS drivers using cascode output stages to avoid device overvoltage stress[41]. The bandwidth requirement is also consistent with recent 5-nm FinFET driver demonstrations operating at 106.25 Gbaud[42].

6. Address temperature robustness / calibration sensitivity of the built-in optical equalizer. Because the manuscript is motivated by practical deployment, it would help to clarify how temperature drift affects equalizer timing/response and whether any compensation is required during operation. This may require additional DSP to allow for dynamic calibration to compensate for temperature drift.

Temperature drift changes the refractive index of Si and shifts the PCW transmission spectrum to longer wavelengths. In our previous co-packaged transmitter experiment, driver-induced heating shifted the PCW spectrum by 2.6 nm, while the 3-dB operating bandwidth of the PCW was 5.9 nm

[R2-4]. This result showed that a PCW modulator can operate without laser wavelength tuning or active temperature control when the spectral shift remains within the operating band.

In this work, we used a simple line-defect triangular-lattice PCW, whose n_g has wavelength dependence, as shown in Fig. S1(c). Therefore, a temperature-induced spectral shift changes n_g at the operating wavelength and hence changes T_S and T_D, producing the wavelength-dependent EO frequency response variation shown in Figs. S3 and S4. Nevertheless, both the $N = 3$ and $N = 4$ devices maintained $f_{3dB} > 50$ GHz over the measured wavelength range, indicating that the present device preserves sufficient bandwidth for 200-Gb/s PAM4 operation within this range. This temperature sensitivity can be further reduced by using lattice-shifted PCWs [R2-5]. As shown in our previous calculation, a lattice-shifted PCW with $n_g \approx 20$ can maintain an approximately constant n_g over a 35-nm wavelength range [R2-6]. Therefore, by using such a flat-n_g PCW design, the wavelength dependence of T_S and T_D can be suppressed, and the temperature-induced change in the EO equalizer response itself can be reduced. Thus, additional electronic DSP is not inherently required for dynamic calibration of the EO equalizer response. We clarified this point in Supplementary Information 2.

Supplementary information lines 8–12: Nevertheless, the 6-nm spectral window is sufficiently wide to eliminate the need for temperature compensation. In our previous co-packaged transmitter experiment, driver-induced heating shifted the PCW spectrum by 2.6 nm, while the 3-dB operating bandwidth of the PCW was 5.9 nm. This result showed that a PCW modulator can operate without laser wavelength tuning or active temperature control when the spectral shift remains within the operating band [S1].

Supplementary information lines 42–46: Figure S3 and Figure S4 show the measured EO frequency responses of the PCW modulator using an EO equalizer for $N = 3$ and 4, respectively. Because the simple line-defect triangular-lattice PCW has a wavelength-dependent n_g, shifts of the n_g spectrum caused by fabrication variations or temperature drift change n_g at the operating wavelength. This changes the EO phase matching and the tap delay T_D of the equalizer, and consequently modifies the EO response.

Supplementary information lines 51–55: Changes in the EO response caused by fabrication variations and temperature changes can be mitigated by using lattice-shifted PCWs with a flatter n_g spectrum. For example, previous calculations showed that a lattice-shifted PCW designed around $n_g \approx 20$ can provide an almost flat n_g spectrum over a 35-nm wavelength range [S4]. In

PCW modulators, the lattice-shift design has also enabled athermal operation over 19–124°C [S5]. Such a flatter n_g spectrum also helps preserve the EO-equalizer response.

7. Improve CPO context with quantitative comparisons. A comparison on bandwidth density, total link power (with clear accounting), and PAM4 linearity/DSP requirements relative to representative alternatives (such as Silicon Photonics ring-based resonators) would help readers evaluate system-level impact.

In response, we have added Table 1 to the main text and revised the Discussion to strengthen the CPO context through a quantitative comparison with representative silicon modulators, including both MZM- and MRM-based devices.

For bandwidth density, we added the EO bandwidth together with the phase-shifter length. This comparison clarifies that recent modulators have exceeded 200 Gb/s, although this progress has depended on high drive voltages, long phase shifters, and extensive DSP. MRMs provide compact footprints and low bit energy, whereas their resonant operation requires wavelength control. In contrast, the present device achieves a broad EO bandwidth with a deep sub-millimeter PCW phase shifter, retaining the non-resonant behavior.

For PAM4 linearity and DSP requirements, we added the DSP-free data rate and the equalization method used for DSP-assisted operation. Previous high-speed PAM4 demonstrations often rely on complex DSP, including long-tap equalization, predistortion, nonlinear equalization, or neural-network-based equalizers, depending on the modulator architecture. In contrast, the present device achieves the highest DSP-free data rate in comparison and reaches 200-Gb/s PAM4 operation using only low-complexity electronic assistance. We clarified this point in the revised Discussion.

Regarding total link power, we agree that it is important for evaluating CPO systems. However, a meaningful total-link-power comparison requires complete link-level assumptions; otherwise, the comparison can easily become arbitrary or misleading. Therefore, we focused on directly comparable quantities in Table 1, including the driving bit energy and the DSP condition. These additions allow readers to evaluate the system-level implication of the proposed modulator from explicit device-level metrics.

<u>Main text lines 136–152</u>: It has been recognized for years that Si photonics is a primary candidate for multi-Tb/s interconnects, yet the trade-off between modulation efficiency and operating

bandwidth remains a fundamental bottleneck. As shown in Table 1, recent modulators have exceeded 200 Gb/s, although this progress has depended on high drive voltages[35,45,46], long phase shifters[47,48], and extensive DSP[14,17,35,45-50]. MRMs provide compact footprints and low switching energy, whereas their resonant operation requires wavelength control[17,49,50]. In this article, we have shown that integrating a built-in EO equalizer with a Si PCW slow-light modulator is essential to overcome this trade-off without relying on DSP. The extraordinary modulation efficiency of the slow-light effect is critical for device miniaturization, which reduces the active length to the deep sub-millimeter scale. This short interaction length is vital for suppressing RF propagation loss, thereby allowing the integrated equalizer to maintain an ideal equalizer response for multi-level modulation. For this reason, we designed a segmented phase shifter with an integrated FIR filter and confirmed that the EO bandwidth increases from 35 GHz to 80 GHz. At the same time, the present modulator achieves the smallest $V_\pi L$ and the lowest drive voltage among those listed in Table 1, while retaining the non-resonant behavior. Following these designs, we have successfully demonstrated the first DSP-free 180-Gb/s PAM4 operation using a Si slow-light modulator. We have also confirmed that the device achieves an extremely low energy consumption of 26 fJ/bit with a CMOS-compatible drive voltage and has achieved 200 Gb/s PAM4 using only low-complexity electronic assistance.

Table 1:

Table 1. Comparison with representative silicon modulators.

Ref.	Type	Phase shifter	DSP-free data rate [Gb/s]	Data rate w/ DSP [Gb/s]	V_{pp} [V]	Driving bit energy† [fJ/bit]	EO f_{3dB} [GHz]	Phase shifter length [mm]	Optical passband [nm]	$V_\pi L$ [V•cm]	Insertion loss [dB]
**This work**	**MZM**	**PCW**	**180 (PAM4)<br>112 (OOK)**	**200 (PAM4), 140 (OOK)<br>11-tap FFE**	**1.5 (PAM4)<br>0.6 (OOK)**	**63 (PAM4)<br>26 (OOK)**	**≈ 80 (N = 3)<br>62 (N = 4)**	**0.25‡ (N = 3)<br>0.32‡ (N = 4)**	**6**	**0.3**	**11.7 (N = 3)<br>15.5 (N = 4)**
[35]	MZM	Bragg-grating	N.R.	400 (PAM4)<br>bi-GRU/T-biGRU ANN equalizer	5	347	90	0.249	7	0.82	10.5
[14]	MZM	Rib	N.R.	350 (PAM4)<br>99FF/99FB-tap DFE	2.7	116	42	1.5	N.R.	1.7	3.6
[38]	MZM	Rib	140 (OOK)	N.R.	5	893	>110	0.9	N.R.	1.62	4.3
[45]	MZM	Rib	N.R.	100 (PAM4), 138 (PAM8)<br>Pre-distortion + VNLE/NWF/BCJR	3.2	569 (PAM4)<br>318 (PAM8)	>35	2	N.R.	0.82	9.6
[46]	MZM	Rib	N.R.	106 (PAM4)<br>Pre-emphasis + 21-tap FIR	4.5	531	7.7	1.6	N.R.	1.02	2.1
[47]	MZM	Rib	N.R.	Net 225 (PAM8)<br>2nd-order VNLE	2.3	72	47	2.5	N.R.	1.35	5.4
[48]	MZM	Rib	40 (PAM4)	46 (PAM4)<br>FFE/NLE/MLSE	1.1, 2.1	611	27	3	N.R.	0.45	N.R.
[49]	MRM	Rib	N.R.	200 (PAM4)<br>21-tap FFE	1.6	6.3<br>(capacitive)	48.6	N.R.	≈ 0.35	0.6	6
[17]	MRM	Rib	N.R.	330 (PAM8), 280 (PAM-4)<br>Pre-distortion/pre-emphasis + PNLE	1.8	3.1<br>(capacitive)	>50<br>(detuned)	N.R.	≈ 0.3	0.63	N.R.
[50]	MRM	Rib	N.R.	170 (PAM4)<br>Pre-emphasis + 33FF/9FB-tap DFE	2.4	N.R.	47	N.R.	≈ 0.51	N.R.	N.R.

† For MZM-based devices, driving bit energy was calculated from the drive voltage and R_T using the method described in Supplementary Information 5. R_T = 50 Ω was assumed when R_T was not reported.

‡ Including the built-in EO equalizer.

Clarity and context

The manuscript is generally clear and technically interesting. However, the central messaging would benefit from a sharper distinction between what is directly demonstrated (EO bandwidth enhancement, high-speed eyes, TDEC/TDECQ results, 200 Gb/s PAM4 with low-complexity Rx FFE), and what is inferred/projected for practical links (deployment-level DSP-free performance, link-budget sufficiency, CPO-level system impact). I recommend revising the wording accordingly unless additional BER and insertion-loss evidence is added.

In the revised manuscript, experimentally demonstrated results, such as the EO bandwidth enhancement and 200-Gb/s PAM4 operation with the 11-tap receiver FFE, are described as directly demonstrated results. In contrast, statements inferred from these results were revised so that they are presented more clearly as inferred/projected implications.

Abstract lines 13–15: By eliminating reliance on power-dissipating electronics, this strategy ~~enables~~ provides a route toward dense multi-Tb/s co-packaged optics in future energy-constrained switching systems.

Main text lines 128–129: The data rate was further extended ~~in practical optical interconnections, with a~~ using a low-complexity 11-tap symbol-spaced FFE at the receiver, indicating the potential for practical optical interconnects.

Main text lines 159–160: The modulator architecture ~~is highly scalable~~ can also be scaled via wavelength division multiplexing.

Main text lines 164–168: In particular, lattice-shifted PCWs have achieved a 35-nm bandwidth covering the C band[43], ~~facilitating~~ which can facilitate 16- to 32-channel integration and increase total capacity from 3.2 Tb/s to 6.4 Tb/s. A single 0.4-mm^2 modulator ~~enables~~ can enable 32-channel integration, totaling only 12.8 mm^2 for high-density packaging, and the power consumption of the entire transmitter is expected to remain below several pJ/bit even after multiplexing.

Figure 4 caption: ~~Data transmission results.~~ Measured optical modulation characteristics.

References

The manuscript cites relevant prior work on slow-light silicon modulators and high-speed silicon photonic links. If the authors continue to emphasize CPO significance, a more explicit quantitative

comparison to representative CPO-oriented modulator approaches would improve context and help clarify the manuscript's distinct contribution.

As described in our response to Suggested improvement 7, we have added Table 1 to compare the present device with representative CPO-relevant silicon modulators, including MZM- and MRM-based devices. This comparison clarifies the position of the present device in terms of EO bandwidth, phase-shifter length, drive voltage, DSP requirement, and non-resonant operation.

References

[R2-1]. Kawahara, K. et al. High-speed, low-voltage, low-bit-energy silicon photonic crystal slow-light modulator with impedance-engineered distributed electrodes. *Optica* **11**, 1212–1219 (2024).

[R2-2]. Codega, N. et al. A Monolithic 400Gbps electro-optical retimer with integrated TIA and class-AB silicon-photonics/VCSEL driver in 5nm FinFET. *Proc. IEEE Symp. VLSI Technol. Circuits*, (2025).

[R2-3]. Gurumoorthy, V. et al. A 212Gb/s PAM-4 Retimer with Integrated High-Swing Optical Driver and Chip-to-Module Long Reach Capability of 40dB in 5nm FinFET. *Proc. IEEE ISSCC*, (2025).

[R2-4]. Kawahara, K. et al. High-efficiency compact optical transmitter with a total bit energy of 0.78 pJ/bit including silicon slow-light modulator and open-collector current-mode driver. *IEEE J. Sel. Top. Quantum Electron.* **32**, 3400111 (2025).

[R2-5]. Nguyen, H. C. et al. Sub-100 μm Photonic Crystal Si Optical Modulators: Spectral, Athermal, and High-Speed Performance. *IEEE J. Sel. Top. Quantum Electron.* **19**, 127–137 (2013).

[R2-6]. Hirotani, K., Shiratori, R. & Baba, T. Si photonic crystal slow-light waveguides optimized through informatics technology. *Opt. Lett.* **46**, 4422 (2021).

Response to the reviewers' comments

The reviewers' comments are enclosed in boxes. The authors' responses are written in blue. The modified parts in the manuscript are written in red, and the retained parts are written in black. Deleted text from the previous manuscript is indicated by ~~strikethrough~~.

Reviewers' comments

Authors' responses

Manuscript modified parts

Manuscript retained parts

~~**Manuscript deleted parts**~~

Response to Reviewer #1:

I would like to thank the authors for their thorough and constructive responses to all the comments from the first round of review. The manuscript has been significantly improved. I have only three minor suggestions for the authors before publication.

We sincerely thank the reviewer for the positive evaluation of our revised manuscript and for the constructive suggestions. We have carefully considered the three remaining comments and have revised the manuscript accordingly. Our detailed responses are provided below.

First, given the extensive amount of experimental data, the authors should carefully double-check the consistency between the text and figures throughout the paper. For instance, in lines 115–116, the text states that the 100G and 112G eye diagrams in Fig. 4a remain clear down to 0.7 V, but the figure actually shows the 100G eye diagram at 0.8 V.

We carefully rechecked the consistency between the text and figures throughout the manuscript and corrected the identified inconsistency.

Main text lines 116–117: As shown in the upper panel of Fig. 4a, clear eye openings at 100 Gb/s and 112 Gb/s were maintained down to V_{pp} = 0.8 V and 0.7 V, respectively.

Second, I suggest that the authors consider replacing the N = 3 data in Fig. 3d with the corresponding N = 4 data. I understand that using the N = 3 device highlights the bandwidth enhancement from the EO equalizer more clearly, but the subsequent DSP-independent experiments are still demonstrated using the N = 4 device. Since readers will naturally be more interested in the actual performance (such as bandwidth and modulation efficiency) of the N = 4 device, presenting the N = 4 data in Fig. 3d would allow for a more direct and coherent comparison.

We agree with the reviewer that the performance of the N = 4 device should be shown more directly because it was used in the subsequent modulation experiments. We have therefore revised Fig. 3d to include both the N = 3 and N = 4 data and standardized the wavelength range of the frequency-response data shown for all devices to 1536–1540 nm in 1-nm steps. In the previous revised manuscript, Fig. 3c showed the equalized response of the N = 4 device at 1535 nm, where the largest f_{3dB} of 62 GHz was obtained. Because this wavelength is close to the PCW band edge and corresponding data were not available for some devices, we replaced it with the 1536-nm data to ensure a consistent and reproducible comparison. Accordingly, the reported maximum f_{3dB} values

for the $N = 4$ device were revised from 62 GHz to 55 GHz. The specific changes are listed below.

Figure 3: Change the wavelength of the $N = 4$ data shown in Fig. 3c from 1535 nm to 1536 nm. Add the results for the $N = 4$ device to Fig. 3d.

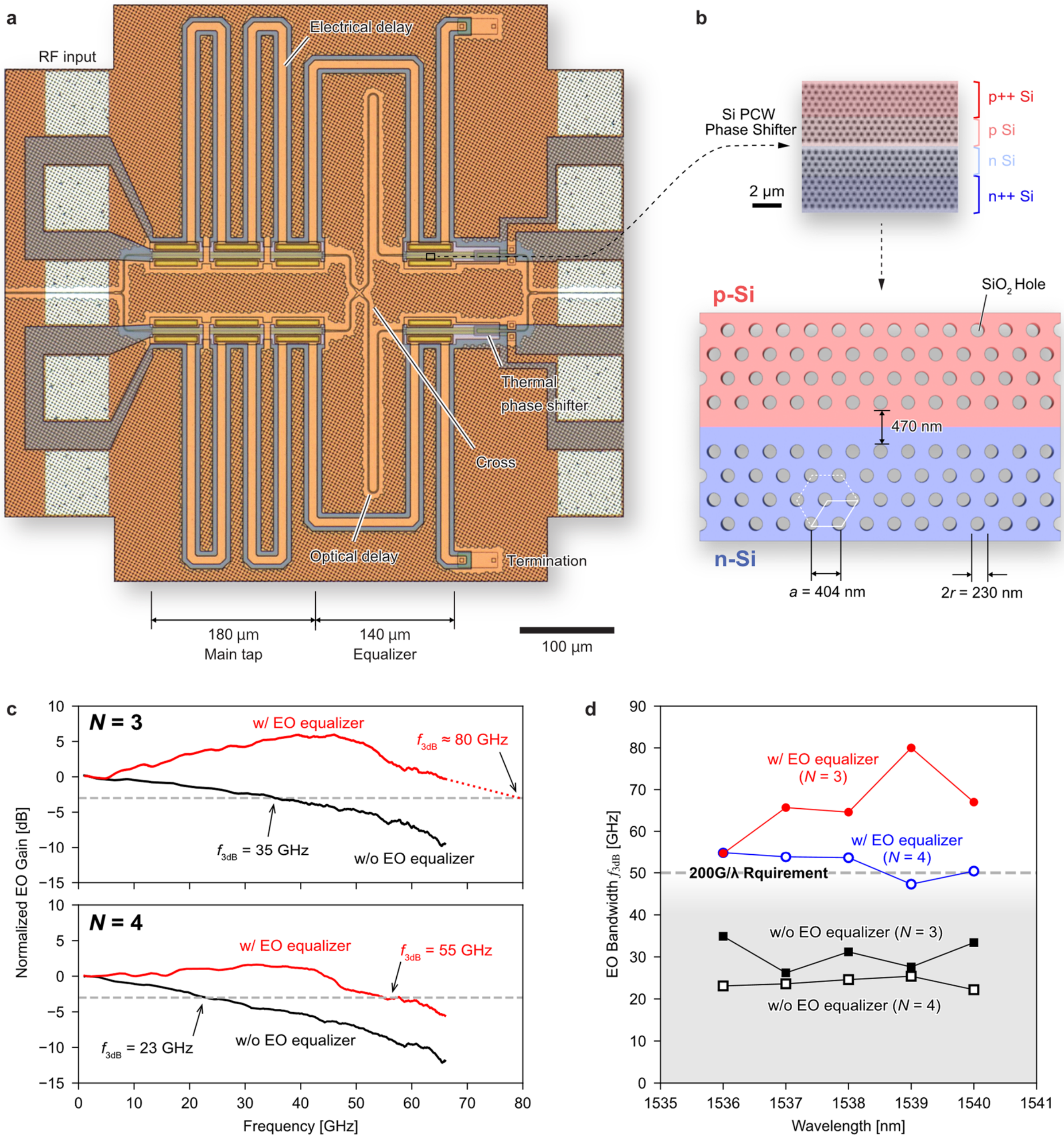

Fig. 3 Fabricated modulator. (a) Optical micrograph. (b) Laser confocal micrograph and an enlarged view of the Si PCW phase shifter. (c) Measured normalized EO gain for $N = 3$ and 4 devices, demonstrating bandwidth enhancement from 35 GHz to 80 GHz for $N = 3$ and from 23 GHz to 55 GHz for $N = 4$ with the EO equalizer. The un-equalized

responses were taken at 1536 nm to minimize the sinc roll-off from inter-segment EO phase mismatch, whereas the equalized responses were taken at the wavelength of maximum f_{3dB} within the 1536–1540 nm range. (d) Measured 3-dB bandwidth of the N = 3 and N = 4 devices across the operating wavelength range of the PCW ~~exceeding the requirement for 200 Gb/s/λ PAM4 transmission~~.

Main text lines 101–106: The N = 4 device provided a more moderate equalization response, increasing the 3-dB bandwidth from 23 GHz to 55 GHz with a flatter EO response. Figure 3d summarizes the measured 3-dB bandwidths of the N = 3 and N = 4 devices for the operating wavelengths of the PCW. Although the frequency response is slightly affected by the n_g dispersion of the PCW, a bandwidth exceeding 50 GHz was observed at almost all the wavelengths, satisfying the Nyquist criterion for 200-Gb/s PAM4 operation.

Table 1: Change the EO f_{3dB} value for the N = 4 device from 62 GHz to 55 GHz.

Accordingly, the wavelength range of the frequency-response data presented in Figs. S3 and S4 of the Supplementary Information was also changed to 1536–1540 nm to match Fig. 3d.

Figures S3 and S4: Change the wavelength range to match Fig. 3d.

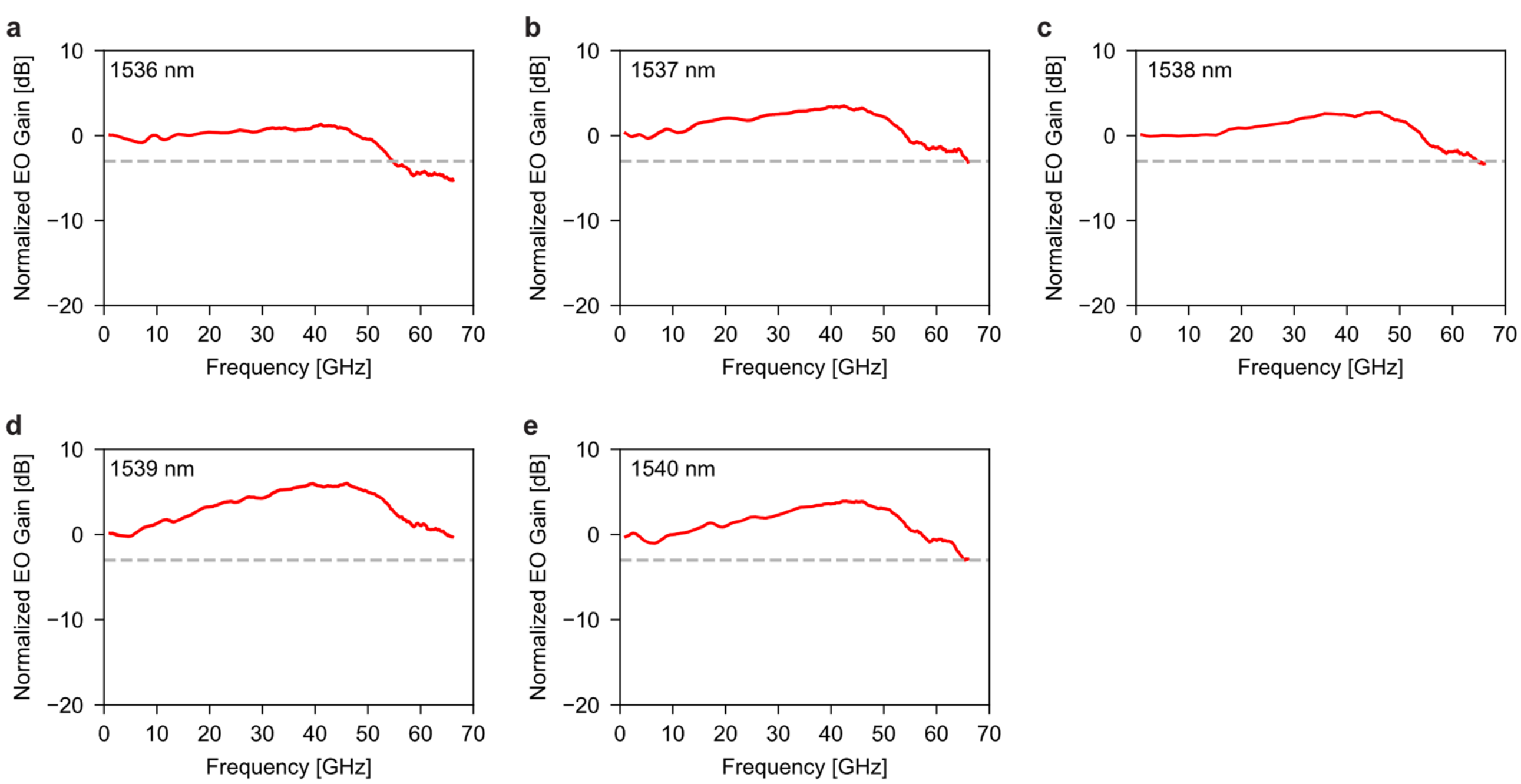

Fig. S3 Measured EO frequency response of the EO equalized PCW modulator (N = 3) for optical wavelengths λ ranging from (a) 1536 nm to (e) 1540 nm. The dashed grey lines indicate the 3-dB bandwidth threshold.

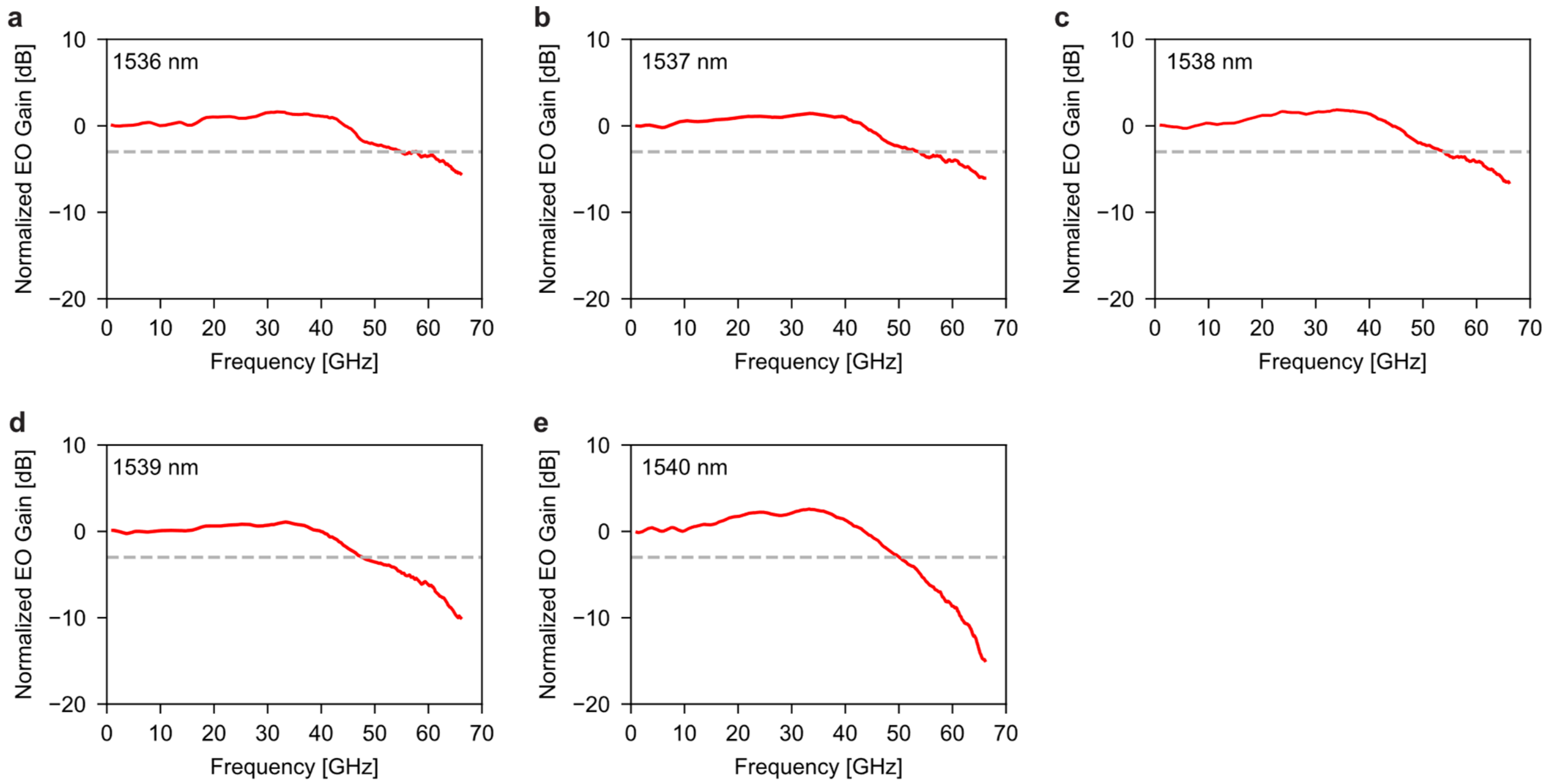

Fig. S4 Measured EO frequency response of the EO equalized PCW modulator (N = 4) for optical wavelengths λ ranging from (a) 1536 nm to (e) 1540 nm. The dashed grey lines indicate the 3-dB bandwidth threshold.

Supplementary information lines 47–48: The f_{3dB} exceeded 50 GHz at almost all the wavelengths for both N = 3 and 4, which supports 200 Gb/s PAM4 operation.

Lastly, please briefly clarify how the driving voltage was adjusted in the experimental setup (e.g., via the AWG output amplitude or by tuning the electrical attenuators). I believe the manuscript will be ready for publication once these minor details are addressed.

The drive voltage was adjusted using the AWG output amplitude, not the electrical attenuators. We have revised the relevant sentence to clarify that all other conditions were kept fixed during this adjustment.

Main text lines 228–229: The drive voltage applied to the modulator was controlled by adjusting the AWG output amplitude while keeping all other conditions fixed.

Response to Reviewer #2:

I thank the authors for a thorough revision. The disclosure of the drive-voltage error, the added comparison table, insertion-loss values, HD-FEC re-analysis, group-delay discussion, and the sharper "DSP-free" vs. "DSP-independent" distinction all strengthen the manuscript.

We sincerely thank the reviewer for the positive evaluation of our revised manuscript and for recognizing the improvements made in response to the previous comments. We have carefully considered the remaining comments and have revised the manuscript accordingly. Our detailed responses are provided below.

One point does not fit the corrected drive voltages. The 140-Gb/s PAM4 drive voltage changed 5.2→2.0 V (ratio 0.385), whereas a uniform correction predicts ≈1.85 V. This discrepancy is beyond rounding and should be explained or corrected.

We apologize for the confusion caused by the incorrect value in the initially submitted manuscript. The apparent discrepancy arises because the value of V_{pp} = 5.2 V for the 140-Gb/s PAM4 signal in the first submitted manuscript was a typographical error and therefore was not a valid value to which the uniform correction could be applied. During the preparation of the first revised manuscript, we checked the original electrical waveform data and recalculated the drive voltage. For the 140-Gb/s PAM4 signal, the peak-to-peak voltage displayed on the DCA was 554.6 mV. Applying the corrected conversion factor of 3.55 gives 554.6 mV × 3.55 = 1.97 V, which was rounded to V_{pp} = 2.0 V. Therefore, the value of V_{pp} = 2.0 V reported in the first revised manuscript is correct and is retained in the current manuscript.

The core claim that a built-in EO equalizer improves transmitter waveform quality on a slow-light Si modulator is correctly and sufficiently supported by the with/without-equalizer TDEC/TDECQ comparison under identical conditions. I accept TDEC/TDECQ as the appropriate metric for that claim and do not require BER for it. However, the manuscript also carries a deployment-level framing (title "DSP-independent 200G/λ," CPO discussion, multi-Tb/s capacity projections) that the present evidence does not establish. Please choose one of the following:

- Scope the claims to transmitter characterization, retaining TDEC/TDECQ and removing or clearly qualifying the link-level and CPO assertions. No new measurements needed, or

- retain the deployment framing, in which case at least one BER-vs-received-power curve is

required. This is needed because TDECQ is anchored to assumed receiver and target-BER conditions, the eyes use averaging that suppresses real receiver noise, and the 200-Gb/s result combines the transmitter with a receiver FFE.

The "DSP-free" vs. "DSP-independent" clarification is part of this same issue. The new definitions are reasonable. The problem is that the title still advertises "DSP-independent 200G/λ," yet the only 200G/λ result with acceptable quality uses receiver DSP; an 11-tap symbol-spaced FFE is DSP. The strong term thus covers precisely the rate at which the DSP-free claim fails. Please remove "DSP-independent" from the 200G/λ framing (e.g., "DSP-light" or "reduced-DSP" would be accurate).

We have revised the manuscript to scope the claims to transmitter waveform characterization. We maintained the TDEC/TDECQ-based evaluation, while revising statements that could imply deployment-level validation beyond the present measurements. Specifically, we qualified the link-level assertions, CPO discussion, and multi-Tb/s capacity projections. These points are now described as future implications from a modulator-design perspective rather than as demonstrated performance. The specific revisions are listed below.

Title: ~~DSP-independent~~ Reduced-DSP 200G/λ low-voltage slow-light silicon modulator enabled by built-in EO equalizer

Abstract lines 12–14: By ~~eliminating~~ reducing reliance on power-dissipating electronics, this strategy provides a potential route toward future dense multi-Tb/s co-packaged optics ~~in future~~ for energy-constrained switching systems.

Main text lines 40–41: In this paper, we present a ~~DSP-independent~~ Si PCW modulator that integrates a built-in EO equalizer to break the bandwidth limitation while preserving the benefits of the slow-light effect, as shown in Fig. 1.

Main text lines 47–49: From a modulator-design perspective, our proposal ~~eliminates~~ offers a path toward reducing reliance on power-dissipating DSPs and drivers~~, providing a path toward~~ in future multi-Tb/s co-packaged optics.

Main text lines 142–143: In this article, we have shown that integrating a built-in EO equalizer with a Si PCW slow-light modulator is ~~essential to~~ effective for overcoming this trade-off ~~without relying~~ while reducing reliance on DSP.

Main text lines 155–156: The achievement of such a high-speed and low-power-consumption modulator platform is important because it ~~eliminates~~ helps reduce reliance on power-intensive DSPs and external drivers.

Main text lines 164–165: The modulator architecture ~~can also be scaled via~~ also provides a basis for future scaling through wavelength division multiplexing.

Main text lines 167–175: The available wavelength range can be broadened in future implementations either by integrating multiple PCW modulators, each designed to operate over a different wavelength range, or by using lattice-shifted PCWs with a broader spectral window. In particular, lattice-shifted PCWs have achieved a 35-nm bandwidth covering the C band[43], ~~which can facilitate~~ suggesting a possible route to 16- to 32-channel integration ~~and increase total capacity from~~ with a projected total capacity of 3.2 Tb/s to 6.4 Tb/s. ~~A single 0.4-mm2 modulator can enable 32-channel integration, totaling only~~ Scaling the 0.4-mm^2 modulator footprint to 32 channels would correspond to a projected total modulator area of 12.8 mm^2 ~~for high-density packaging~~, and the power consumption of the entire transmitter ~~is expected to~~ is projected to remain below several pJ/bit even after multiplexing. We believe that this architecture offers promise as a ~~viable solution~~ building block for future co-packaged optics in high-density data centers.

Figure 1a: Change "DSP-independent" to "Reduced-DSP."

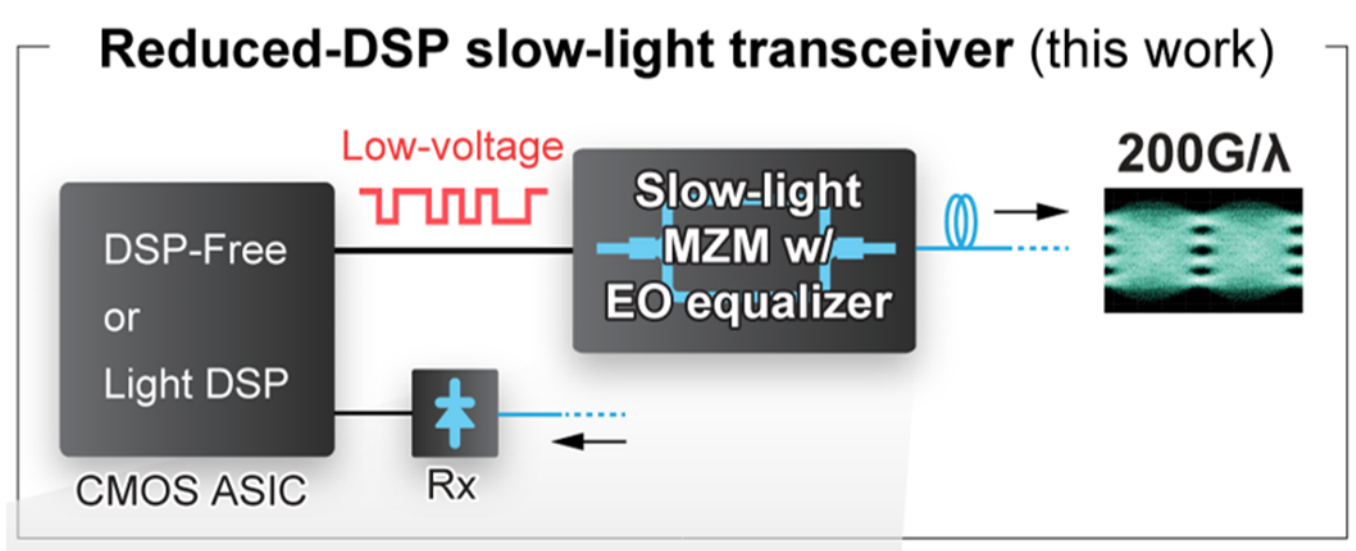

Figure 1 caption: Concept of the ~~DSP-independent~~ reduced-DSP slow-light transceiver.

Figure 5 caption: Experimental setups. (a) Small-signal EO response measurement setup consisting of a VNA and an LCA. (b) ~~Data transmission~~ High-speed modulation measurement setup using an AWG and a sampling oscilloscope for evaluating ~~DSP-independent~~ reduced-DSP performance.

The reported 80 GHz bandwidth is obtained from the $N = 3$ device by linear-in-dB extrapolation beyond the 67 GHz measurement limit, while all modulation data come from the $N = 4$ device, which shows a measured bandwidth of 62 GHz. The two-device rationale is reasonable, but the

abstract highlights the 35 to 80 GHz improvement from a device with no modulation data. Since the revision now includes the $N = 4$ response, the modulation discussion should highlight the 62 GHz result and clearly identify which device each value refers to.

We agree that the 35-to-80-GHz enhancement of the $N = 3$ device should not be emphasized in the Abstract and general discussion when the modulation experiments were performed with the $N = 4$ device. We therefore removed this numerical comparison and now describe the equalized EO bandwidth in terms of exceeding the Nyquist bandwidth required for 200-Gb/s PAM4 operation.

Abstract lines 7–9: By monolithically integrating optical delay lines and crossings within segmented slow-light phase shifters, we synthesized an on-chip feedforward response that extends the 3-dB bandwidth ~~from 35 GHz to 80 GHz~~.

Main text lines 41–44: The equalizer compensates for the intrinsic roll-off with a high-pass response to extend f_{3dB} ~~beyond the Nyquist limit~~. We demonstrated a Si modulator with a compact device length, while extending the ~~intrinsic~~ equalized EO bandwidth ~~from 35 GHz to 80 GHz~~ beyond the Nyquist bandwidth required for 200-Gb/s PAM4.

Main text lines 147–148: For this reason, we designed a segmented phase shifter with an integrated FIR filter and confirmed that the EO bandwidth increases ~~from 35 GHz to 80 GHz~~ beyond the Nyquist bandwidth.

To ensure consistency of the frequency-response data throughout the manuscript, we added the $N = 4$ data to Fig. 3d and standardized the wavelength range to 1536–1540 nm in 1-nm steps. In the previous revised manuscript, the 62-GHz value was obtained at 1535 nm, which is close to the PCW band edge and for which data were not available for some devices. The $N = 4$ response in Fig. 3c was therefore replaced with the 1536-nm data, and the corresponding wavelength range in Figs. S3 and S4 was revised to match Fig. 3d. As a result, the $N = 4$ bandwidth is reported as 55 GHz rather than 62 GHz in the present revision, and this value is used consistently throughout the manuscript. The specific changes are listed below.

Main text lines 101–106: The $N = 4$ device provided a more moderate equalization response, increasing the 3-dB bandwidth from 23 GHz to 55 GHz with a flatter EO response. Figure 3d summarizes the measured 3-dB bandwidths of the $N = 3$ and $N = 4$ devices for the operating wavelengths of the PCW. Although the frequency response is slightly affected by the n_g dispersion of the PCW, a bandwidth exceeding 50 GHz was observed at almost all the wavelengths, satisfying

the Nyquist criterion for 200-Gb/s PAM4 operation.

Table 1: Change the EO f_{3dB} value for the $N = 4$ device from 62 GHz to 55 GHz.

Figure 3: Change the wavelength of the $N = 4$ data shown in Fig. 3c from 1535 nm to 1536 nm. Add the results for the $N = 4$ device to Fig. 3d.

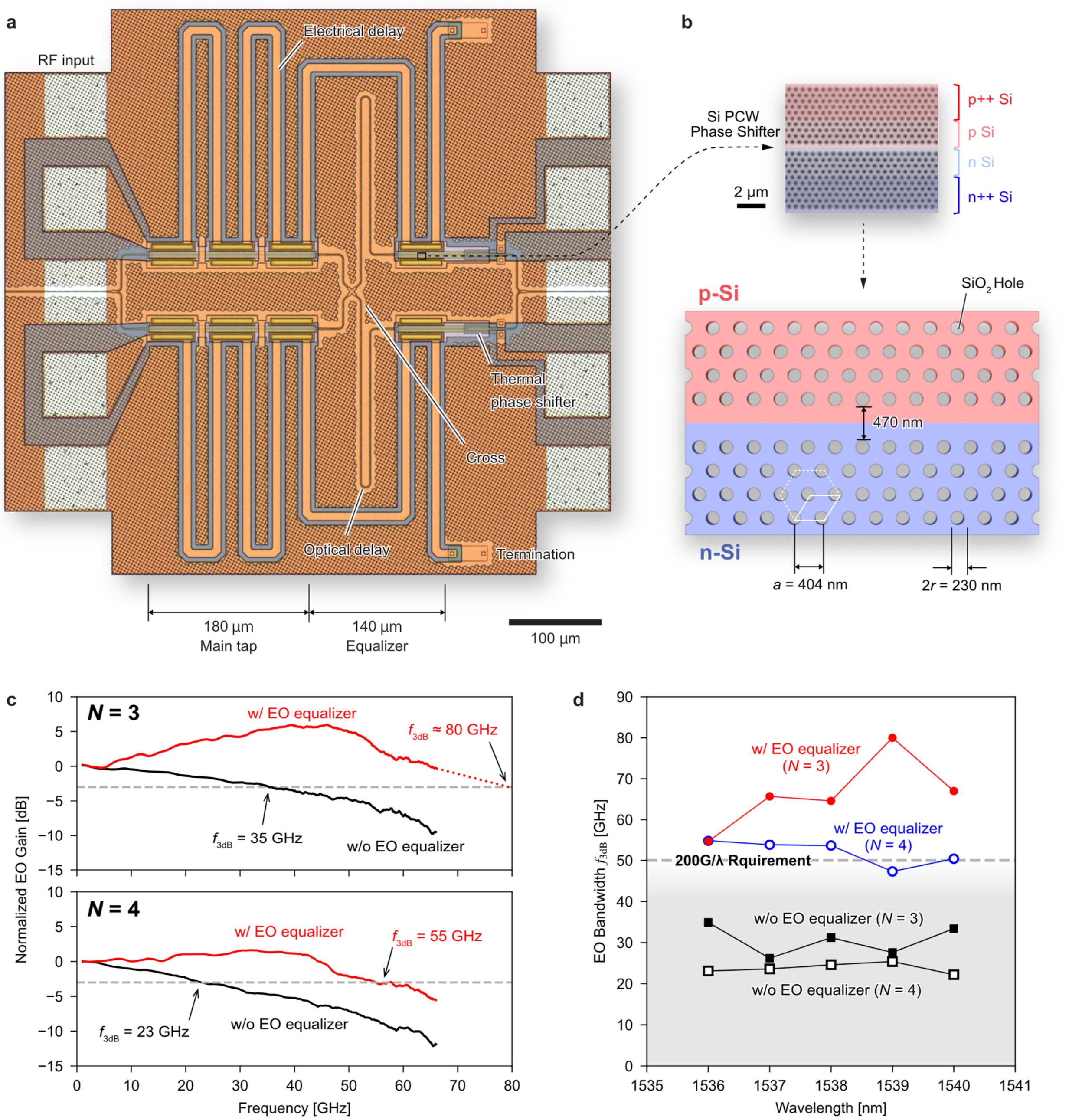

Fig. 3 Fabricated modulator. (a) Optical micrograph. (b) Laser confocal micrograph and an enlarged view of the Si PCW phase shifter. (c) Measured normalized EO gain for $N = 3$ and 4 devices, demonstrating bandwidth enhancement

from 35 GHz to 80 GHz for N = 3 and from 23 GHz to 55 GHz for N = 4 with the EO equalizer. The un-equalized responses were taken at 1536 nm to minimize the sinc roll-off from inter-segment EO phase mismatch, whereas the equalized responses were taken at the wavelength of maximum f_{3dB} within the 1536–1540 nm range. (d) Measured 3-dB bandwidth of the N = 3 and N = 4 devices across the operating wavelength range of the PCW ~~exceeding the requirement for 200 Gb/s/λ PAM4 transmission~~.

<u>Figures S3 and S4</u>: Change the wavelength range to match Fig. 3d.

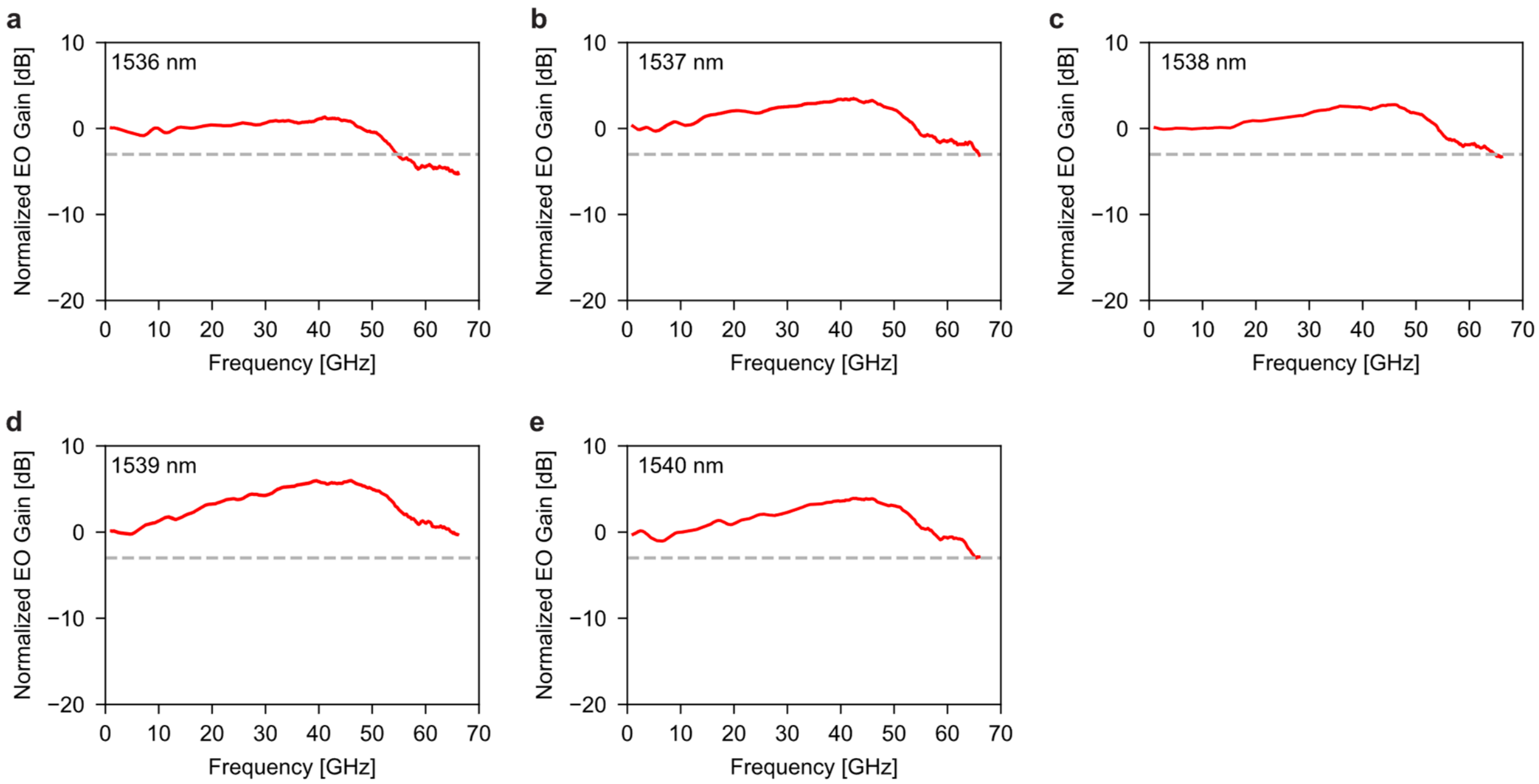

Fig. S3 Measured EO frequency response of the EO equalized PCW modulator (N = 3) for optical wavelengths λ ranging from (a) 1536 nm to (e) 1540 nm. The dashed grey lines indicate the 3-dB bandwidth threshold.

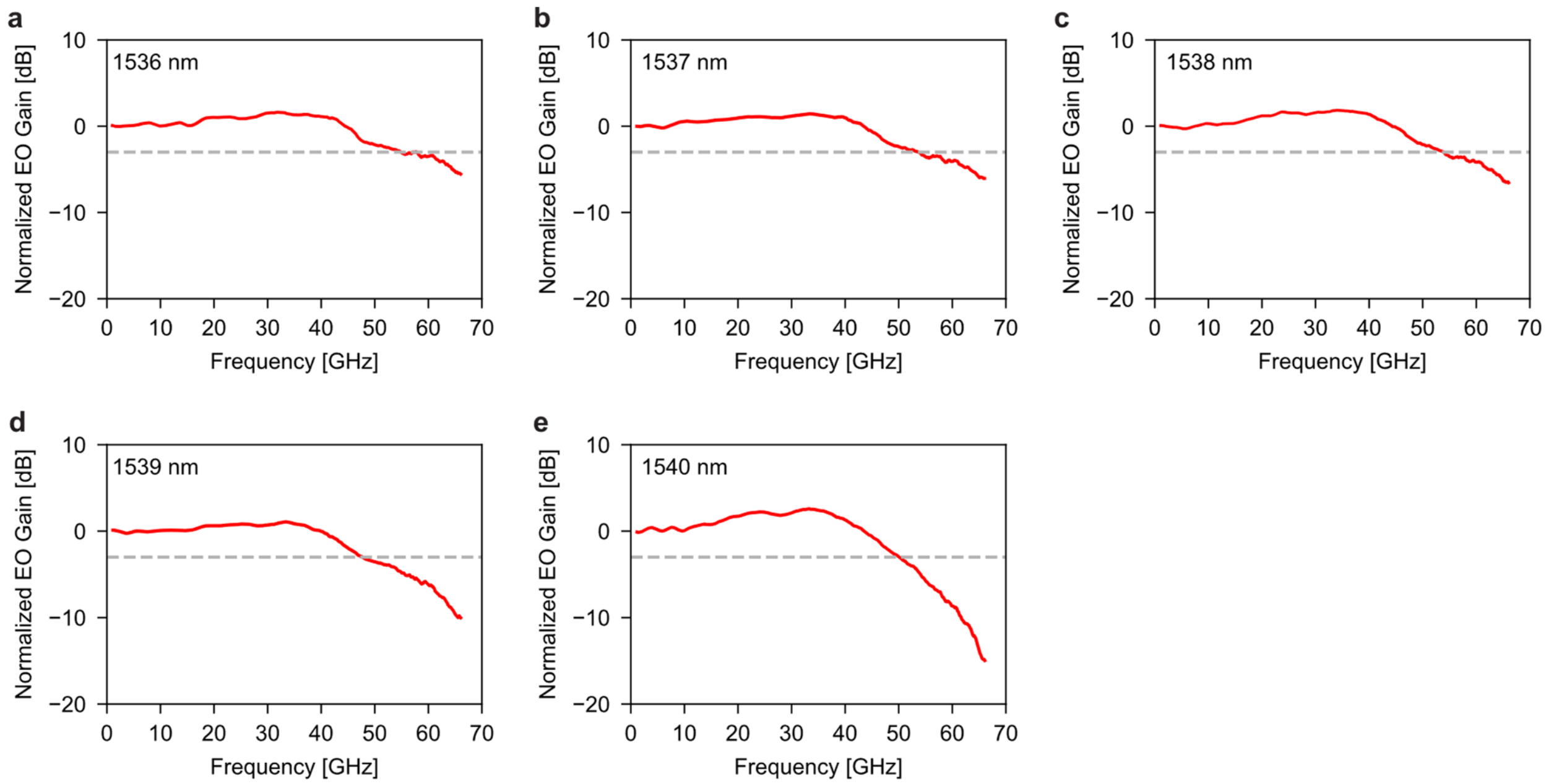

Fig. S4 Measured EO frequency response of the EO equalized PCW modulator (N = 4) for optical wavelengths λ ranging from (a) 1536 nm to (e) 1540 nm. The dashed grey lines indicate the 3-dB bandwidth threshold.

The measured on-chip loss (up to 15.5 dB for $N = 4$) requires a downstream EDFA in the present setup, which is in tension with the CPO claims. A brief, explicit link-budget statement or at least an acknowledgment that CPO viability is contingent on reducing this loss would close the gap.

We agree that the insertion loss should be explicitly connected to the co-packaged-optics discussion. The downstream EDFA in the present setup compensates the optical loss and should not be interpreted as part of an amplifier-free co-packaged transmitter. We have therefore revised the Discussion to state that the present 11.7–15.5 dB insertion loss must be reduced for co-packaged-optics implementation so that the transmitter can satisfy the optical power budget without downstream optical amplification.

Main text lines 161–164: At the same time, the present devices exhibit an insertion loss of 11.7–15.5 dB (see Supplementary Information 1), which was compensated by an erbium-doped fiber amplifier (EDFA) in the measurement setup. For future implementation in co-packaged optics, this loss needs to be reduced.
